# Supplementary material for: Cytochrome P450 Enzyme Design by Constraining the Catalytic Pocket in a Diffusion Model
Source: Research (Wash D C). 2024 Jul 8;7:0413. doi: 10.34133/research.0413 (PMC11227911; doi:10.34133/research.0413)
Supplement: Supplementary 1 — Supplementary Information Figs. S1 to S25 Tables S1 to S4 [file research.0413.f1.zip › Revised_Supplementary_Material_clean_version.docx]

Supplementary Information for

**Cytochrome P450 Enzyme Design by Constraining Catalytic Pocket in Diffusion Model**

Qian Wang^1,2,3†^, Xiaonan Liu^1,2,3†^, Hejian Zhang^1,3,8†^, Huanyu Chu^1,2†^, Chao Shi^4†^, Lei Zhang^1,5^, Jie Bai^1,3^, Pi Liu^1,3^, Jing Li^1,3,6,7^, Xiaoxi Zhu^1,2,3^, Yuwan Liu^1,3^, Zhangxin Chen^4^, Rong Huang^1,3^, Hong Chang^1,3^, Tian Liu^1,3^, Zhenzhan Chang^4*^, Jian Cheng^1,3*^, Huifeng Jiang^1,3*^

*^1^Key Laboratory of Engineering Biology for Low-Carbon Manufacturing, Tianjin Institute of Industrial Biotechnology, Chinese Academy of Sciences, Tianjin, 300308, China;*

*^2^University of Chinese Academy of Sciences, Beijing, 100049, China;*

*^3^National Center of Technology Innovation for Synthetic Biology, Tianjin, 300308, China;*

*^4^Department of Biochemistry and Biophysics, School of Basic Medical Sciences, Peking University, Beijing, 100191, China;*

*^5^College of Life Science and Technology, Wuhan Polytechnic University, Wuhan, Hubei, 430023, China;*

*^6^State Key Laboratory of Elemento-Organic Chemistry, College of Chemistry, Nankai University, Tianjin, 300071, China;*

*^7^College of Life science, Nankai University, Tianjin, 300071, China;*

*^8^College of Biotechnology, Tianjin University of Science and Technology, Tianjin, 300457, China.*

†These authors contributed equally to this article.

*Correspondence: Huifeng Jiang (jiang_hf@tib.cas.cn), Jian Cheng ([cheng_j@tib.cas.cn](mailto:cheng_j@tib.cas.cn)) and Zhenzhan Chang (changz@bjmu.edu.cn).

## Supplementary Methods

### Chemicals and media

Yeast nitrogen base without amino acids and ammonium sulfate (YNB), Bacto peptone, Bacto yeast extract, Luria Broth (LB), agar, lithium acetate, ssDNA and glucose were obtained from Solarbio, China. Kanamycin, ampicillin, amino acids, adenine, histidine, leucine, tryptophan and uracil were obtained from Sigma-Aldrich, USA. Chromatography-grade methanol and acetonitrile were obtained from EMD chemicals, USA. Chromatography-grade formic acid and isopropanol were obtained from Thermo Fisher Scientific, USA. Authentic reference standard of scutellarein and apigenin was obtained from Solarbio, China. The Hi-Fusion Cloning Mix was purchased from CWBIO, China and used for recombinant plasmid construction. *S. cerevisiae* W303-1B (*MATα leu2-3112 ura3-1 trp1-92 his3-11,15 ade2-1 can1-100*)(1) was used as the parent strain for all engineered strains. Competent cells of *E. coli* DMT (TransGen Biotech, China) were used for recombinant vectors construction. Competent cells of *E. coli* BL21(*DE3*) (TransGen Biotech, China) was used for recombinant protein expression. *E. coli* was grown in LB medium with appropriate antibiotics. Yeast strains were grown in CM medium minus tryptophan or uracil and was used for 24-well plates and shake-flask fermentation of yeast. The corresponding solid plate was added 15 g/L of agar powder.

### Bacterial expression and purification of the P450 enzyme

Nucleotide sequence of ancX3 was codon optimized for *E. coli* and codons for the N-terminal transmembrane domain was replaced with codons for an optimized sequence of a short hydrophilic peptide “AKKTSSKGK”, and codons for 6 × His-tag were inserted before the stop codon to facilitate purification. The gene was subcloned into pCW_ori+_ expression vector between restriction sites *Nde*I and *Xba*I. The gene of recombinant construct was synthesized by Genscript, China. Expression and puriﬁcation of recombinant protein were conducted as the methods reported by Gu *et al*(2). The purity and subunit molecular masses of the recombinant ancX3 was verified by SDS-PAGE analysis, and the protein concentration was determined using a BCA Protein Assay Kit (Pierce, USA) with 2 mg/mL BSA as the standard. Purified enzymes were stored at -80 °C before use.

### Fluorescence microscopic analysis

The nucleotide sequences of Design6444, Design11361, Design33380, Design49566, Design58683, Design84497, Design91808 and CYP706X1 were fused with green fluorescent protein (GFP) at C-terminal and subcloned between GAL1 promoter and CYC1 terminator of pYES2.0. The plasmids were verified by sequencing and transformed into W303-1B, and transformants were selected on a uracil minus plate (CM-Ura). The cells were grown at 30 ℃ and 550 rpm for 24 hours, after which the resulting seed cultures were transferred into fresh medium and induced by galactose for 12 hours. The cultivation was diluted twice and used for microscopic analysis. Images were acquired using a Leica DM5000B microscope (Leica, Germany) with a 100 × objective and Leica filter GFP for fluorescent microscopy. Leica LAS AF software (Leica, Germany) was used for image acquisition.

### HPLC detection

The culture samples were diluted with an equal volume of 100% methanol. After vigorous mixing and ultrasonic breaking for 30 min, the lysates were spun down at 13,000 × g for 10 min. The supernatant compounds were measured at 335 nm using a Kinetex 5 µm Biphenyl 100 Å LC Column (250 × 4.6 mm; Phenomenex, USA) operating at 30 ℃. The mobile phase consisted of 0.1% formic acid and acetonitrile with methanol at a flow rate of 1 mL·min-1 using the following gradients: 0-20 min, 22% acetonitrile, 5% methanol; 20 min to 22 min, acetonitrile increased from 22% to 90%; 25 min, 22% acetonitrile. Subsequently, the column was washed and equilibrated for 5 min before the next injection. 30 µL of the sample were injected into the HPLC system and each run was stopped at 30 min after the injection.

### Soluble expression and the spectral analysis of P450 designs expressed in *E. coli*

The P450 candidate genes was codon optimized for *E. coli*. The N-terminal transmembrane domains were predicted by TMHMM 2.0 and replaced with codons for an optimized sequence “MAKKTSSKGK”, and 6× His-tag was added to the C-terminal to facilitate purification. The DNA fragment synthesized and inserted into the NdeI and XbaI sites of the pCWori+ vector by GenScript (Nanjing, China).

The plasmid was transformed into BL21 (DE3) competent cells following the manufacturer’s specification. One fresh colony grown on LB plates containing Amp (100 µg/mL) 50mL of an LB medium containing Amp (100 µg/mL) and incubated at 37°C, 220rpm. Then, 5mL aliquots of overnight culture were inoculated into 500mL of the TB medium containing Amp (100 µg/mL) in the 2L flasks and incubated at 37°C, 220rpm. Approximately 5h later, the protein expression was induced with 0.5mM IPTG and 0.5mM ALA when OD600 reached 0.6−0.8. Incubation was continued with shaking at 32°C, 180rpm. Ampicillin (100 µg/mL) was added in each 24h incubation. The cells were harvested 48h later by centrifugation at 5000g, 4°C for 10min, and stored at −20°C in a freezer.

Harvested cells were thawed in lysis buffer (500 mM potassium phosphate, pH 7.4, 250 mM NaCl, 0.25% sodium cholate, 10% glycerol, 10 mM imidazole, 10 mM β-mercaptoethanol) on ice. Cells were lysed by sonication on ice for 30 min. Cell debris was removed by centrifugation at 4000 rpm, 4°C for 30 min. The supernatant was applied to a 24-channel automatic purifier (Cat. No. P241001) (1) adsorbed by Ni NTA Magarose Beads and washed with (2) buffer A (10 mM potassium phosphate, pH 7.4, 500 mM NaCl, 10% glycerol, 25 mM imidazole, 10 mM β-mercaptoethanol) and (3) buffer B (10 mM potassium phosphate, pH 7.4, 500 mM NaCl, 200 mM imidazole, 10 mM β-mercaptoethanol, 10% glycerol) (4) buffer C (10 mM potassium phosphate, pH 7.4, 500 mM NaCl, 500 mM imidazole, 10 mM β-mercaptoethanol, 10% glycerol). The protein was then eluted with elution buffer (SEC buffer: 10 mM potassium phosphate, pH 7.4, 500mM NaCl, 250mM imidazole, 10% glycerol, 10mM β-mercaptoethanol) by the 24 well thickener plate and analysis by SDS-PAGE. The ligand spectral analysis was conducted by a microplate spectrophotometer (BioTek EON) at the wavelength of 300-600 nm for scanning.

## Supplementary Figures


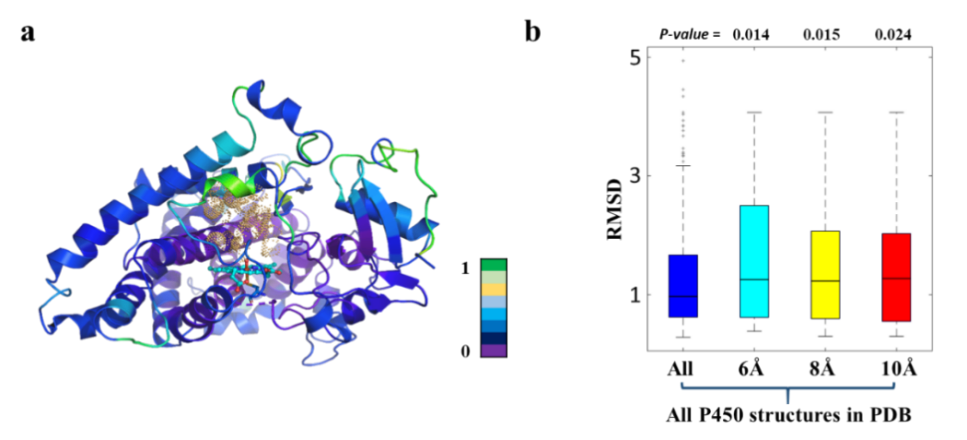


**Figure S1. Structural variability of the catalytic pocket between different P450s in PDB database. (a)** The structural alignment of non-redundant P450 structures in the PDB database reveals varying levels of structural variability among different regions. The color gradient in the legend, from green to purple, signifies decreasing structural variability, with the green region corresponding to the binding pocket area exhibiting the highest variability. **(b)** A box plot illustrates the structural variability (RMSD) at different distances (all, 6Å, 8Å, and 10Å) from the active site. The region within a 6Å distance from the active site displays the most significant variability.


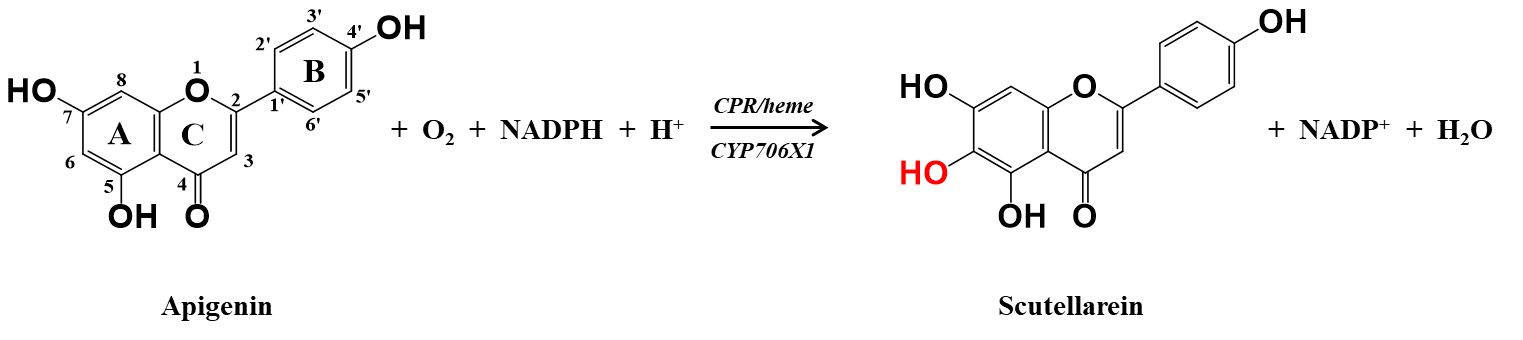


**Figure S2. The CYP706X1 in E. breviscapus catalyzes the 6-hydroxylation of apigenin to yield scutellarein.** The figure depicts the reaction scheme catalyzed by CYP706X1 in conjunction with CPR (cytochrome P450 reductase) and heme.


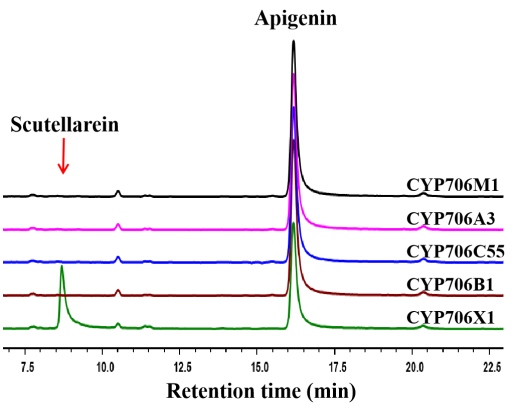


**Figure S3. HPLC analysis of the fermented products catalyzed by various cytochrome P450 enzymes (CYP706X1: green, CYP706B1: brown, CYP706C55: blue, CYP706A3: purple, CYP706M1: black).** Among these enzymes, only CYP706X1 exhibits reactivity towards the substrate apigenin, leading to the formation of the product scutellarein. The substrate apigenin and the product scutellarein are clearly labeled in the figure.


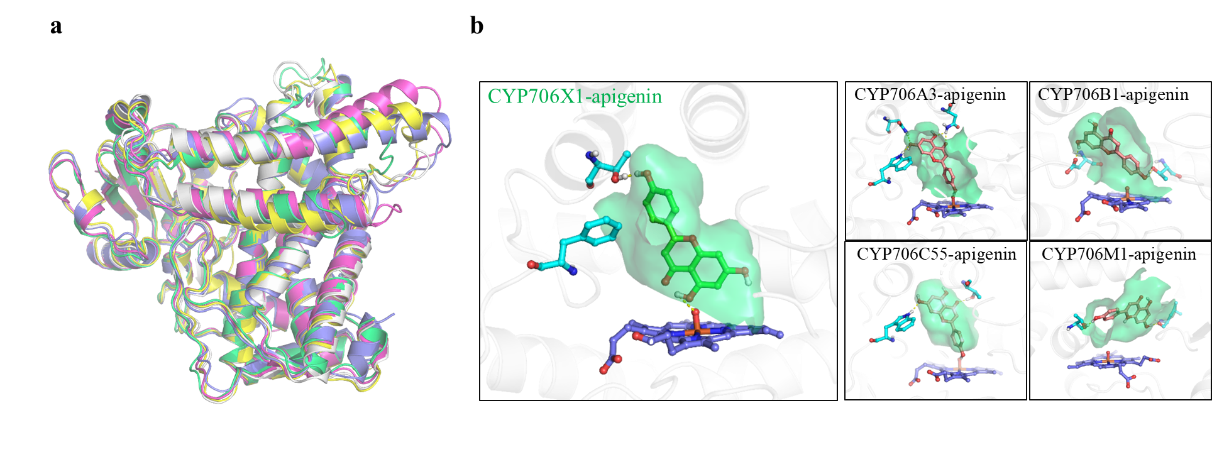


**Figure S4. The structural alignment and apigenin-binding models of CYP706X1, CYP706B1, CYP706C55, CYP706A3 and CYP706M1. (a)** The structural alignment of the five P450 enzymes reveals a unique structural arrangement, with each enzyme represented in a different color. **(b)** The structural models depict the binding of apigenin within these five P450 enzymes. The substrate apigenin and critical residues are illustrated as ball-and-stick models, while the green regions delineate the shapes of the substrate-binding domains. Dashed lines signify hydrogen bond interactions. All residues in the figures are colored cyan, with the apigenin in CYP706X1 shown in green, and brown in the other four proteins.


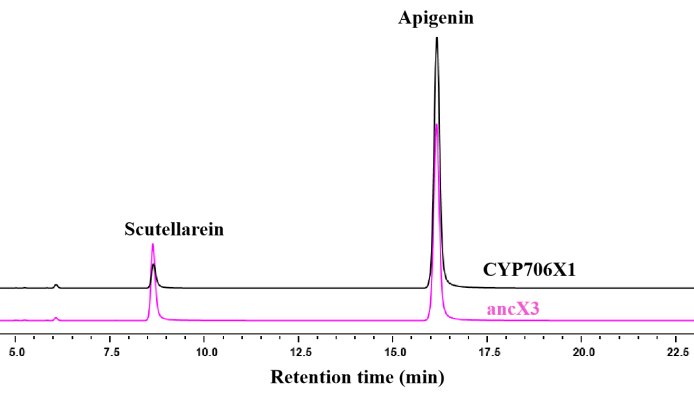


**Figure S5. HPLC analysis of the fermented products catalyzed by CYP706X1 (black) and ancX3 (magenta).** CYP706X1 and ancX3 exhibit reactivity towards the substrate apigenin, leading to the formation of the product scutellarein. The ancX3 shows a higher level of catalytic capability compared to CYP706X1. The substrate apigenin and the product scutellarein are labeled in the figure.


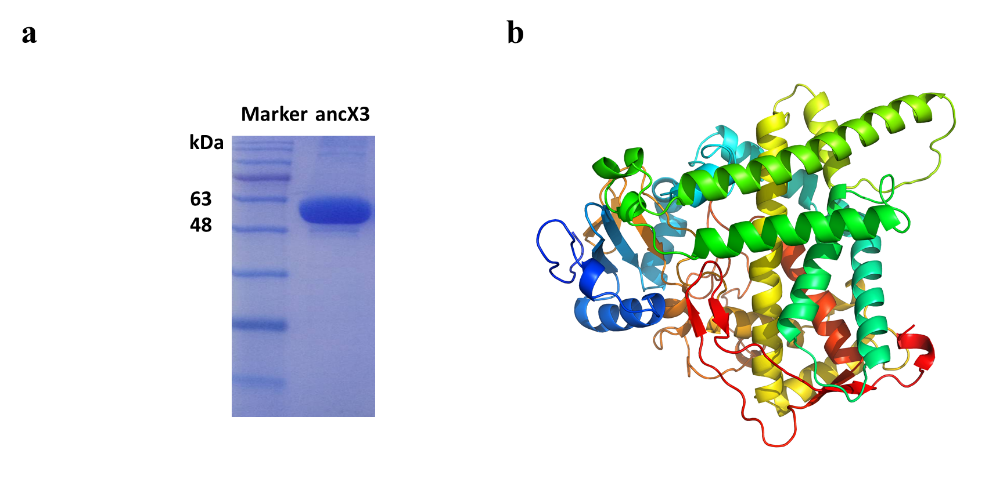


**Figure S6. SDS-PAGE analysis of the purified ancX3 and the crystal structure of ancX3.** **(a)** SDS-PAGE analysis of the purified ancX3 and showed in the second gel lane. The molecular weight of protein ancX3 is 53.90 kDa. **(b)** The structural representation of the ancX3 crystal structure is shown as a cartoon model and the crystal resolution is 2.3Å.


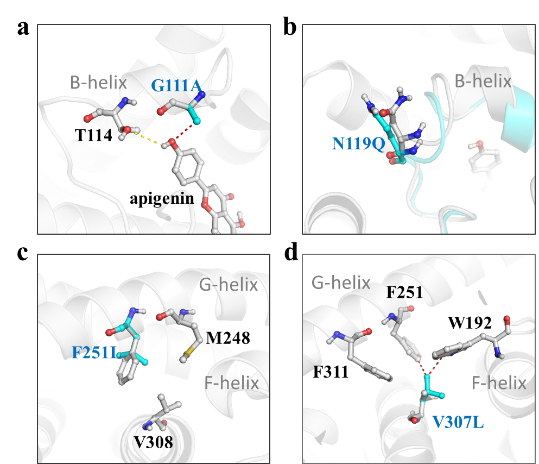


**Figure S7. Structural analysis of four mutations (i.e., G111A, N119Q, F251L and V307L) in ancXY-16.** **(a)** The G111A mutation introduced a collision between the side chain of alanine and substrate apigenin thus impairing the activity of ancXY-16. **(b)** The N119Q was far from the active center, and the mutation might influence the hydrophilicity of the protein surface. **(c and d)** The F251L and V307L changed the local hydrophobic property of ancXY-16. Ball-and-stick models are used to represent the substrates and residues. Wild-type residues are colored white and labeled in black, while mutations are colored cyan and labeled in blue.


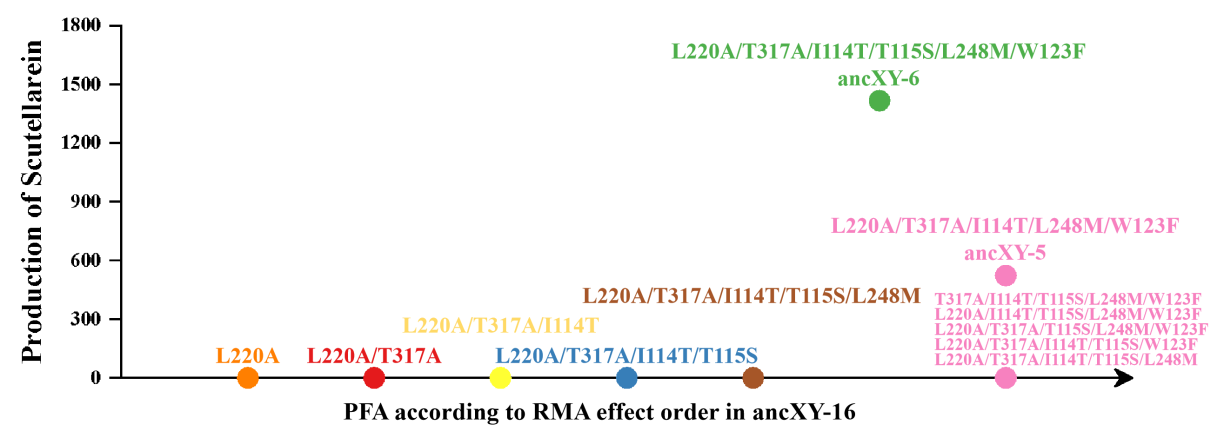


**Figure S8. Progressive forward accumulation (PFA) process for accumulating crucial residues in F6H functional innovation.** PFA process followed the order of RMA mutation effects in ancXY-16. The mutant ancXY-6 (green) within the PFA process displayed initial indication of F6H functional innovation.


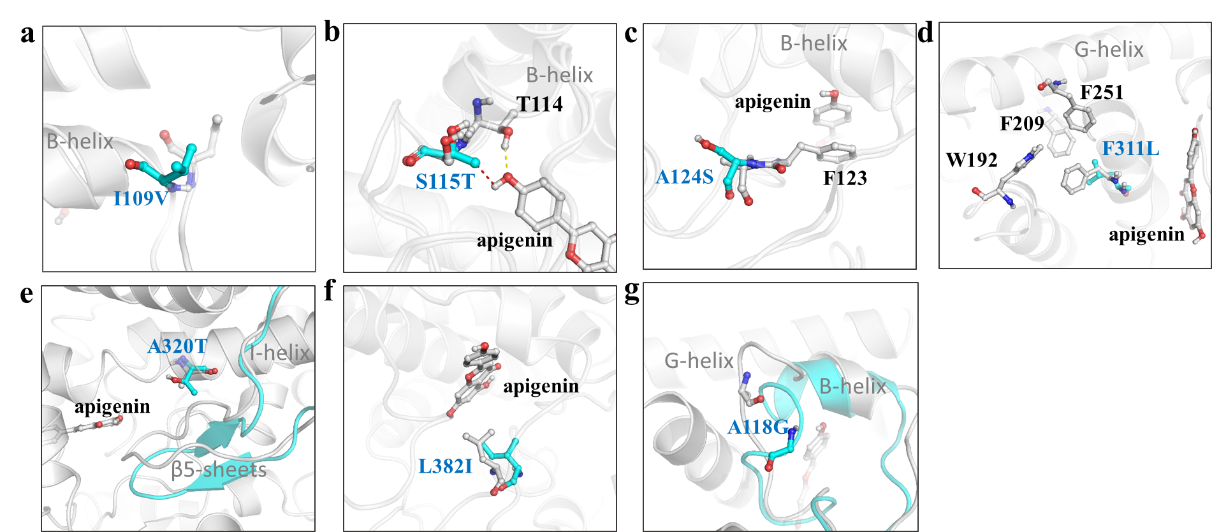


**Figure S9. Structural analysis of seven mutations (i.e., I109V, S115T, A124S, F311L, A320T, L382I and A118G) in ancXY-12.** **(a, d and f)** The I09V, F311L and L382I changed the local hydrophobic properties of ancXY-12 to influence the catalytic activity. **(b)** The S115T introduced space collision between the side chain of threonine and substrate apigenin, which impaired the activity of ancXY-12 severely. **(c and e)** The A124S and A320T are far from substrate apigenin, and may influence the hydrophilic environment of ancXY-12. **(g)** The A118G affected the main-chain conformation of the B-helix by introducing a turn in front of the B-helix. Ball-and-stick models are used to represent the substrates and residues. Wild-type residues are colored white and labeled in black, while mutations are colored cyan and labeled in blue.


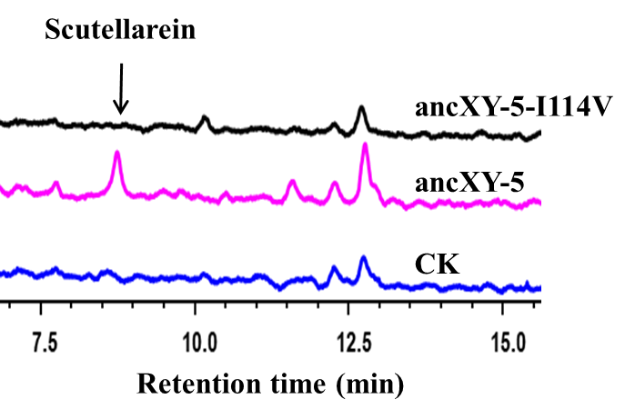


**Figure S10. HPLC analysis of the fermented products catalyzed by ancXY-5 (magenta) and ancXY-5-T114V mutation (black).** The T114V mutation deactivated the catalytic activity of ancXY-5. The control check is colored blue and the scutellarein is indicated with a black arrow.


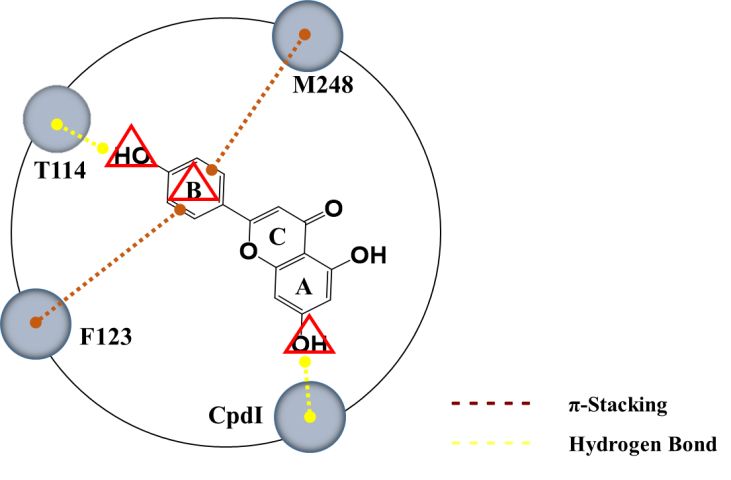


**Figure S11. The representative diagram of “three-point fixation” model.** Three pivots in apigenin are marked with red triangles, while yellow and brown dash lines represent hydrogen bond and π-stacking interactions, respectively. Key residues and CpdI in this model are represented by gray circles.

**Figure S12. Proposed possible flavone 6-hydroxylation reaction mechanism.** The oxyferryl species of CpdI attacks the π-system of the “A” ring of apigenin to produce cationic σ-complex or radical σ-complex and then occurs ring closure to produce epoxide. The epoxide undergoes a hydride shift and enol isomerization to gain scutellarein. The mechanism speculation is based on a previous study(3).


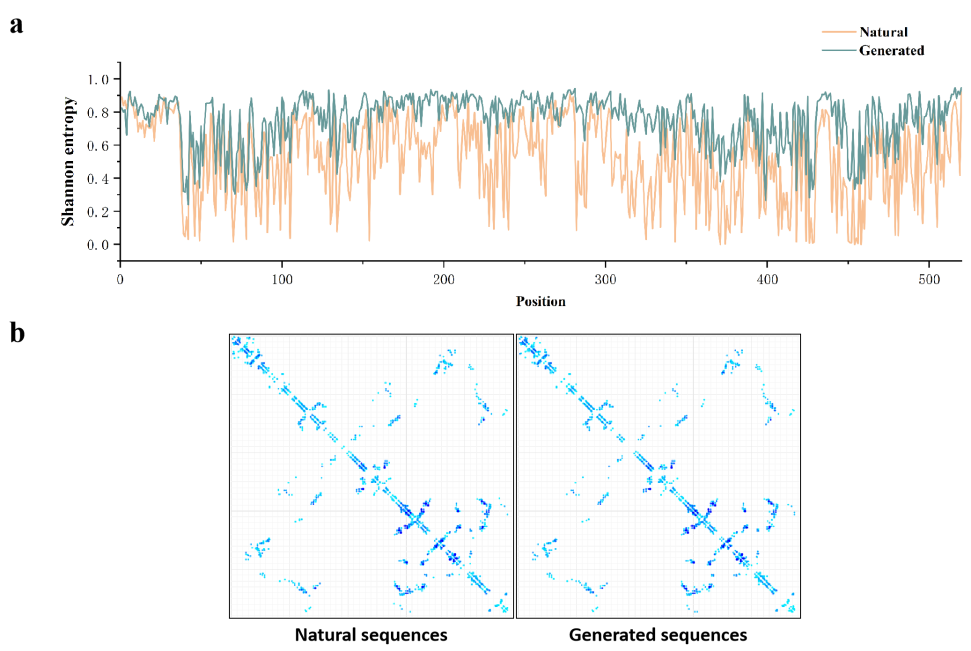


**Figure S13. The evaluation of P450Diffusion fine-tuning model generated sequences. (a)** The amino acids variations of generated sequences (orange) and natural sequences (green) at each position are expressed as Shannon entropy. Low Shannon entropy values represent highly conserved positions and high entropy values indicates high amino-acid diversity at a given position. **(b)** The coevolution feature of natural sequences (left) and generated sequences (right). The coevolution analysis was performed using GREMLIN software4, and the multiple sequence alignments (MSAs) for natural sequences and generated sequences were used as input files, respectively.


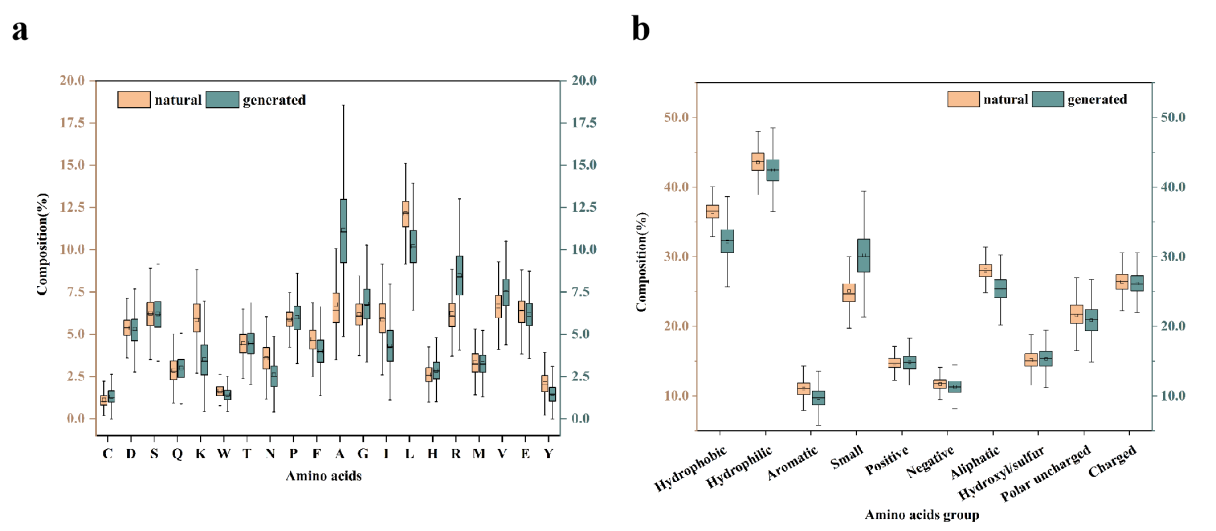


**Figure S14. The amino acid composition of raw sequences and generated sequences. (a)** A boxplot displays the percental amino acid composition of raw sequences, grouped by their physicochemical properties. **(b)** The amino acid distribution and compositional variability of sequences generated by P450Diffusion are highly similar to those of the natural sequences. P450Diffusion is able to replicate the specific physicochemical properties found in natural sequences.


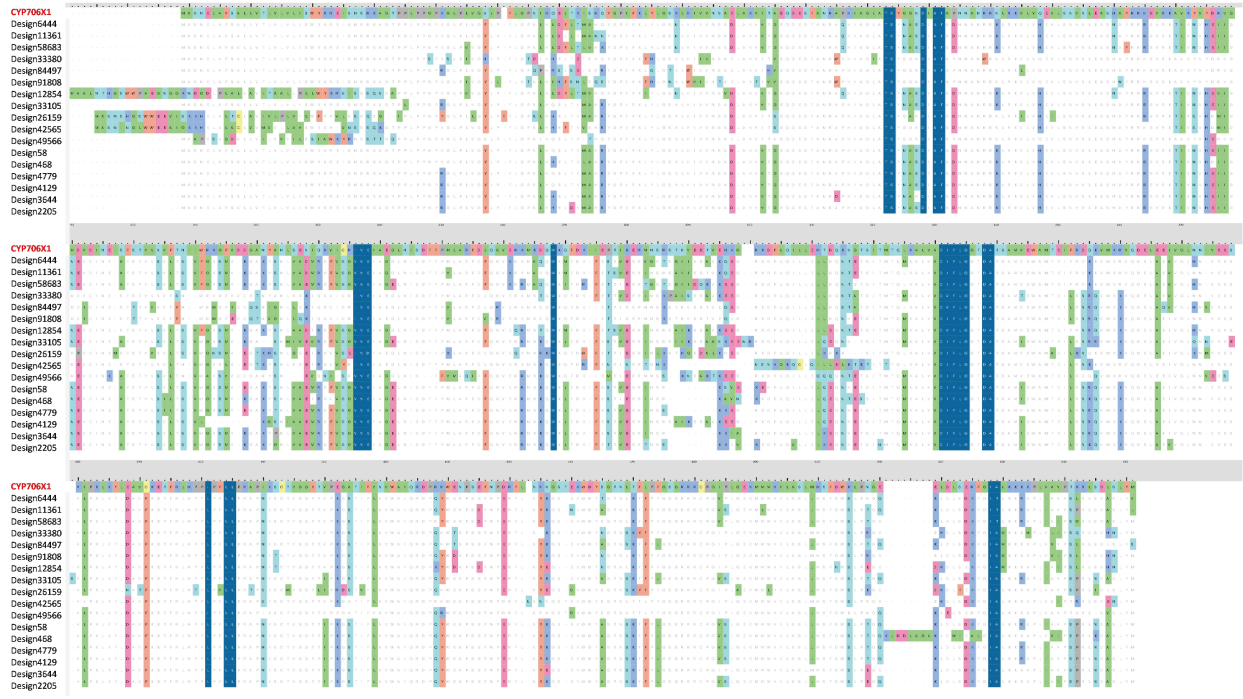


**Figure S15. The multiple sequence alignment of the generated designs with CYP706X1 reveals nearly identical configurations in the protein-substrate binding pockets.** Residues within a 4-angstrom distance of the catalytic pocket are highlighted in deep blue, while identical aligned residues are denoted by dots.


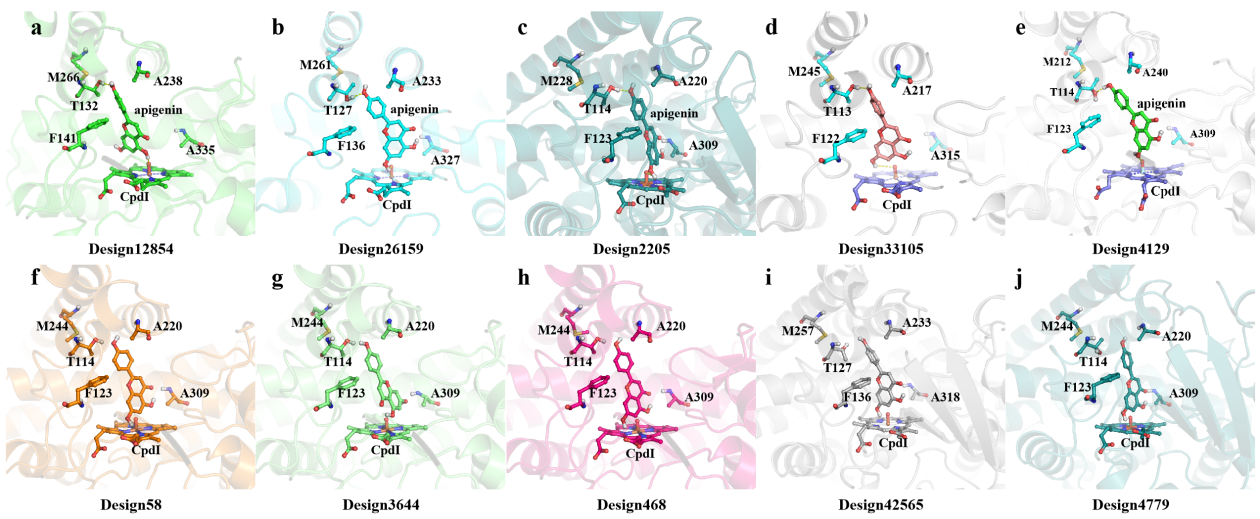


**Figure S16. The apigenin binding model of 10 active designs.** Substrates in active designs bind to catalytic pockets in a manner highly similar to natural CYP706X1. The cartoon of ten designs is colored. Key residues and heme species were labeled in figures too.

.


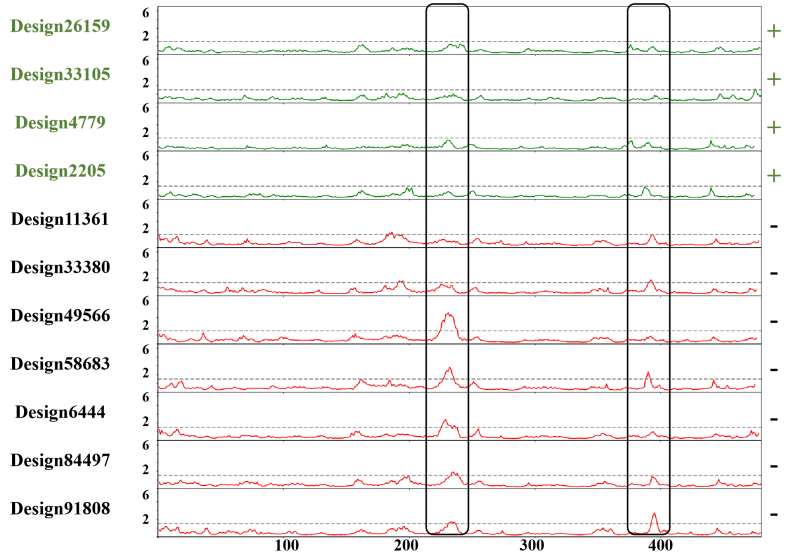


**Figure S17. Root Mean Square Fluctuation (RMSF) curve of the designed P450s (N-terminal trimmed) in molecular simulations.** This curve illustrates the average fluctuations of each residue within the designed P450s, reflecting their flexibility and stability during the simulation. Higher peaks typically correspond to flexible regions, while lower peaks indicate more stable regions. Four active designs, including Design26159, Design33105, Design4779, and Design2205 were selected as active controls and highlighted in green. Additionally, seven inactive designs, consisting of Design11361, Design33380, Design49566, Design58683, Design6444, Design84497 and Design91808, have been marked in red.


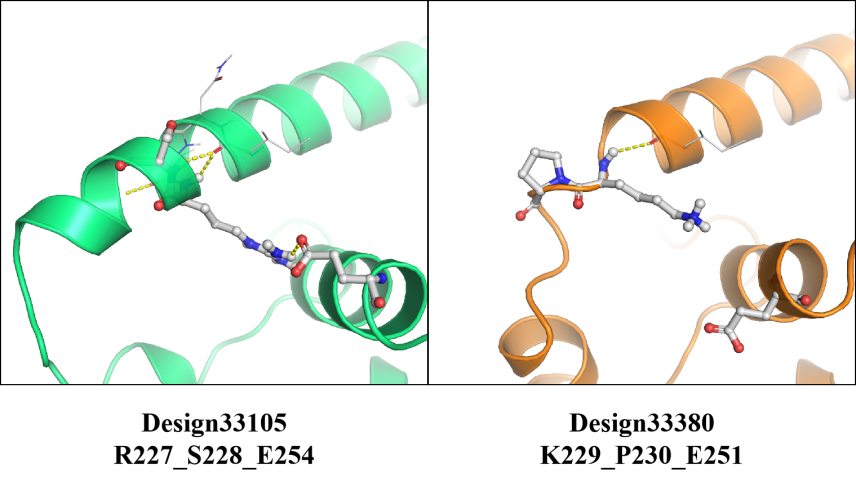


**Figure S18. The local fluctuations in region 220-230 (N-terminal trimmed) of active Design33105 (green) compared to inactive Design33380 (brown).** The mutations were represented as stick-and-ball model at both designs. The salt-bridge between R227 and E254 in active Design33105 are broken in inactive Design33380.


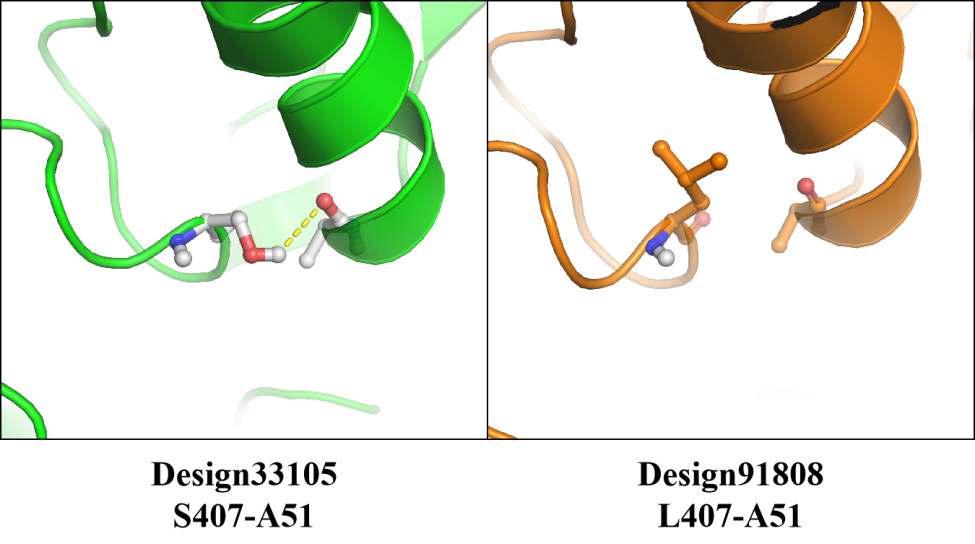


**Figure S19. The local fluctuations in region 390-410 (N-terminal trimmed) of active Design33105 (green) compared to inactive Design91808 (brown).** The mutations were represented as stick-and-ball model at both designs. The hydrogen bond between S407 and A51 in active Design33105 are broken in inactive Design91808.


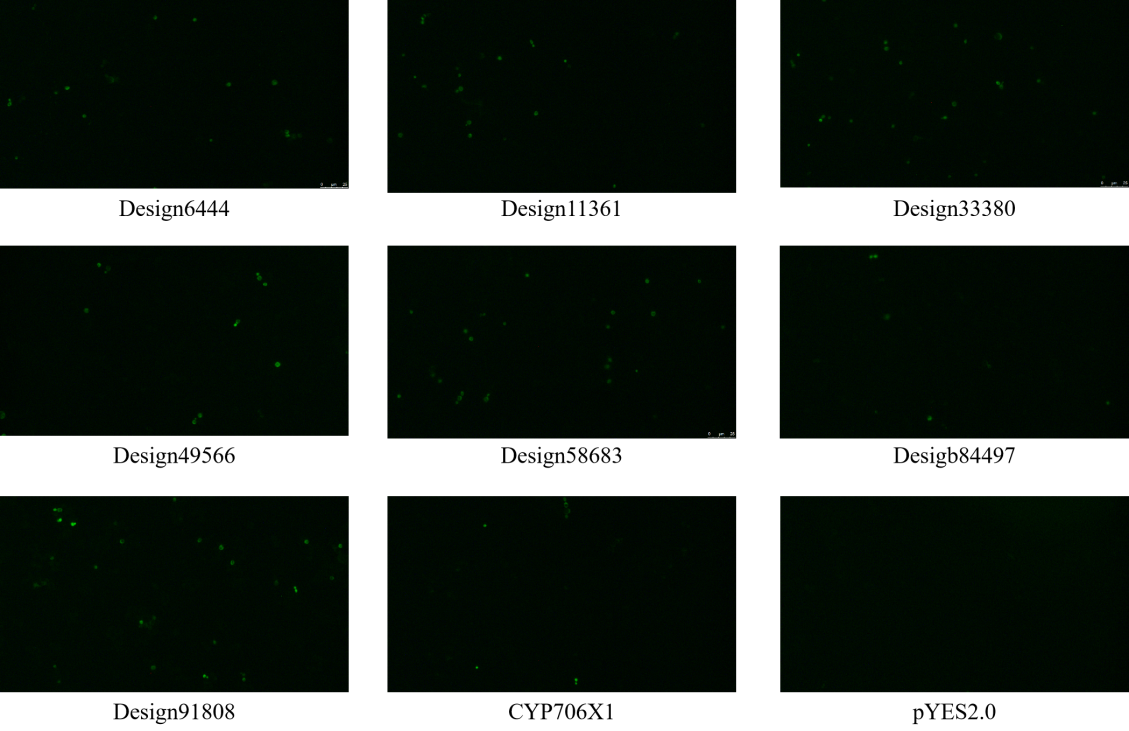


**Figure S20. Fluorescence microscopic analysis of inactive designs generated by deep learning.** Design6444, Design11361, Design33380, Design49566, Design58683, Design84497 and Design91808 were fused with GFP and then visualized by fluorescence microscopy. The pYES2.0-CYP706X1(EbF6H) was used as positive control. The pYES2.0 was used as negative control. The green dots represent correctly expressed and folded P450s tagged with green fluorescent proteins.


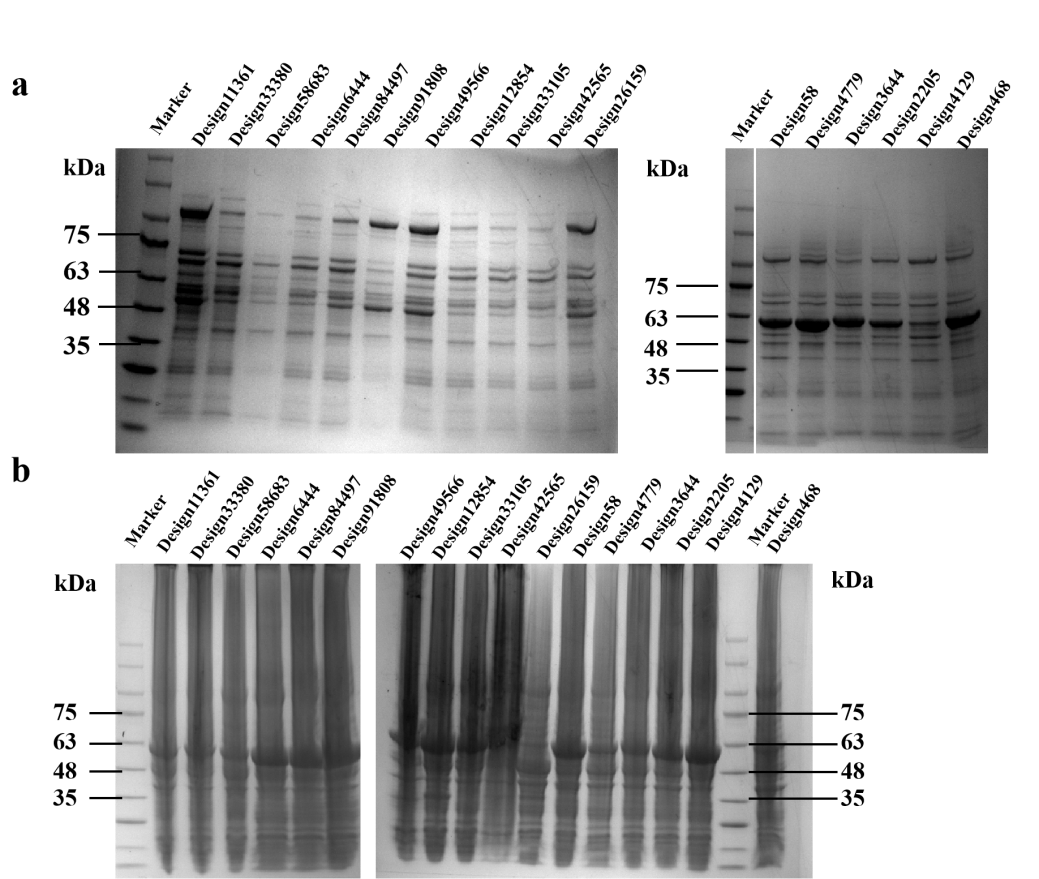


**Fig. S21 SDS-PAGE analysis of the P450 enzymes expressed in *E. coli* system.** (a) SDS-PAGE analysis of the P450 enzymes soluble expression in *E. coli* eluted by 200 mM imidazole. (b) SDS-PAGE analysis of the P450 enzymes precipitation expression in *E. coli*.


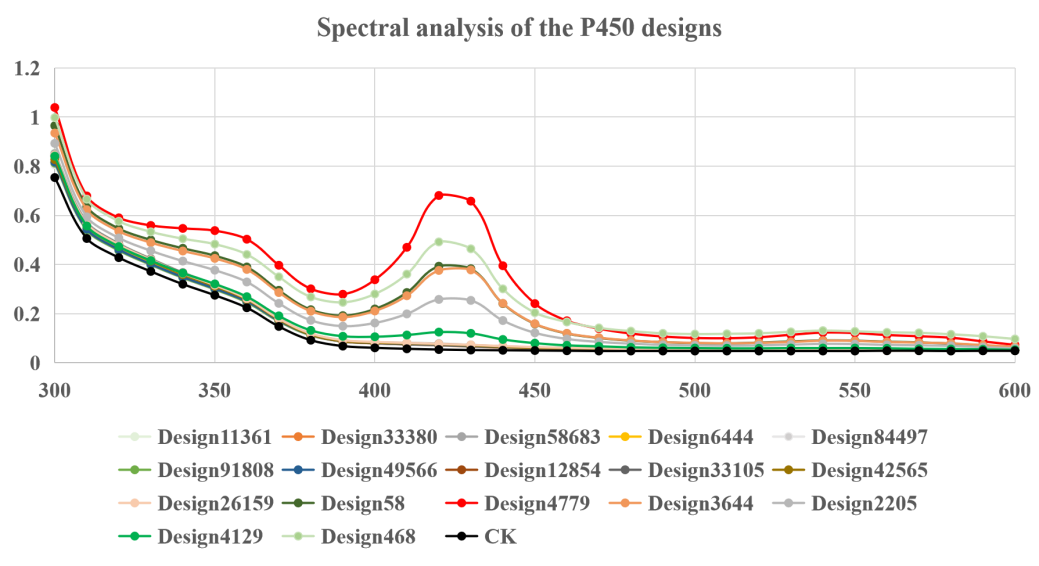


**Figure S22. Spectral analysis of the P450 designs expressed in *E. coli* system.** The horizontal x-axis is the absorption wavelength of 300-600 nm and the vertical Y-axis is the OD value.


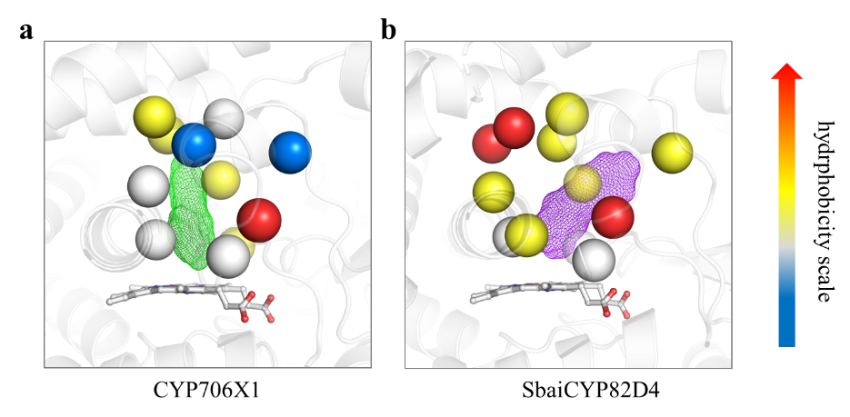


**Figure S23. Comparative analyses of the substrate-binding modes between CYP706X1 and SbaiCYP82D4.** A “vertical binding mode” and an “oblique binding mode” were found in CYP706X1 **(a)** and SbaiCYP82D4 **(b)**, respectively. Residues in the catalytic pockets and heme molecules are represented as spheres and ball-and-sticks, respectively. The gradient from blue to red represents increasing hydrophobicity. The substrates in CYP706X1 and SbaiCYP82D4 are represented as mesh, and colored green and purple, respectively. The cartoon representations of both proteins were colored white.


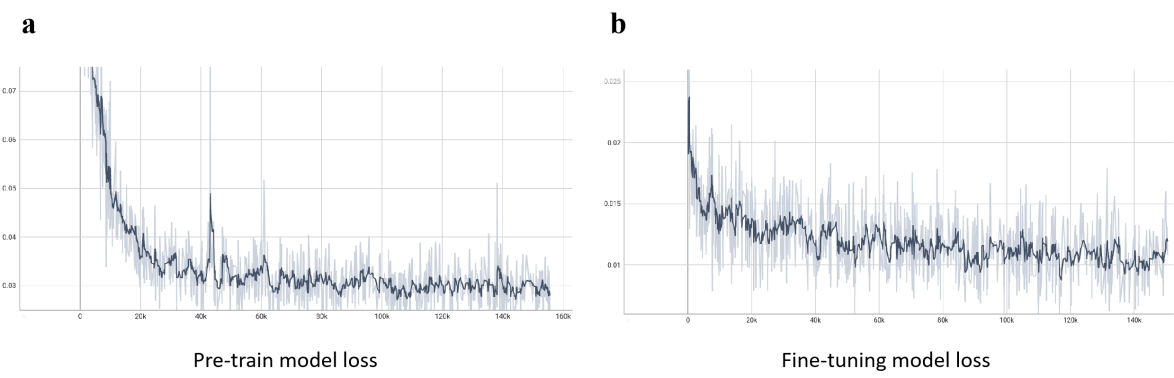


**Figure S24. The losses of the P450diffusion Pre-trained model (a) and Fine-tuning model (b) in the training procedure.** The loss of the training process is smoothed in the figure.


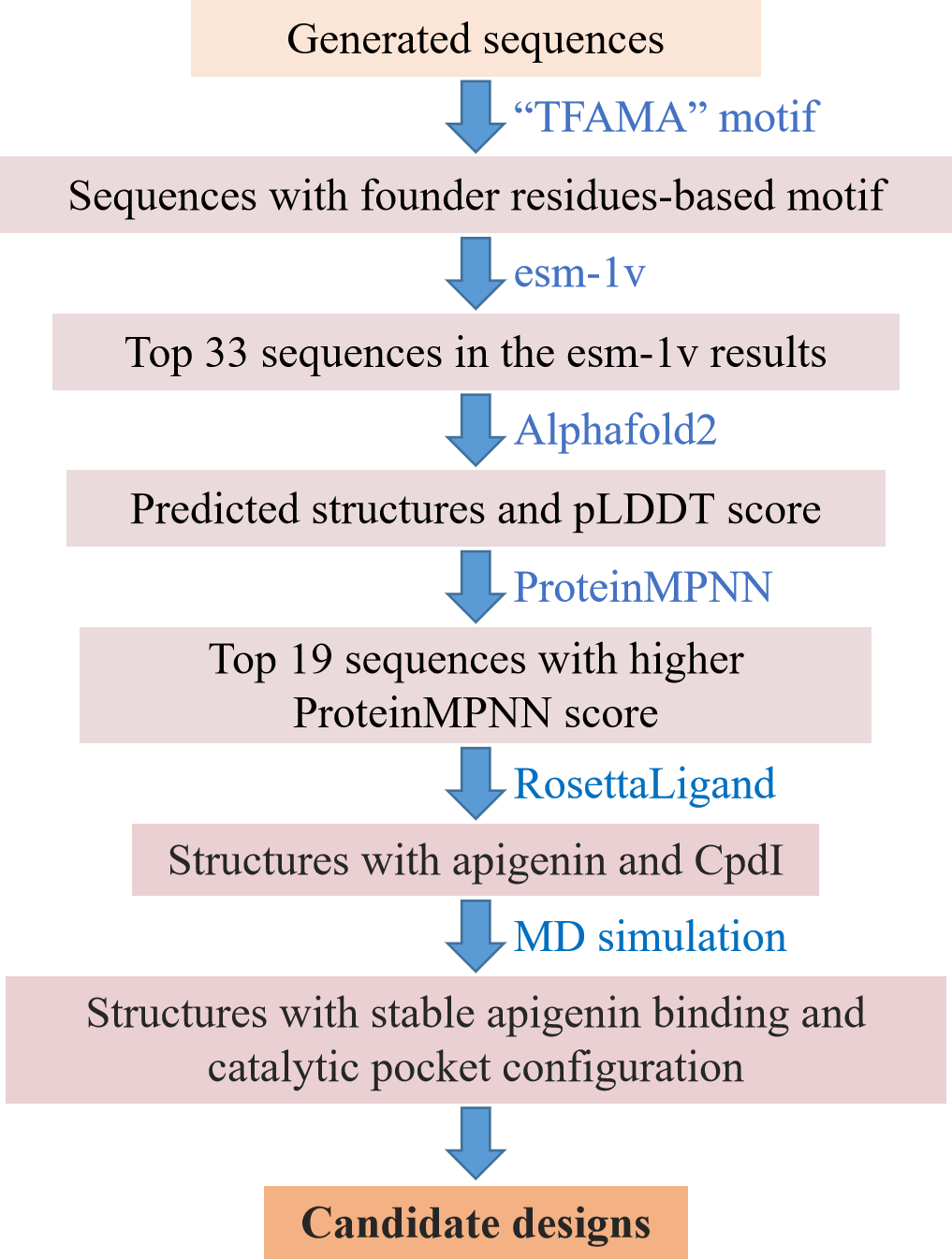


**Figure S25. The computational evaluation and structure-based virtual screening for generated sequences.** The virtual screening protocol is shown as a flowchart.

## Supplementary Tables

**Table S1. Data collection and refinement statistics of ancX3**

| **Data collection** |  |
| --- | --- |
| Beamline | SSRF17U1 |
| Integration Package | XDS |
| Wavelength (Å) | 0.97918 |
| Space group | I432 |
| Unit cell: a, b, c (Å)，α, β, γ (°) | 197.61 197.61 197.61 90.00 90.00 90.00 |
| Resolution (Å) | 46.58-2.30 (2.38-2.30) |
| Rmerge | 0.134 (3.349) |
| Rmean | 0.136 (3.374) |
| Rpim | 0.015 (0.406) |
| Mean((I)/sd(I)) | 30.8 (2.0) |
| CC(1/2) | 1.000 (0.705) |
| Completeness (%) | 100 (100) |
| Total number of measured reflections | 2283864 |
| Total number of unique reflections | 29446 |
| Multiplicity | 77.6 (68.6) |
| Mean(Chi^2) | 1 (0.96) |
| Molecules in an asymmetric unit | 1 |

*Numbers in parentheses are for the highest resolution shell.

**Table S2. Sequence similarity of 17 designs compared to CYP706X1.**

| **Design** | **Sequence similarity** | **Activity** |
| --- | --- | --- |
| Design33380  Design91808  Design84497  Design49566  Design42565  Design11361  Design6444  Design58683  Design4779  Design3644  Design4129  Design58  Design26159  Design2205  Design33105  Design468  Design12854 | 83.8  83.6  84.3  86.2  80.8  77.5  77.5  76.9  77.1  77.8  76.7  77.3  71.3  75.9  75.9  75.5  72.8 | No  No  No  No  Yes  No  No  No  Yes  Yes  Yes  Yes  Yes  Yes  Yes  Yes  Yes |

**Table S3. The primers used in this study.**

| **Primers** | **Primer sequences** |
| --- | --- |
| Y22-Gibson-5F | tcatgtaattagttatgtcacgcttac |
| Y22-Gibson-3R | ATAAAGCAATCTTGATGAGGATAATG |
| PGK1+TDH3-Gibson-5F | tgtttttatatttgttgtaaaaagtagata |
| PGK1+TDH3-Gibson-3R | TTTGTTTGTTTATGTGTGTTTATTCG |
| ATR2-Gibson-5F | GAATAAACACACATAAACAAACAAAATGtcctcttcttcttcttcgtc |
| ATR2-Gibson-3R | TTATCCTCATCAAGATTGCTTTATttaccatacatctctaagatatcttc |
| ancX-Gibson-5F | actttttacaacaaatataaaaacaATGGCTTCTAACGAATTGGCTTTTTCA |
| ancX-Gibson-3R | agcgtgacataactaattacatgaTTACATGTACAAAGAAGCATCTGGTAATCT |
| ancX-Gibson-5F | actttttacaacaaatataaaaacaATGGCTTCTAACGAATTGGCTT |
| ancX-Gibson-3R | agcgtgacataactaattacatgaTTACATGTACAATGAAGCATCAGA |
| ancX1-Gibson-5F | actttttacaacaaatataaaaacaATGGCTTCTAACGAATTGGCTT |
| ancX1-Gibson-3R | agcgtgacataactaattacatgaTTACATGTATAATGACAAATCAGACA |
| ancX2-Gibson-5F | actttttacaacaaatataaaaacaATGGCTTCTAACGAATTGGCTT |
| ancX2-Gibson-3R | agcgtgacataactaattacatgaTTACATGTACAATGACAAATCAGACAAT |
| pYES-3R-zhong | CGCTGGTGAAAGTAAAAGATGCTGAAG |
| pYES-5F-zhong | CATCTTTTACTTTCACCAGCGTTTCTGG |
| eGFP-Linker-5F | ggcggcggaatggtgagcaagggcgaggagctgttc |
| pYES-eGFP-3R | AATATTCCCTATAGTGAGTCGTATTA |
| F6H_Design11361-5F | TAGCAGCTGTAATACGACTCACTATAGGGAATATTATGGCTTCTAATGAATTGGCCTTC |
| F6H_Design11361-3R | aacagctcctcgcccttgctcaccattccgccgccCAAGTACAAGGAAGCATCAGACAATC |
| F6H_Design6444-5F | TAGCAGCTGTAATACGACTCACTATAGGGAATATTATGGCTTCTAATGAACTGGCTTTC |
| F6H_Design6444-3R | aacagctcctcgcccttgctcaccattccgccgccAACGTACAAAGAAGCGTCAGACAGT |
| F6H_Design33380-5F | TAGCAGCTGTAATACGACTCACTATAGGGAATATTATGGCTTCTAACGAACTGGCTTTC |
| F6H_Design33380-3R | aacagctcctcgcccttgctcaccattccgccgccCATGTACAAGTTGTGATCGGATAATCTTTGAG |
| F6H_Design84497-5F | TAGCAGCTGTAATACGACTCACTATAGGGAATATTATGGCTTCTAACGAATTAGCCTTTTCTG |
| F6H_Design84497-3R | aacagctcctcgcccttgctcaccattccgccgccAGAGTACAAGGACAAATCTGACAAACG |
| F6H_Design91808-5F | TAGCAGCTGTAATACGACTCACTATAGGGAATATTATGGCTTCCAACGAATTAGCTTTCTC |
| F6H_Design91808-3R | aacagctcctcgcccttgctcaccattccgccgccCATGTACAAGTTGTGGTCCGATAAACG |
| F6H_Design58683-5F | TAGCAGCTGTAATACGACTCACTATAGGGAATATTATGGCTTCCAACGAATTGGC |
| F6H_Design58683-3R | aacagctcctcgcccttgctcaccatttccgccgccCATGTACAGGCTGGCATCAGAT |
| F6H_Design49566-5F | TAGCAGCTGTAATACGACTCACTATAGGGAATATTatggcttctaacgaattggc |
| F6H_Design49566-3R | aacagctcctcgcccttgctcaccattccgccgcccatgtacaaactaacatctaatctcttggt |
| CYP706X1(F6H)_GFP-5F | TAGCAGCTGTAATACGACTCACTATAGGGAATATTATGGCATCAAACGAGCTTGCTT |
| CYP706X1(F6H)_GFP-3R | aacagctcctcgcccttgctcaccattccgccgccCATGTAAAGGCTTAGGTCACTTAATCT |
| ancXY-16-I109V-F | AACAGAAATCCACCAgTTGCTGGTTTAGCAACATCATAC |
| ancXY-16-I109V-R | GTTGCTAAACCAGCAAcTGGTGGATTTCTGTTTGCGAAAG |
| ancXY-16-G111A-F | GAAATCCACCAATTGCTGcaTTAGCAACATCATACGGTGC |
| ancXY-16-G111A-R | CCGTATGATGTTGCTAAtgCAGCAATTGGTGGATTTCTGT |
| ancXY-16-T114I-F | TGCTGGTTTAGCAAttTCATACGGTGCTAATGATATCG |
| ancXY-16-T114I-R | CATTAGCACCGTATGAaaTTGCTAAACCAGCAATTGGTG |
| ancXY-16-S115T-F | GCTGGTTTAGCAACAaCATACGGTGCTAATGATATCG |
| ancXY-16-S115T-R | CATTAGCACCGTATGtTGTTGCTAAACCAGCAATTGG |
| ancXY-16-A118G-F | CAACATCATACGGTGgTAATGATATCGCTTTCGCAAAC |
| ancXY-16-A118G-R | GAAAGCGATATCATTAcCACCGTATGATGTTGCTAAAC |
| ancXY-16-N119Q-F | ACATCATACGGTGCTcaaGATATCGCTTTCGCAAACAACAAC |
| ancXY-16-N119Q-R | GAAAGCGATATCttgAGCACCGTATGATGTTGCTAA |
| ancXY-16-F123W-F | CTAATGATATCGCTTggGCAAACAACAACTCTAACTGGAG |
| ancXY-16-F123W-R | AGAGTTGTTGTTTGCccAAGCGATATCATTAGCACCGTA |
| ancXY-16-A124S-F | CTAATGATATCGCTTTCtctAACAACAACTCTAACTGGAGA |
| ancXY-16-A124S-R | AGTTAGAGTTGTTGTTagaGAAAGCGATATCATTAGCACCGT |
| ancXY-16-A220L-F | AGATCGTTGAAATCttgGGTGCACCAAACATCTCTGATT |
| ancXY-16-A220L-R | GATGTTTGGTGCACCcaaGATTTCAACGATCTTAGAAACAAC |
| ancXY-16-M248L-F | GAAATGAAAAGACAAtTGAAGCAATTCGATAGAATCTTCG |
| ancXY-16-M248L-R | TATCGAATTGCTTCAaTTGTCTTTTCATTTCTCTTTCAACACC |
| ancXY-16-F251L-F | GACAAATGAAGCAATTgGATAGAATCTTCGAATCAATTATTGAAG |
| ancXY-16-F251L-R | GATTCGAAGATTCTATCcAATTGCTTCATTTGTCTTTTCATTTC |
| ancXY-16-V307L-F | CAAATTAAAGCTTTAttaGTTGATATTTTCTTGGGTGGT |
| ancXY-16-V307L-R | CAAGAAAATATCAACtaaTAAAGCTTTAATTTGTGTGATATTA |
| ancXY-16-F311L-F | CTTTAGTTGTTGATATTTTgTTGGGTGGTACTGATGCTACA |
| ancXY-16-F311L-R | ATCAGTACCACCCAAcAAAATATCAACAACTAAAGCTTTAATTTG |
| ancXY-16-A317T-F | CTTGGGTGGTACTGATaCTACATCTGCAATGGTTGAATG |
| ancXY-16-A317T-R | ACCATTGCAGATGTAGtATCAGTACCACCCAAGAAAATATC |
| ancXY-16-A320T-F | ACTGATGCTACATCTaCAATGGTTGAATGGGCTATG |
| ancXY-16-A320T-R | CCCATTCAACCATTGtAGATGTAGCATCAGTACCAC |
| ancXY-16-L381I-F | CCATTGCCATTGTTAattCCAAGATCTCCAAATCAATCA |
| ancXY-16-L381I-R | GATTTGGAGATCTTGGaatTAACAATGGCAATGGTGGATG |

**Table S4. The strains and plasmids used in this study**

| **Strains** | **Relevant characteristics** | **Source** |
| --- | --- | --- |
| W303-1B | W303 (MATα, ade2–1; ura3–1; his3–11,15; trp1–1; leu2–3,112; can 1–100) | (4) |
| Trans-T1 | F-φ88(lacZ)△M15△lacX74hsdR(rk-,mk+)△recA1398endAltonA | TransGen |
| DMT | F-φ88(lacZ)△M15△(lacZYA-argF)U169△recA1endAhsdR17(rk-,mk+)phoAsupE44thi-1 gyrA96relAl tonA | TransGen |
| BL21(DE3) | F-; ompT; hsdS（rBB-mB－);gal;dcm（DE3） | TransGen |
| **Plasmids** | **Relevant characteristics** | **Reference** |
| YCplac22 | Centromeric vector, TRP1, AmpR | 3 |
| YCplac33 | Centromeric vector, URA3, AmpR | (4) |
| YCplac22-PE | Centromeric vector, TRP1, AmpR, pPGK1 and tCYC1 | (4) |
| YCplac33-PE | Centromeric vector, URA3, AmpR, pPGK1 and tCYC1 | (4) |
| Y33-ATR2 | YCplac33 vector, pTDH3-ATR2-tTDH1 | Lab preserved |
| Y22-ATR2-ancXY | YCplac22 vector, pPGK1-ancXY-tCYC1/pTDH3-ATR2-tTDH1 | This study |
| Y22-ATR2- ancX | YCplac22 vector, pPGK1-ancX-tCYC1/pTDH3-ATR2-tTDH1 | This study |
| Y22-ATR2-ancX1 | YCplac22 vector, pPGK1-ancX1-tCYC1/pTDH3-ATR2-tTDH1 | This study |
| Y22-ATR2-ancX2 | YCplac22 vector, pPGK1-ancX2-tCYC1/pTDH3-ATR2-tTDH1 | This study |
| Y22-ATR2-ancX3 | YCplac22 vector, pPGK1-ancX3-tCYC1/pTDH3-ATR2-tTDH1 | This study |
| Y22-ATR2-ancXY-16 | YCplac22 vector, pPGK1-ancXY-16-tCYC1/pTDH3-ATR2-tTDH1 | This study |
| Y22-Cnan706X | YCplac22 vector, pPGK1-Cnan706X-tCYC1 | Genscript Biotech Corporation |
| Y22-Lasl706X | YCplac22 vector, pPGK1-Lasl706X-tCYC1 | Genscript Biotech Corporation |
| Y22-CYP73A1 | YCplac22 vector, pPGK1-CYP73A1-tCYC1 | Genscript Biotech Corporation |
| Y22-CYP706A3 | YCplac22 vector, pPGK1-CYP706A3-tCYC1 | Genscript Biotech Corporation |
| Y22-CYP706B1 | YCplac22 vector, pPGK1-CYP706B1-tCYC1 | Genscript Biotech Corporation |
| Y22-CYP706C55 | YCplac22 vector, pPGK1-CYP706C55-tCYC1 | Genscript Biotech Corporation |
| Y22-CYP706M1 | YCplac22 vector, pPGK1-CYP706M1-tCYC1 | Genscript Biotech Corporation |
| Y22-Design6444 | YCplac22 vector, pPGK1-Design6444-tCYC1 | Genscript Biotech Corporation |
| Y22-Design11361 | YCplac22 vector, pPGK1-Design11361-tCYC1 | Genscript Biotech Corporation |
| Y22-Design33380 | YCplac22 vector, pPGK1-Design33380-tCYC1 | Genscript Biotech Corporation |
| Y22-Design49566 | YCplac22 vector, pPGK1-Design49566-tCYC1 | Genscript Biotech Corporation |
| Y22-Design58683 | YCplac22 vector, pPGK1-Design58683-tCYC1 | Genscript Biotech Corporation |
| Y22-Design84497 | YCplac22 vector, pPGK1-Design84497-tCYC1 | Genscript Biotech Corporation |
| Y22-Design91808 | YCplac22 vector, pPGK1-Design91808-tCYC1 | Genscript Biotech Corporation |
| Y22-Design26159 | YCplac22 vector, pPGK1-Design26159-tCYC1 | Genscript Biotech Corporation |
| Y22-Design12854 | YCplac22 vector, pPGK1-Design12854-tCYC1 | Genscript Biotech Corporation |
| Y22-Design42565 | YCplac22 vector, pPGK1-Design42565-tCYC1 | Genscript Biotech Corporation |
| Y22-Design33105 | YCplac22 vector, pPGK1-Design33105-tCYC1 | Genscript Biotech Corporation |
| Y22-Design468 | YCplac22 vector, pPGK1-Design468-tCYC1 | Genscript Biotech Corporation |
| Y22-Design4779 | YCplac22 vector, pPGK1-Design4779-tCYC1 | Genscript Biotech Corporation |
| Y22-Design3644 | YCplac22 vector, pPGK1-Design3644-tCYC1 | Genscript Biotech Corporation |
| Y22-Design58 | YCplac22 vector, pPGK1-Design58-tCYC1 | Genscript Biotech Corporation |
| Y22-Design2205 | YCplac22 vector, pPGK1-Design2205-tCYC1 | Genscript Biotech Corporation |
| Y22-Design4129 | YCplac22 vector, pPGK1-Design4129-tCYC1 | Genscript Biotech Corporation |
| pYES2.0_Design11361 | pYES2.0 vector, pGAL1-Design11361-GFP-tCYC1 | This study |
| pYES2.0_Design49566 | pYES2.0 vector, pGAL1-Design49566-GFP-tCYC1 | This study |
| pYES2.0_Design33380 | pYES2.0 vector, pGAL1-Design33380-GFP-tCYC1 | This study |
| pYES2.0-Design58683 | pYES2.0 vector, pGAL1-Design58683-GFP-tCYC1 | This study |
| pYES2.0_Design6444 | pYES2.0 vector, pGAL1-Design6444-GFP-tCYC1 | This study |
| pYES2.0_Design84497 | pYES2.0 vector, pGAL1-Design84497-GFP-tCYC1 | This study |
| pYES2.0_Design91808 | pYES2.0 vector, pGAL1-Design91808-GFP-tCYC1 | This study |
| pYES2.0_CYP706X1(EbF6H) | pYES2.0 vector, pGAL1-EbF6H-GFP-tCYC1 | This study |

## The protein sequences and DNA sequences used in this study

**Protein sequences used in this study**

**Natural sequences**

**>CYP706X1**

MASNELAFSALLVTLVLVLISWYKREISNSRKAGTPPLPPGPKGLPLVGSLPFLGPNIHQELTKISHQYGPIFKLYLGSKLHIVVNSAELAKVITAEQDESFANRAPHIAGLATSYGGNDIAFAPNNANRRNLRKVLVQEVLSNVNLEASHAYRRHEVRKAVKYVYDRVGMDVDINEISFSTVLNVFTNIIWRKGFVDDGANYANLSENIQKVICRIVEIAEGLNISDFFPMLARFDLQGVERKMKDQMKQFDKIIEPTIKERMNSRSTNVEETVEHKGRKDFLQILLEHTDQKNGTSITMTQLKALVADIFLGGTDATSAMVEWAMTEIFRDQKVMKRVQDELEEIVGLNNIVEESHIPKLKYLEAVCKETFRLHPPIPFLLPRAPIKSCTVGGYTVPEGATIFVNVWAIQRDPRHWENPSEFNPDRFLNRNGSTEKWDYSGTNLTFLPFGSGRRRCPGIPLGEKMMMHILASLMHSFDWKLPNGEELDLSERFGIALKKKKPLVAVPTKRLSDLSLYM*

**>Cnan706X**

MAASSHSSLWEEATNNKHEVALAVFVGTLVILAISWYKRSNSRTGKGTPPLPPGPKGLPIVGYLPFLSPNLHHEFAKIANQYGPIFKLYLGSKLHIVVNSADLAKVVTGEQDESFANRDPHIAGLTASYGASDVAWQNNNSNRRNLRKVLVHEVLSNKNLEASHAYRRSEVRKTIKNVHDMIGTAVDINEVSFSTVLNILTHIVWGNSFVEGAKYPNLAADIRKVVLDIVEIAEGLNLSDFFPMLARFDFQGVERRVKAQVKKFDHIFETTIEERTNSKSKVSEEAVKQEGRKDFLQILLELLDQNTATSINMTQLKALVVDIFLGGTDATAAMTEWAMAEILRNPKVMKKVQDELAEVVGLNNIVEESHLPKLKYLDAVFKETFRLHTPLPFLLPRTPDKSCVVGGYTVPKGATVFLNVWAIQRDPQNWENPSEFNPERFLNNKGSEKWDYSGTNSTYFPFGSGRRRCPGILLGEKMMMHILASLMHSFDWSLPKGEELDLSDKFGIAMKKKMPLVVIPSLRLSDLSLYS*

**>Lsal706X**

MASNELAFSALLVTLVLVLISWYKREISNSRKAGTPPLPPGPYGLPLVGYLPFLGPSLHHELTKMAHRYGPIFKLYLGSKLHIVVNSADLAKVITSEQDESFANRAPHIAGLATSYGANDIAFADNNANRRNLRKILVHEILSNVNLEASHAYRRREVRKTIKSVHDMIGMPVDINEMSFSTVVNVLTSIVWGNSMVEGTKHSNLGEEIRKVVSEIVDIAEGLNISDFFPKLARFDLQGVEQKMKRKMKQFDWIFETTIEERINLKSTHGEDALKHEGRKDFLQILLELKDKKSITMTQLKALVVDIFLGGTDATSAMVEWAMAEILKNQKVMKKVQDELAEIVGLKNMVEESHLPKLKYLNATFKETFRLHTPLPVLLPRTPSKSCMVGGYLIPRDSTVFLNVWAIQRDPQHWENPSEFNPERFLNYEGSGKWDYSGTNSKYFPFGSGRRRCPGIPLAEKMMLHILASLLHSFDWSLPKGEDHDLFEKFGIALKKKKPLVAVPSPRLIDLSLYM*

**Ancestral protein sequences**

**>ancXY** MASNELAFSALLVTLVLVLISWYKREISNSRKAGTPPLPPGPRGLPVVGYLPFLGPNLHQEFTKMAHRYGPIFKLHLGSKLHIVVNSADLAKVVAREQDETFANRNPPVAALAITYGGQDIAWSNNNSNWRNLRKVLVHEVLSNKNLEASRSFRRREVRKTIKNVYEKIGTEIDINEIAFSTELNVLTSMVWGKSLVEGEKYSNLGDEFREVVSKIVEILGAPNISDFFPILAWFDLQGVEREMKRQLKQLDRIFESIIEERINSNSTKSEEAVEHEGRKDFLQILLELKDQKDATSINITQIKALLVDILLGGTDTTSTMVEWAMAEILQNQKVMKKVQDELAEIVGLNNIVEESHLPKLKYLDAVIKETFRLHPPLPLLIPRSPNQSCTVGGYTIPKGSTVFLNVWAIHRDPQYWDNPLEFNPERFLNREGTDKWDYNGNNLKFLPFGSGRRRCPGIPLGEKMLMYILASLLHSFDWSLPKGEEHDLSDKFGIALKKRKPLIAIPSQRLPDASLYM*

**>ancX**

MASNELAFSALLVTLVLVLISWYKREISNSRKAGTPPLPPGPRGLPLVGYLPFLGPNLHQELTKMAHRYGPIFKLYLGSKLHIVVNSADLAKVVTGEQDESFANRAPHIAGLATSYGANDIAFADNNANRRNLRKVLVHEVLSNVNLEASHAYRRREVRKTIKNVHEMIGNEVDINEIAFSTVLNVLTSIVWGKSMVEGAKYSNLGEEIRKVVSGIVEIAGGLNISDFFPMLARFDLQGVERKMKRQMKQFDKIFESTIEERINSKSTNVEEAVKHEGRKDFLQILLELKDQKNETSITMTQLKALVVDIFLGGTDATSAMVEWAMTEILRNQKVMKKVQDELAEIVGLNNIVEESHLPKLKYLDAVFKETFRLHPPLPFLLPRAPNKSCTVGGYTIPKGSTIFLNVWAIQRDPQYWENPSEFNPERFLNYEGSEKWDYSGTNSKFFPFGSGRRRCPGIPLGEKMMMHILASLLHSFDWSLPKGEEHDLSDKFGIALKKRKPLIAVPSPRLSDASLYM*

**>ancX1**

MASNELAFSALLVTLVLVLISWYKREISNSRKAGTPPLPPGPKGLPLVGSLPFLGPNIHQELTKISHQYGPIFKLYLGSKLHIVVNSAELAKVITAEQDESFANRAPHIAGLATSYGGNDIAFAPNNANRRNLRKVLVQEVLSNVNLEASHAYRRHEVRKAVKYVYDRVGMDVDINEISFSTVLNVLTNIIWRKGFVDDGANYANLSEKIQKVICRIVEIAEGLNISDFFPMLARFDLQGVERKMKDQMKQFDKIIETTIKERMNSKSTNVEETVEHKGRKDFLQILLEHTDQKNGTSITMTQLKALVADIFLGGTDATSAMVEWAMTEIFRDQKVMKRVQDELEEIVGLNNIVEESHIPKLKYLEAVCKETFRLHPPIPFLLPRAPNKSCTVGGYTVPEGATIFVNVWAIQRDPRHWENPSEFNPDRFLNCNGSTEKWDYSGTNLTFLPFGSGRRRCPGIPLGEKMMMHILASLMHSFDWKLPNGEELDLSERFGIALKKKKPLVAVPTKRLSDLSLYM*

**>ancX2**

MASNELAFSALLVTLVLVLISWYKREISNSRKAGTPPLPPGPRGLPLVGYLPFLGPNLHQELTKIAHRYGPIFKLYLGSKLHIVVNSADLAKVITGEQDESFANRAPHIAGLATSYGANDIAFADNNANRRNLRKVLVHEVLSNVNLEASHAYRRREVRKTIKNVHDMIGMEVDINEISFSTVLNVLTSIVWGKGMVEGAKYSNLGEEIRKVVSGIVEIAEGLNISDFFPMLARFDLQGVERKMKRQMKQFDKIFETTIEERINSKSTNVEEAVKHEGRKDFLQILLELKDQKNGTSITMTQLKALVVDIFLGGTDATSAMVEWAMTEILRNQKVMKKVQDELAEIVGLNNIVEESHLPKLKYLDAVFKETFRLHPPLPFLLPRTPNKSCTVGGYTIPKGSTIFLNVWAIQRDPQHWENPSEFNPERFLNYEGSEKWDYSGTNSKFFPFGSGRRRCPGIPLGEKMMMHILASLLHSFDWSLPKGEEHDLSEKFGIALKKKKPLVAVPSPRLSDLSLYM*

**>ancX3**

MASNELAFSALLVTLVLVLISWYKREISNSRKAGTPPLPPGPRGLPLVGYLPFLGPNLHQELTKMAHRYGPIFKLYLGSKLHIVVNSADLAKVVTGEQDESFANRAPHIAGLATSYNASDIAFADNNANRRKLRKVLVHEVLSNVNLEASHAYRRREVRKTIKNVHEIIGNEVDINEIAFSTVLSVLTSIVWGKSMVKGAKYSNLVAEMRKFVSGVVEIAGELNISDFFPMLARFDFQGVERRMKKQMKLFDKIFESTVEERINSRSAIKEEAVKEEGRKDFLQILLELQEQNNETSITMTQMKALVVDIFLGGTDATSAMIEWAMTEILRNRQVMKKVQDELAEIVGLNNIVEESHLPKLKYLDAVFKETFRLHPPLPFLLPRAPNKSCTVGGYTIPKGSTIFLNVWAIQRDPQYWENPSEFNPERFLNYKGSEKWDYAGTNSKFFPLGSGRRRCPGVSLGEKMMMHILASLLHSFDWSLPTGQKLDLSDKFGIALKKRKPLIAVPSPRLNDASLYM*

**>ancX-16**

MASNELAFSALLVTLVLVLISWYKREISNSRKAGTPPLPPGPRGLPVVGYLPFLGPNLHQEFTKMAHRYGPIFKLHLGSKLHIVVNSADLAKVVAREQDETFANRNPPIAGLATSYGANDIAFANNNSNWRNLRKVLVHEVLSNKNLEASRSFRRREVRKTIKNVYEKIGTEIDINEIAFSTELNVLTSMVWGKSLVEGEKYSNLGDEFREVVSKIVEIAGAPNISDFFPILAWFDLQGVEREMKRQMKQFDRIFESIIEERINSNSTKSEEAVEHEGRKDFLQILLELKDQKDATSINITQIKALVVDIFLGGTDATSAMVEWAMAEILQNQKVMKKVQDELAEIVGLNNIVEESHLPKLKYLDAVIKETFRLHPPLPLLLPRSPNQSCTVGGYTIPKGSTVFLNVWAIHRDPQYWDNPLEFNPERFLNREGTDKWDYNGNNLKFLPFGSGRRRCPGIPLGEKMLMYILASLLHSFDWSLPKGEEHDLSDKFGIALKKRKPLIAIPSQRLPDASLYM*

**Other subfamily protein sequences**

>CYP73A1 (NCBI Number: Q04468)

MDLLLIEKTLVALFAAIIGAILISKLRGKKFKLPPGPIPVPIFGNWLQVGDDLNHRNLTDLAKRFGEILLLRMGQRNLVVVSSPELAKEVLHTQGVEFGSRTRNVVFDIFTGKGQDMVFTVYGEHWRKMRRIMTVPFFTNKVVQQYRYGWEAEAAAVVDDVKKNPAAATEGIVIRRRLQLMMYNNMFRIMFDRRFESEDDPLFLKLKALNGERSRLAQSFEYNYGDFIPILRPFLRNYLKLCKEVKDKRIQLFKDYFVDERKKIGSTKKMDNNQLKCAIDHILEAKEKGEINEDNVLYIVENINVAAIETTLWSIEWGIAELVNHPEIQAKLRHELDTKLGPGVQITEPDVQNLPYLQAVVKETLRLRMAIPLLVPHMNLHDAKLGGFDIPAESKILVNAWWLANNPDQWKKPEEFRPERFLEEEAKVEANGNDFRYLPFGVGRRSCPGIILALPILGITIGRLVQNFELLPPPGQSKIDTDEKGGQFSLHILKHSTIVAKPRSF*

>CYP706A3 (NCBI Number: KAG7604930)

MTDISSLFRNRSRKDQLDYGLTVIVISTLCWCLWLYAKCKRRSPPLPPGPWGLPIIGNLPFLQPELHTYFQGLAKKHGPIFKLWLGAKLTIVVTSSEVAQEILKTNDIIFANHDVPAVGLVNTYGGTEIIWSPYGPKWRMLRKLCVNRILRNAMLDSSTDLRRRETRQTVRYLADQARVGSPVNLGEQIFLMMLNVVTQMLWGTTVKEEEREVVGAEFLEVIREMNDLLLVPNISDFFPVLSRFDLQGLAKRMRRPAQRMDQMFDRIINQRLGMDRDSSDGRAVDFLDVLLKVKDEEAEKTKLTMNDVKAVLMDMVLGGTDTSLHVIEFAMAELLHNPDIMKRAQQEVDKVVGKEKVVEESHISKLPYILAIMKETLRLHTVAPLLVPRRPSQTTVVGGFTIPKDSKIFINAWAIHRNPNVWENPLKFDPDRFLDMSYDFKGNDFNYLPFGSGRRICVGMAMGERVVLYNLATFLHSFDWKIPQGERVEVEEKFGIVLELKNPLVATPVLRLSDPNLYL*

>CYP706B1 (NCBI Number: XP_016705189)

MLQIAFSSYSWLLTASHQKDGMLFPVALSFLVAILGISLWHVWTIRKPKKDIAPLPPGPRGLPIVGYLPYLGTDNLHLVFTDLAAAYGPIYKLWLGNKLCVVISSAPLAKEVVRDNDITFSERDPPVCAKIITFGLNDIVFDSYSSPDWRMKRKVLVREMLSHSSIKACYGLRREQVLKGVQNVAQSAGKPIDFGETAFLTSINAMMSMLWGGKQGGEQKGADVWGQFRDLITELMVILGKPNVSDIFPVLARFDIQGLEKEMTKIVNSFDKLFNSMIEERENFSNKLSKEDGNTEAKDFLQLLLELKQKNDSGISITMNQVKALLMDIVVGGTDTTSTMMEWTMAELIANPEAMKKVKQEIDDVVGSDAAVDETHLPKLRYLDAAVKETFRLHPPMPLLVPRCPGDLSNVGGYSVPKGTRVFLNIWCIQRDPQLWENPLEFKPERFLTDHQKLDYLGNDSRYMPFGSGRRMCAGVSLGEKMLYSSLAAMIHAYDWNLADGEENDLIGLFGIIMKKKKPLILVPTPRPSNLQHYMK*

>CYP706C55 (NCBI Number: AYN73068)

MSPSISLSLLHNLESSLSSNDFSLLSLLFLLSGALAIGFWAWRRSAEKKNSLPLPPGPAGLPLVGNLPFLDPELHTYFATLAMTYGPILKLQLGKKLGIVVTSPATAREVLKDNDVTFANRDVPIAGRVAFYGGSDIVWNSYGPEWRMFRKVCGLKMLSNHALDSVYELRRREVRRTVGYFLLQAGSPVNVGEQMFLTVLNVITSMLWGGTAQGEEKESLGADFRQAVSSLTKLLGKPNISDFYPSLARFDLQGIERQMKGLAKRFDGIFQKMIEQRLKMQRENGSESLDGGEENKDFLQFLLNLKDEEDAQTQLTTTGLKALLMDMLVGGTDSSSNTIEFAMAEIINKPKVLQNIQQELETVVGRGNVVEESHIPKLPYLQAVMKESLRLHPPVPLLIPHCPSATCTVGGYTVPKGSRVFINVWAIHRDPLIWRDPLEFDPERFLHSEGNYNEHNFNYFPFGSGRRMCVGILMAERMVLYSLATLLHSFDWKLPKGEKMDLTEQFGIVMKKKKPLMAVPSPRFSNSRLYE*

>CYP706M1 (NCBI Number: AGJ03150)

MDMSTIWYYWVSIILGVFIFLIVGIQKWRSKKLPPGPFALPLLGHLHLLEPNVHECLSKISEKFGPLMSFKFGMKTSIIVSSPAMAKEILRENDQIFANRSIPVVARCIAYDASDILWSPNGPRWRLLRKICVKELFSPKSTEALQPLRREEVRRTMGNIYKDSINGVSVDVGAKAFITSLNLITNMMWSTSTETGERGGEFKDLVGELVHVLGVPNASDLFPFLERFDVQGLYRRMEKVFVRFDKMFDGIIEDKLSGKSKEKDFLQSLLDLVERGVDEQDPDSVQLTMKDVKVLLMDMVTGSTDTTSNTVEWAMAELLQQPEIMKRAQKELEEVVGLDNMVEECHLSQLPYLDIIVKEVLRLHPALPLLAPHRPERECEIGGYIIPKDTQVLINVWSIQRNPKVWKEPLLFDPERFSDSKWDYNGRDFDYFPFGSGRRICAGLSMAKIMVHYSLASLLHSFDWSLPVAEKLNMDEKYGIVLRKAVPLVALPKPRLLYPNLYE*

**Deep learning protein sequences**

**>Design11361**

MASNELAFSALLVTLVLVLISWYKREISNSRKAGTPPLPPGPKGLPLVGFLPFLGPNLHLDLLKMANNYGPIFKLYLGSNLHIVVNSADLAKVVTGEQDESFANRAQHIAGLATSYNASDIAFADNNANRRKLRKVLVHEVLSNVNLEASNAYRRREVRKTIKNVHEIIGNEVDINEIAFSTVLSVLTSIVFGKSMVKGAKYSNLVADMRKFVSGVVEIAGGLNISDFFPVLARFDFQGVKRKMADQMKMFDKIFETSVEERINSRSAIIEETVKQEGRKDFLQILLELLDQNTETSITMTQLKALVVDIFLGGTDATSAMVEWAMTEIFRDKKVMKRVQDELAEVVGLNNIVEESHLPKLKYLDAVFKETFRLHPPLPFLLPRAPNKSCTVGGYTVPKGSTIFLNVWAIQRDPQYWENPSDFNPERFLNYKGSNKWDYAGTNLKFFPFGSGRRRCPGVSLGEKMLMHILASLLHSFDWSLPTGQKLDLSDKFGITLKKRKPLIAVPSPRLSDASLYL*

**>Design33380**

MASNELAFSALLVTLVLVLISWYKREISNSRKAGTPPLPPSPKSLPIVGHLPFLGTDIHHELTEISHQYGPIFKFHLGSKLHIIINSAELAKVITVEQDESFANRWPHIAGIATSYGGNDIAFAPNNANWRNLRKVLVQEVLSNVNLEASHAYRRHEVRKAVKYVYDRVGMDVDINEISFSTVLNVFSNIIWRKGFVDDGTNYANLSEKIQKVICRIVEIAEGLNISDFFPMLARFDLQGVERKMKTQMKQFDKIFETTVDERINSKPAISEEAVKEEGRKDFLQILLELLDQNTATSITMTQMKALVVDVFLGGTDATSAMTEWAMTEILRNRQVMKKVQDELAEVVGLNNIVEESHLPKLKYLDAVFKETFRLHPPLPFLLPRAPNKSCTVGGYTVPKGSTIFLNVWAIQRDPQHWTNPSEFNPERFLNKGSEKWDYNGTNSKYFPFGSGRRRCPGIPLGEKMMMHILASLMHSFDWSLPRGEEHDLSDKFGIAMKKKMPLVLIPSQRLSDHNLYM*

**>Design58683**

MASNELAFSALLVTLVLVLISWYKREISNSRKAGTPPLPPGPKGLPVVGFLPFLGPNLHLDFLTLVHKYGPIFKLYLGSNLHIVVNSADLAKVVTGEQDESFANRAQHIAGLATSYNASDIAFADNNANRRKLRKVLVHEVLSNVNLEASNAFRRREVRKTIKNVHEIIGNEVDINEIAFSTVLSVLTSIVFGKSMVKGAKYSNLVAEMRKFVSGVVEIAGELNISDFFPMLARFDFQGVKRRMAQQMKIFDRIFETTIEERTGSTSGIIDQKVKEEGRKDFLQILLELLDQNTGTSITMTQLKALVVDIFLGGTDATSAMVEWAMTEIFRDKKVMKRVQDELAEVVGLHNIVEESHLPKLKYLDAVFKETFRLHPPLPFLLPRAPNKSCTVGGYTVPKGSTIFLNVWAIQRDPRYWENPSDFNPERFLNYKGSEKWDYSGTNLKFFPFGSGRRRCPGVPLGEKMMMHILASLLHSFDWSLPTGEKLDLSDKFGITLKKRKPLIAIPSMRLSDASLYM*

**>Design6444**

MASNELAFSALLVTLVLVLISWYKREISNSRKAGTPPLPPGPKGLPLVGFLPFLGPNLHLDFLTMAHQYGPIFKLYLGSNLHIVVNSADLAKVVTGEQDESFANRAQHIAGLATSYNASDILFADNNANRRKLRKVLVHEVLSNVNLEASNAYRRREVRKTIKNVHEIIGNEVDINEIAFSTVLSVLTSIVFGKSMVKGAKYSNLVADMRKFVSGVVEIAGGLNISDFFPMLARFDFQGVKRKMAQQMKMFDKIFESTVEERVGSTSGIIEEAVKQEGRKDFLQILLELLDQNTETSITMTQMKALVVDIFLGGTDATSAMVEWAMTEIFRDKKVMKRVQDELAEVVGLHNIVEESHLPKLKYLDAVFKETFRLHPPLPFLLPRAPNKSCTVGGYTVPKGSTIFLNVWAIQRDPQYWENPSEFNPERFLNYKGSEKWDYTGTNLKFFPFGSGRRRCPGVPLGEKMMMHILASLLHSFDWSLPNGQKLDLSDKFGITLKKRKPLIAVPSLRLSDASLYV*

**>Design84497**

MASNELAFSALLVTLVLVLISWYKREISNSRKAGTPPLPPGPRGLPLVGYLPFLGPQPHRSLSEISHRYGPIFKLQLGTKLWIVVNSAELAKVIHVEQDESFANRAPHIAGLATSYGGNDIAFAPNNANRRNLRKLLVQEVLSNVNLEASHAYRRHEVRKAVKYVYDRVGMDIDINEISFTTVINVFFNIWRMGFIDDQSNIGNLLEKIQKVICRIVEIAEGLNISDFFPVLARFDLQRVERKMKDQMKQFDKIIETTIKERMNSKSTNVEETVEQEGRKDFLQILLELLDQSTATSITMTQLKALVVDVFLGGTDATSAMVEWAMTEILRNRQVMKKVQDELAQVVGLHNVVEESHLPKLKYLDAVFKETFRLHPPLPFLLPRAPNKSCTVGGYTVPKGSTIFLNVWAIQRDPQHWTNPSEFNPERFLNYKGSEKWDYAGTNSKFFPLGSGRRRCPGIPLGEKMMMHILASLLHSFDWSLPTGQKLDLSDKFGIAMKKKKPLVVVPSLRLSDLSLYS*

**>Design91808**

MASNELAFSALLVTLVLVLISWYKREISNSRKAGTPPLPPGPKGLPIVGYLLFLGTNLHIHFSNLSQSYGPIFKFHLGNKLWVIVNTAELAKTIVVEQDESFANRWPHIAGLATSYGGNDIAFAPNNANRRNLRKVLVQEVLSNVNLEASHNYRRHEVRKAVKYVYDRVGMDIDINEISFTTVLNVFFNIIWRMGFEDDQTNVGNLLQKIQKVICRIVEIAEGLNISDFFPVLARFDVQRVERKMKDQMKQFDKIIETTIKERMNSKSTNVEETVEQKGRKDFLQILLELLDQNNETSITMTQMKALVVDIFLGGTDATSAMVEWAMTEILRNRQVMKKVQDELAEIVGLNNIVEESHLPKLKYLDAVFKETFRLHPPLPFLLPRAPNKTCTVGGYTVPKGSTIFLNVWAIQRDPQYWDNPSEFNPERFLNYKGSEKWDYNGTNLKFFPFGSGRRRCPGIPLGEKMMMHILASLMHSFDWSLPRGEELDLSDKFGIAMKKKKPLVVIPSLRLSDHNLYM*

**>Design49566**

MASNELAFSALLVTLVLVLISWYKREISNSRKAGTPPPLPPGPKGLPLVGSLPFLGPNIHQELTKITHQYGPIFKLYLGSKLHIVVNSAELAKVITAEQDESFANRAPHIAGLATSYGGNDIAFAPNNANRRNLRKVLVQEVLSNVNLEASHAYRRHEVRKAVKYVHERVGMEVDINKIAFSTVLSVLTNIVWAKSVVDDGANYSNEVQNIISRVVEIAGGLNISFFVMAQIDFQGVERRMKKQMKQFDKIIEMTIEERMNSSKNEAKTKEEGRKDFLQILLEQQQQNTETSITMTQMKALVVDIFLGGTDATSAMVEWAMTEILRDQQVMKRVQDELEEIVGLNNVEESHLPKLKYLDAVFKETFRLHPPIPFLLPRAPIKSCTVGGYTVPEGATIFVNVWAIQRDPQRWENPSEFNPDRFLNRNGSTGKWDYSGTNLTFLPFGSGRRRCPGIPLGEKMMMHILASLLHSFDWKLPNGQKLELSERFGIALKKKKPLVAVPTKRLDVSLYM*

**>Design12854**

MAALNTHGSWWPAEGNGGKNDGDLPLALLAVITAALLPLLWYKRSISSSQNGAPPLPPGPKGLPVVGYLPFLGPNLHLDYLTMVHQYGPIFKIYLGSNLHIVVNSVDLAKVVTGEQDESFANRAQHIAGLATSYNASDIAFADNNANRRKLRKVLVHEVLSNVNLEASNAYRRREVRKTIKNVHEVIGNEVDINEISFSTVLSVLTSIVFGKSMVKGAKYSNLAADIRKFVSGVVEIAGGLNISDFFPMLARFDFQGVEQRMKTQMKMFDKIFETSVEERINSRSAIKEEAVKEEGRKDFLQILLELLEQNTETSITMTQMKALVVDVFLGGTDATSAMVEWAMTEIFRNRQVMKKVQDELAEIVGLHNIVEESHLPKLKYLDAVFKETFRLHPPLPFLLPRAPNKTCTVGGYTVPKGSTIFLNVWAIQRDPKYWDNPSDFNPERFLNYEGEKWDYNGTNLKFFPFGSGRRRCPGIPLGEKMMMHILASLLHSFNWSLPEGEDHDLSEKFGIAMKKKKPLIAIPSLRLSDHNLYM*

**>Design33105**

MASNELAFSALLVTLVLVLISWYKREISNSRKAGTPPLPPGPRGLPLVGYLPFLGPNLHQELTKMAHRYGPIFKLYLGSKLHIVVNSADLAKVVTGEQDESFANRAPHIAGLATSYNASDIAFADNNANRRKLRKVLVHEVLSNVNLEASHAYRRREVRKTIKNVHEIIGNEVDINEIAFSTVLSVLTSIVWGKSMVKGASNMIVEVRKFVSGVVEIAGELNISDFFPMLARFDFQGVERRMKKQMKLFDKIFESTVEERINSRSIIKEEAASKEENRRKDFLQILLELQEQNNTSITMTQMKALVVDIFLGGTDATSAMIEWAMTEILRNRRVMKKVQDELAEIVGLQNNVEEESHLPKLKYLDAVFKETFRLHPPLPFLLPRAPNKSCTVGGYTIPKGSTIFLNVWAIQRDPQYWENPSEFNPERFLNYKGSEKWDYAGTNSKFFPLGSGRRRCPGVSLGEKMMMHILASLLHSFDWSLPTGQKLDLSDKFGIALKKRKPLIAVPSPRLNDASLYM*

**>Design42565**

MASNELAFSALLVTLVLVLISWYKREISNSRKAGTPPLPPGPKGLPLVGYLPFLGPNLHHEFTKVSHRYGPIFKLYLGSKLHIVVNSADLAKVITSEQDESFANRAPHIAGLATSYGGNDIAFADNNANRRNLRKVLVHEVLSNVNLEASHAYRRHEVRKTIKSVHDMIGMEVDINEISFSTVLNVLTNIVWGKGLVEGTKYSNLSEEIRKVVYRIVEIAEGLNISDFFPMLARFDLQGVERKMKKQMKQFDRIFENTISERTNNKNSNHGKQCLQILLELKTKTITTTQLKALVVDIFLGGTDATSAMVEWAMTEILRNKKVMKRVQDELEEIVGLNNIVEESHIPKLKYLDAVFKETFRLHPPLPFLLPRAPSKSCTVGGYTVPKGATIFLNVWAIQRDPRHWENPSEFNPDRFLNNNNGSTEKWDYSGTNLTFLPFGSGRRRCPGIPLGEKMMMHILASLMHSFDWSLPNGEEHDLSDKFGIALKKKKPLVAIPTRRLSDENLYM*

**>Design26159**

MASNELAFSALLVTLVLVLISWYKREISNSRKAGTPPLPPGPYGLPLLGYLPFFLGPSLHHELTKMAHRYGPIFKLYLGSKLHIVVNSADLAKVITSEQDESFANRAPHIAGLATSYGANDIAFADNNANRRNLRKILVHEILSNVNLEASHAYRRREVRKTIKSVHDMIGMPVDINEMSFSTVVNVLTSIVWGNSMVEGTKHSNLGEEIRKVVSEIVDIAEGLNISDFFPKLARFDLQGVEQKMKRKMKQFDWIFETTIEERINLKSTHGEDALKHEGRKDFLQILLELKDKKSITMTQLKALVVDIFLGGTDATSAMVEWAMAEILKNQKVMKKVQDELAEIVGLKNMVEESHLPKLKYLNATFKETFRLHTPLPVLLPRTPSKSCMVGGYLIPRDSTVFLNVWAIQRDPQHWENPSEFNPERFLNYEGSGKWDYSGTNSKYFPFGSGRRRCPGIPLAEKMMLHILASLLHSFDWSLPKGEDHDLFEKFGIALKKKKPLVAVPSPRLIDLSLYM*

**>Design49566**

MASNELAFSALLVTLVLVLISWYKREISNSRKAGTPPPLPPGPKGLPLVGSLPFLGPNIHQELTKITHQYGPIFKLYLGSKLHIVVNSAELAKVITAEQDESFANRAPHIAGLATSYGGNDIAFAPNNANRRNLRKVLVQEVLSNVNLEASHAYRRHEVRKAVKYVHERVGMEVDINKIAFSTVLSVLTNIVWAKSVVDDGANYSNEVQNIISRVVEIAGGLNISFFVMAQIDFQGVERRMKKQMKQFDKIIEMTIEERMNSSKNEAKTKEEGRKDFLQILLEQQQQNTETSITMTQMKALVVDIFLGGTDATSAMVEWAMTEILRDQQVMKRVQDELEEIVGLNNVEESHLPKLKYLDAVFKETFRLHPPIPFLLPRAPIKSCTVGGYTVPEGATIFVNVWAIQRDPQRWENPSEFNPDRFLNRNGSTGKWDYSGTNLTFLPFGSGRRRCPGIPLGEKMMMHILASLLHSFDWKLPNGQKLELSERFGIALKKKKPLVAVPTKRLDVSLYM*

**>****Design58**

MASNELAFSALLVTLVLVLISWYKREISNSRKAGTPPLPPGPKGLPLVGYLPFLGPNLHQELTKMAHRYGPIFKLYLGSKLHIVVNSADLAKVVTGEQDESFANRAPHIAGLATSYNASDIAFADNNANRRKLRKVLVHEVLSNVNLEASHAYRRREVRKTIKNVHEIIGNEVDINEIAFSTVLSVLTSIVWGKSMVEGAKYSNLVAEMRKFVSGVVEIAGELNISDFFPMLARFDFQGVERRMKKQMKLFDKIFESTVEERINSRKSVEKEDFLQILLELQEQNNETSITMTQMKALVVDIFLGGTDATSAMIEWAMTEILRNRQVMKKVQDELAEIVGLNNIVEESHLPKLKYLDAVFKETFRLHPPLPFLLPRAPNKSCTVGGYTIPKGSTIFLNVWAIQRDPQYWENPSEFNPERFLNYKGSEKWDYAGTNSKFFPLGSGRRRCPGVSLGEKMMMHILASLLHSFDWSLPTGQKLDLSDKFGIALKKRKPLIAVPSPRLNDASLYM*

**>** **Design4779**

MASNELAFSALLVTLVLVLISWYKREISNSRKAGTPPLPPGPRGLPLVGYLPFLGPNLHQELTKMAHRYGPIFKLYLGSKLHIVVNSADLAKVVTGEQDESFANRAPHIAGLATSYNASDIAFADNNANRRKLRKVLVHEVLSNVNLEASHAYRRREVRKTIKNVHEIIGNEVDINEIAFSTVLSILTSIVWGKSMVEGAKYTNLVAEMRKFVSGVVEIAGELNISDFFPMLARFDFQGVERRMKKQMKLFDKIFESTVEERINSSKKEGRKDFLQILLELQEQNNETSITMTQMKALVVDIFLGGTDATSAMVEWAMTEILRNRQVMKKVQDELAEIVGLNNIVEESHLPKLKYLDAVFKETFRLHPPLPFLLPRAPNKSCTVGGYTIPKGSTIFLNVWAIQRDPQYWENPSEFNPERFLNYKGSEKWDYAGTNSKFFPLGSGRRRCPGVSLGEKMMMHILASLLHSFDWSLPTGQKLDLSDKFGIALKKRKPLIAVPSPRLNDASLYM*

**>Design3644**

MASNELAFSALLVTLVLVLISWYKREISNSRKAGTPPLPPGPRGLPLVGYLPFLGPNLHHELTKMAHRYGPIFKLYLGSKLHIVVNSADLAKVVTGEQDESFANRDPHIAGLATSYNANDIAFADNNANRRKLRKVLVHEVLSNVNLEASHAYRRREVRKTIKNVHEIIGNEVDINEIAFSTVLSVLTSIVWGKSMVKGAKYPNLVAEMRKFVSGVVEIAGELNISDFFPMLARFDFQGVERRMKKQMKLFDKIFESTVEERINSRKKIARKDFLQILLELKEQNNETSITMTQMKALVVDIFLGGTDATSAMIEWAMTEILRNRQVMKKVQDELAEIVGLNNIVEESHLPKLKYLDAVFKETFRLHPPLPFLLPRAPNKSCTVGGYTIPKGSTIFLNVWAIQRDPQYWENPSEFNPERFLNYEGSEKWDYAGTNSKFFPLGSGRRRCPGIPLGEKMMMHILASLMHSFDWSLPEGEKLDLSDKFGIALKKKKPLIAVPSPRLNDASLYM*

**>Design2205**

MASNELAFSALLVTLVLVLISWYKREISNSRKAGTPPLPPGPRGLPLVGYLPFLGPNLHHELDKMAHRYGPIFKLYLGSKLHIVVNSADLAKVVTGEQDESFANRAPHIAGLATSYNASDIAFADNNANRRKLRKVLVHEVLSNVNLEASHAYRRREVRKTIKNVHEIIGNEVDINEIAFSTVLSVLTSIVWGKSMVKGAKYGNLVAEMRKFVSGVVEIAGELNISDFFPMLARFDFQGVERRMKKQMKLFDKIFETTVEERTNSSKKKVKKDFLQVLLELKEQNNETSINMTQMKALVVDIFLGGTDATSAMIEWAMTEILRNKQVMKKVQDELAEVVGLNNIVEESHLPKLKYLDAVFKETFRLHPPLPFLLPRAPNKSCTVGGYTIPKGSTIFLNVWAIQRDPQYWENPSEFNPERFLNYKGSEKWDYAGTNSKFFPLGSGRRRCPGVSLGEKMMMHILASLLHSFDWSLPEGQKLDLSDKFGIALKKKKPLIAIPSPRLNDASLYM*

**>****Design4129**

MASNELAFSALLVTLVLVLISWYKREISNSRKAGTPPLPPGPRGLPLVGYLPFLGPNLHQELTKMAHRYGPIFKLYLGSKLHIVVNSADLAKVVTGEQDESFANRAPHIAGLATSYNASDIAFADNNANRRKLRKVLVHEVLSNVNLEASHAYRRREVRKTIKNVHEIIGNEVDINEIAFSTVLSVLTSIVLKGSNIAAEMRKFVSGVVEIAGELNISDFFPMLARFDFQGVERRMKKQMKLFDKIFESTVEERINSRSAIKEEAVKEEGRKDFLQILLELQEQNNETSITMTQMKALVVDIFLGGTDATSAMIEWAMTEILRNRQVMKKVQDELAEIVGLNNIVEESHLPKLKYLDAVFKETFRLHPPLPFLLPRAPNKSCTVGGYTIPKGSTIFLNVWAIQRDPQYWENPSEFNPERFLNYKGSEKWDYAGTNSKFFPLGSGRRRCPGVSLGEKMMMHILASLLHSFDWSLPTGQKLDLSDKFGIALKKRKPLIAVPSPRLNDASLYM*

**DNA sequences used in this study**

**Natural sequences**

**>CYP706X1**

ATGGCATCAAACGAGCTTGCTTTTTCAGCACTATTAGTTACACTTGTGTTAGTTCTTATTTCATGGTACAAAAGAGAAATCTCCAACTCCCGAAAGGCAGGCACACCTCCATTGCCTCCGGGTCCAAAAGGTCTACCATTAGTTGGATCTCTTCCATTTCTTGGCCCTAATATTCACCAAGAACTAACCAAAATATCGCACCAATATGGCCCGATTTTTAAGCTATACCTTGGAAGCAAGCTTCACATTGTGGTGAACTCTGCTGAACTGGCAAAGGTCATAACCGCTGAGCAAGACGAGAGCTTTGCTAACCGGGCCCCACATATTGCCGGGCTAGCAACAAGTTACGGTGGCAACGATATAGCATTTGCACCAAATAATGCTAACCGACGTAACCTACGTAAAGTTTTGGTCCAAGAGGTCTTAAGTAATGTCAACCTTGAGGCGTCTCATGCGTATCGTAGACATGAGGTTAGAAAGGCCGTTAAATATGTCTACGATAGGGTTGGTATGGACGTTGATATCAACGAGATATCGTTCTCAACGGTGTTGAATGTGTTTACAAACATAATATGGAGGAAAGGGTTTGTGGATGATGGGGCAAATTATGCTAATCTTAGTGAGAATATACAAAAAGTGATATGTAGAATTGTTGAGATCGCGGAAGGGCTAAATATCTCGGACTTCTTCCCAATGCTTGCAAGGTTTGATCTTCAAGGAGTTGAACGAAAAATGAAGGATCAAATGAAGCAATTCGACAAGATTATAGAGCCTACTATCAAGGAGAGAATGAACTCGAGGTCTACAAACGTTGAAGAAACCGTTGAGCATAAAGGAAGGAAAGATTTTCTACAAATATTGTTAGAGCATACAGATCAAAAAAATGGGACATCAATCACCATGACTCAATTAAAAGCGCTTGTTGCGGATATATTTCTAGGAGGAACAGATGCAACTTCAGCGATGGTGGAATGGGCAATGACAGAGATTTTTAGAGATCAAAAGGTGATGAAAAGGGTACAAGATGAACTAGAAGAAATAGTAGGTCTAAACAACATCGTTGAAGAATCGCATATTCCAAAATTGAAGTACCTTGAGGCCGTGTGTAAGGAAACATTCCGTTTACATCCTCCAATACCTTTCCTACTCCCTCGAGCACCAATTAAGTCTTGTACGGTCGGAGGTTACACAGTTCCAGAAGGCGCTACTATCTTTGTAAATGTATGGGCGATACAAAGGGACCCACGACATTGGGAGAATCCATCTGAGTTCAACCCTGATCGGTTTTTGAACCGTAATGGATCTACCGAGAAATGGGACTATAGTGGTACGAATCTTACGTTTTTACCATTTGGATCTGGTAGGAGAAGGTGTCCAGGAATTCCTTTAGGTGAGAAGATGATGATGCATATTTTGGCTTCATTAATGCACTCTTTTGATTGGAAATTGCCGAATGGCGAAGAACTTGACCTCTCTGAGAGATTTGGTATTGCACTCAAGAAAAAAAAGCCGCTTGTAGCCGTCCCGACTAAAAGATTAAGTGACCTAAGCCTTTACATGTGA

**>Cnan706X**

ATGGCTGCTTCCTCCCACTCTAGTTTATGGGAAGAAGCCACCAACAACAAGCACGAAGTCGCTTTGGCCGTTTTCGTTGGTACTTTGGTTATTTTGGCTATCTCTTGGTACAAGAGATCCAACTCTAGAACCGGTAAGGGTACACCTCCATTGCCTCCAGGTCCAAAGGGTTTACCAATCGTCGGTTATCTACCATTTTTGTCTCCAAACTTGCATCACGAATTTGCTAAGATTGCCAACCAATACGGTCCAATTTTCAAGTTGTACTTGGGTTCTAAATTGCACATCGTTGTTAACTCCGCTGACTTGGCTAAGGTCGTTACTGGTGAACAAGACGAATCTTTCGCTAACAGAGACCCGCACATTGCTGGCTTGACCGCATCTTACGGTGCCTCCGATGTTGCTTGGCAAAACAATAACTCCAACAGAAGAAACTTGAGAAAGGTGTTGGTCCATGAAGTTTTGTCAAACAAGAACTTGGAAGCTTCTCACGCTTACAGAAGATCTGAAGTTCGTAAGACCATCAAGAACGTTCACGACATGATTGGTACCGCTGTTGACATCAACGAAGTTTCCTTTTCTACTGTTTTGAATATTCTTACTCACATTGTTTGGGGTAACTCTTTCGTCGAAGGTGCTAAATACCCAAATCTAGCCGCTGATATCAGAAAGGTTGTCTTGGATATTGTTGAAATTGCTGAAGGTTTGAACCTCTCCGATTTCTTCCCAATGTTGGCTAGATTCGATTTCCAAGGTGTTGAACGTCGTGTCAAAGCTCAAGTTAAGAAGTTTGACCACATTTTCGAAACCACTATTGAAGAAAGAACCAACTCCAAGTCTAAGGTCTCTGAAGAAGCTGTCAAGCAAGAAGGTAGAAAGGATTTCTTGCAAATCTTGTTGGAATTGTTAGACCAAAACACTGCTACCTCCATCAACATGACCCAATTGAAGGCTTTGGTTGTCGATATCTTCTTGGGTGGTACTGATGCTACTGCTGCCATGACTGAATGGGCTATGGCCGAAATCCTACGTAACCCAAAGGTTATGAAGAAGGTTCAAGATGAATTGGCTGAAGTCGTCGGCTTAAACAACATCGTCGAAGAATCTCATTTGCCAAAGTTGAAGTACTTGGACGCTGTTTTCAAGGAAACTTTCAGATTGCACACTCCATTGCCATTCTTATTGCCAAGAACTCCAGACAAGTCTTGTGTCGTTGGTGGTTACACCGTTCCAAAGGGTGCTACTGTCTTCTTGAACGTCTGGGCCATCCAAAGAGACCCACAAAACTGGGAAAACCCTTCTGAATTTAACCCAGAAAGATTCTTGAATAACAAAGGTTCCGAAAAGTGGGACTACTCTGGTACCAATTCCACTTACTTCCCATTCGGTTCTGGTAGAAGAAGATGTCCAGGTATTTTGTTGGGTGAAAAAATGATGATGCATATCCTCGCCTCGTTGATGCACTCCTTCGACTGGTCTTTGCCAAAGGGTGAAGAGTTAGATTTGTCAGACAAGTTCGGTATTGCTATGAAAAAGAAGATGCCATTAGTTGTCATCCCATCTTTGAGATTAAGTGACTTGTCTTTATACTCC

**>Lsal706X**

atggcttctaacgaattggctttttcagcattgttggttactttggttttggttttgatttcttggtacaagagagaaatctctaactcaagaaaggcaggtacaccaccattaccaccaggtccatacggcttgccactagtcggttatttgccattcttgggtccttccctacaccacgaattgactaagatggcccacagatacggtccaatcttcaagttgtacttgggttccaaattgcacattgttgttaattctgctgatctggctaaggttatcacttccgaacaagatgaatcctttgctaacagagctccacacattgctggtttggccacatcttacggtgctaacgacattgctttcgctgacaacaacgccaacagaagaaacttgagaaagatcttggtccacgaaatcttgtccaacgttaacttggaagcttctcacgcatacagaagaagagaagttagaaagactatcaagagtgttcatgacatgattggtatgccagttgatattaacgaaatgtctttctccactgttgttaacgtattgacctctattgtctggggtaactccatggttgaaggtaccaagcattctaacctaggtgaagaaataagaaaggttgtctctgaaatcgtcgatattgctgaaggtttgaacatctctgacttcttcccaaaattagctagattcgacttgcaaggtgtcgaacaaaagatgaagagaaagatgaagcaatttgactggattttcgaaaccaccattgaagaacgtatcaacttgaagtctacccacggtgaagatgccctaaagcacgaaggtcgtaaggatttcttgcaaatcttattggaattgaaggacaagaaatcaatcaccatgactcaattgaaggctctcgtcgttgacatcttcttgggtggtactgatgctacttctgccatggttgaatgggctatggctgagattttgaagaaccaaaaggttatgaaaaaggtccaagacgaattagccgaaatcgttggtttaaagaacatggtcgaagaatctcatttacctaaattaaagtacttgaatgctaccttcaaggaaactttcagattgcataccccactaccagtcttgttgccaagaactccatctaagagttgtatggtcggtggttacttgatcccaagagattctactgtcttcttaaatgtctgggccattcaaagagacccacaacactgggaaaacccatccgaattcaacccagaaagatttttgaactacgaaggttcaggtaagtgggactactctggtaccaactccaagtacttcccattcggttccggtcgtcgtagatgtccaggtattccattggctgaaaagatgatgttgcacatcctggcttccttgttgcactctttcgattggtctttgccgaagggtgaagaccacgatttgtttgaaaagttcggtatcgctttgaaaaagaagaagccattggttgctgttccatctccaagattgattgacttgtctttatacatgtaa

**Ancestral DNA sequences**

**>ancXY**

ATGGCTTCTAACGAATTGGCTTTTTCAGCATTGTTGGTTACTTTGGTTTTGGTTTTGATTTCTTGGTACAAGAGAGAAATCTCTAACTCAAGAAAGGCAGGTACACCACCATTGCCACCAGGTCCAAGAGGTTTACCAGTTGTTGGTTATTTGCCATTTTTAGGTCCAAATTTGCATCAAGAATTCACTAAGATGGCTCATAGATACGGTCCAATTTTTAAGTTGCATTTGGGTTCAAAGTTGCATATCGTTGTTAATTCTGCTGATTTGGCAAAAGTTGTTGCTAGAGAACAAGATGAAACTTTCGCAAACAGAAATCCACCAGTTGCTGCATTAGCTATTACATATGGTGGTCAAGATATCGCATGGTCTAACAACAACTCAAACTGGAGAAATTTGAGAAAGGTTTTGGTTCATGAAGTTTTGTCAAATAAGAATTTGGAAGCATCTAGATCTTTTAGAAGAAGAGAAGTTAGAAAGACTATTAAAAATGTTTACGAAAAGATTGGTACAGAAATTGATATTAATGAAATTGCTTTTTCTACTGAATTGAACGTTTTGACATCAATGGTTTGGGGTAAATCTTTGGTTGAAGGTGAAAAGTACTCAAATTTGGGTGACGAATTCAGAGAAGTTGTTTCTAAGATCGTTGAAATCTTGGGTGCACCAAACATCTCTGATTTCTTTCCAATCTTGGCTTGGTTCGATTTGCAAGGTGTTGAAAGAGAAATGAAAAGACAATTGAAGCAATTGGATAGAATCTTCGAATCAATTATTGAAGAAAGAATTAATTCTAATTCAACTAAATCTGAAGAAGCTGTTGAACATGAAGGTAGAAAGGATTTCTTGCAAATCTTGTTGGAATTGAAGGATCAAAAGGATGCAACTTCAATTAATATCACACAAATTAAAGCTTTGTTAGTTGATATTTTGTTAGGTGGTACTGATACTACATCTACAATGGTTGAATGGGCTATGGCAGAAATCTTGCAAAACCAAAAAGTTATGAAGAAAGTTCAAGATGAATTGGCAGAAATCGTTGGTTTGAACAACATCGTTGAAGAATCACATTTGCCAAAGTTGAAGTATTTGGATGCTGTTATTAAAGAAACTTTTAGATTACATCCACCATTGCCATTGTTAATTCCAAGATCTCCAAATCAATCATGTACTGTTGGTGGTTACACAATCCCAAAGGGTTCTACAGTTTTCTTGAATGTTTGGGCAATTCATAGAGATCCACAATACTGGGATAACCCATTGGAATTCAATCCAGAAAGATTTTTGAACAGAGAAGGTACAGATAAATGGGATTACAACGGTAACAATTTGAAGTTCTTGCCATTTGGTTCAGGTAGAAGAAGATGTCCAGGTATTCCATTGGGTGAAAAGATGTTGATGTACATCTTGGCTTCTTTGTTGCATTCATTCGATTGGTCTTTGCCAAAGGGTGAAGAACATGATTTGTCTGATAAGTTCGGTATCGCTTTGAAGAAAAGAAAGCCATTGATCGCAATTCCATCACAAAGATTACCAGATGCTTCTTTGTACATGTAA

**>ancX**

ATGGCTTCTAACGAATTGGCTTTTTCAGCATTGTTGGTTACTTTGGTTTTGGTTTTGATTTCTTGGTACAAGAGAGAAATCTCTAACTCAAGAAAGGCAGGTACACCACCATTGCCACCAGGTCCAAGAGGTTTGCCATTAGTTGGTTATTTGCCATTTTTAGGTCCAAATTTGCATCAAGAATTGACTAAGATGGCTCATAGATACGGTCCAATTTTTAAGTTGTACTTAGGTTCAAAGTTACATATTGTTGTTAATTCTGCTGATTTGGCAAAAGTTGTTACTGGTGAACAAGATGAATCATTTGCTAATAGAGCACCACATATTGCTGGTTTAGCAACATCTTATGGTGCAAATGATATCGCTTTCGCAGATAACAACGCTAACAGAAGAAATTTGAGAAAGGTTTTGGTTCATGAAGTTTTGTCAAATGTTAATTTGGAAGCTTCTCATGCATACAGAAGAAGAGAAGTTAGAAAGACAATTAAAAATGTTCATGAAATGATCGGTAACGAAGTTGATATCAACGAAATCGCATTTTCTACTGTTTTGAACGTTTTGACATCAATCGTTTGGGGTAAATCTATGGTTGAAGGTGCTAAGTACTCAAATTTGGGTGAAGAAATCAGAAAGGTTGTTTCTGGTATTGTTGAAATTGCAGGTGGTTTGAACATCTCAGATTTCTTTCCAATGTTGGCTAGATTCGATTTGCAAGGTGTTGAAAGAAAGATGAAGAGACAAATGAAGCAATTCGATAAGATCTTCGAATCTACTATTGAAGAAAGAATTAATTCTAAGTCAACAAACGTTGAAGAAGCAGTTAAGCATGAAGGTAGAAAGGATTTCTTGCAAATCTTGTTGGAATTGAAAGATCAAAAGAATGAAACTTCAATCACTATGACACAATTGAAGGCTTTGGTTGTTGATATTTTCTTGGGTGGTACTGATGCTACATCTGCAATGGTTGAATGGGCAATGACAGAAATCTTGAGAAACCAAAAAGTTATGAAGAAAGTTCAAGATGAATTGGCTGAAATCGTTGGTTTGAACAACATCGTTGAAGAATCACATTTGCCAAAGTTGAAGTATTTGGATGCAGTTTTTAAAGAAACTTTTAGATTACATCCACCATTGCCATTTTTGTTACCAAGAGCTCCAAATAAGTCATGTACTGTTGGTGGTTACACAATCCCAAAGGGTTCTACAATTTTCTTGAACGTTTGGGCTATTCAAAGAGATCCACAATACTGGGAAAATCCATCAGAATTCAATCCAGAAAGATTTTTGAACTACGAAGGTTCTGAAAAATGGGATTACTCTGGTACTAACTCAAAGTTTTTCCCATTCGGTTCTGGTAGAAGAAGATGTCCAGGTATTCCATTGGGTGAAAAGATGATGATGCATATCTTGGCTTCTTTGTTGCATTCATTCGATTGGTCTTTGCCAAAGGGTGAAGAACATGATTTGTCTGATAAGTTCGGTATCGCTTTGAAGAAAAGAAAGCCATTGATTGCAGTTCCATCTCCAAGATTATCTGATGCTTCATTGTACATGTAA

**>ancX1**

ATGGCTTCTAACGAATTGGCTTTTTCAGCATTGTTGGTTACTTTGGTTTTGGTTTTGATTTCTTGGTACAAGAGAGAAATCTCTAACTCAAGAAAGGCAGGTACACCACCATTGCCACCAGGTCCAAGAGGTTTGCCATTAGTTGGTTATTTGCCATTTTTAGGTCCAAATTTGCATCAAGAATTGACTAAGATGGCTCATAGATACGGTCCAATTTTTAAGTTGTACTTAGGTTCTAAGTTACATATTGTTGTTAATTCAGCTGATTTGGCAAAAGTTGTTACTGGTGAACAAGATGAATCTTTTGCTAATAGAGCACCACATATTGCTGGTTTGGCAACATCTTACAACGCATCAGATATCGCTTTCGCAGATAACAACGCTAACAGAAGAAAGTTGAGAAAAGTTTTGGTTCATGAAGTTTTATCTAATGTTAATTTGGAAGCTTCACATGCATACAGAAGAAGAGAAGTTAGAAAGACTATTAAAAATGTTCATGAAATCATTGGTAATGAAGTTGATATTAATGAAATCGCTTTTTCTACTGTTTTGTCAGTTTTGACATCTATCGTTTGGGGTAAATCTATGGTTAAGGGTGCTAAGTACTCAAATTTGGTTGCAGAAATGAGAAAGTTCGTTTCTGGTGTTGTTGAAATCGCTGGTGAATTGAACATCTCAGATTTCTTTCCAATGTTGGCTAGATTCGATTTCCAAGGTGTCGAAAGAAGAATGAAGAAACAAATGAAGTTGTTCGATAAGATCTTCGAATCTACAGTTGAAGAAAGAATTAATTCTAGATCAGCTATTAAAGAAGAAGCAGTTAAGGAAGAAGGTAGAAAGGATTTCTTGCAAATCTTGTTGGAATTGCAAGAACAAAACAATGAAACTTCTATCACTATGACACAAATGAAGGCTTTGGTTGTTGATATTTTCTTGGGTGGTACTGATGCTACATCAGCAATGATTGAATGGGCAATGACAGAAATCTTGAGAAACAGACAAGTTATGAAGAAAGTTCAAGATGAATTGGCTGAAATCGTTGGTTTGAACAACATCGTTGAAGAATCTCATTTGCCAAAGTTGAAGTATTTGGATGCAGTTTTTAAAGAAACTTTTAGATTACATCCACCATTGCCATTTTTGTTACCAAGAGCTCCAAATAAGTCTTGTACTGTTGGTGGTTACACAATCCCAAAGGGTTCAACAATTTTCTTGAACGTTTGGGCAATTCAAAGAGATCCACAATACTGGGAAAATCCATCTGAATTCAATCCAGAAAGATTTTTAAACTACAAAGGTTCAGAAAAATGGGATTACGCTGGTACTAATTCTAAATTTTTCCCATTGGGTTCAGGTAGAAGAAGATGTCCAGGTGTTTCTTTGGGTGAAAAGATGATGATGCATATCTTGGCATCATTGTTGCATTCTTTCGATTGGTCATTGCCAACAGGTCAAAAGTTGGATTTGTCTGATAAGTTCGGTATCGCTTTGAAGAAAAGAAAGCCATTGATTGCAGTTCCATCTCCAAGATTAAATGATGCTTCATTGTACATGTAA

**>ancX2**

ATGGCTTCTAACGAATTGGCTTTTTCAGCATTGTTGGTTACTTTGGTTTTGGTTTTGATTTCTTGGTACAAGAGAGAAATCTCTAACTCAAGAAAGGCAGGTACACCACCATTGCCACCAGGTCCAAGAGGTTTGCCATTAGTTGGTTATTTGCCATTTTTAGGTCCAAATTTGCATCAAGAATTGACTAAGATCGCTCATAGATACGGTCCAATTTTTAAGTTGTACTTAGGTTCAAAGTTACATATTGTTGTTAACTCTGCTGATTTGGCAAAAGTTATTACTGGTGAACAAGATGAATCATTTGCTAATAGAGCACCACATATTGCTGGTTTAGCAACATCTTATGGTGCTAATGATATCGCTTTCGCAGATAACAACGCAAACAGAAGAAATTTGAGAAAGGTTTTGGTTCATGAAGTCTTGTCAAATGTTAATTTGGAAGCTTCTCATGCATACAGAAGAAGAGAAGTTAGAAAGACAATTAAAAATGTTCATGATATGATTGGTATGGAAGTTGATATCAACGAAATCTCTTTTTCAACTGTTTTGAACGTTTTGACATCAATCGTTTGGGGTAAAGGCATGGTTGAAGGTGCTAAGTACTCAAATTTGGGTGAAGAAATCAGAAAGGTTGTTTCTGGTATCGTTGAAATCGCTGAAGGTTTGAACATCTCTGATTTCTTTCCAATGTTGGCAAGATTCGATTTGCAAGGTGTTGAAAGAAAGATGAAGAGACAAATGAAGCAATTCGATAAGATCTTCGAAACTACAATTGAAGAAAGAATTAATTCTAAGTCAACTAACGTTGAAGAAGCTGTTAAGCATGAAGGTAGAAAGGATTTCTTGCAAATCTTGTTGGAATTGAAGGATCAAAAGAATGGTACATCAATCACTATGACACAATTGAAGGCATTGGTTGTTGATATTTTCTTGGGTGGTACTGATGCTACATCTGCAATGGTTGAATGGGCTATGACTGAAATCTTGAGAAACCAAAAAGTTATGAAGAAAGTTCAAGATGAATTGGCAGAAATCGTTGGTTTGAACAACATCGTTGAAGAATCTCATTTGCCAAAGTTGAAGTATTTGGATGCTGTTTTTAAAGAAACTTTTAGATTACATCCACCATTGCCATTTTTGTTACCAAGAACACCAAATAAGTCATGTACTGTTGGTGGTTACACAATCCCAAAGGGTTCTACTATTTTCTTGAACGTTTGGGCAATTCAAAGAGATCCACAACATTGGGAAAATCCATCAGAATTCAATCCAGAAAGATTTTTGAACTACGAAGGTTCTGAAAAATGGGATTACTCTGGTACAAACTCAAAGTTTTTCCCATTCGGTTCTGGTAGAAGAAGATGTCCAGGTATTCCATTGGGTGAAAAGATGATGATGCATATCTTGGCTTCTTTGTTGCATTCATTCGATTGGTCTTTGCCAAAGGGTGAAGAACATGATTTGTCAGAAAAGTTCGGTATCGCTTTGAAGAAAAAGAAACCATTGGTTGCAGTTCCATCTCCAAGATTGTCTGATTTGTCATTGTACATGTAA

**>ancX3**

ATGGCTTCTAACGAATTGGCTTTTTCAGCATTGTTGGTTACTTTGGTTTTGGTTTTGATTTCTTGGTACAAGAGAGAAATCTCTAACTCAAGAAAGGCAGGTACACCACCATTGCCACCAGGTCCAAAAGGTTTGCCATTAGTTGGTTCTTTGCCATTTTTAGGTCCAAACATCCATCAAGAATTGACTAAGATCTCACATCAATACGGTCCAATTTTTAAGTTGTACTTAGGTTCTAAGTTGCATATCGTTGTTAACTCAGCTGAATTGGCAAAAGTTATTACTGCTGAACAAGATGAATCTTTTGCTAATAGAGCACCACATATTGCTGGTTTAGCAACATCATATGGTGGTAATGATATCGCTTTCGCACCAAACAACGCAAACAGAAGAAATTTGAGAAAGGTTTTGGTTCAAGAAGTTTTGTCTAACGTTAATTTGGAAGCTTCACATGCATACAGAAGACATGAAGTTAGAAAGGCTGTTAAGTACGTTTACGATAGAGTTGGTATGGATGTTGATATCAACGAAATCTCTTTTTCAACTGTTTTGAATGTTTTAACAAATATTATTTGGAGAAAAGGTTTTGTTGATGATGGTGCTAATTATGCAAATTTGTCTGAAAAGATTCAAAAAGTTATTTGTAGAATTGTTGAAATTGCTGAAGGTTTGAACATCTCAGATTTCTTTCCAATGTTGGCAAGATTCGATTTGCAAGGTGTTGAAAGAAAGATGAAGGATCAAATGAAGCAATTCGATAAGATCATCGAAACTACAATTAAAGAAAGAATGAATTCTAAGTCAACTAACGTTGAAGAAACAGTTGAACATAAGGGTAGAAAGGATTTCTTGCAAATCTTGTTGGAACATACTGATCAAAAGAATGGTACATCTATCACTATGACACAATTGAAGGCTTTGGTTGCAGATATTTTCTTGGGTGGTACTGATGCTACATCAGCAATGGTTGAATGGGCTATGACAGAAATTTTTAGAGATCAAAAAGTTATGAAGAGAGTTCAAGATGAATTGGAAGAAATCGTTGGTTTGAACAACATCGTTGAAGAATCTCATATTCCAAAGTTGAAGTATTTGGAAGCTGTTTGTAAGGAAACTTTTAGATTGCATCCACCAATTCCATTTTTGTTACCAAGAGCACCAAATAAGTCTTGTACTGTTGGTGGTTACACAGTTCCAGAAGGTGCTACAATCTTCGTTAACGTTTGGGCAATCCAAAGAGATCCAAGACATTGGGAAAATCCATCTGAATTCAATCCAGATAGATTTTTAAACTGTAATGGTTCTACTGAAAAGTGGGATTACTCAGGTACTAATTTGACATTTTTACCATTCGGTTCAGGTAGAAGAAGATGTCCAGGTATTCCATTGGGTGAAAAGATGATGATGCATATCTTGGCTTCTTTGATGCATTCATTCGATTGGAAGTTACCAAATGGTGAAGAATTGGATTTGTCTGAAAGATTCGGTATCGCTTTGAAGAAAAAGAAACCATTGGTTGCAGTTCCAACAAAAAGATTGTCTGATTTGTCATTATACATGTAA

**>ancX-16**

ATGGCATCAAACGAATTGGCTTTTTCTGCATTGTTGGTTACTTTGGTTTTGGTTTTGATCTCTTGGTACAAGAGAGAAATCTCTAATTCAAGAAAAGCTGGTACACCACCATTACCACCAGGTCCAAGAGGTTTGCCAGTTGTTGGTTATTTGCCATTTTTAGGTCCAAATTTGCATCAAGAATTCACTAAGATGGCACATAGATACGGTCCAATTTTTAAGTTGCATTTGGGTTCAAAGTTGCATATCGTTGTTAATTCTGCTGATTTGGCAAAAGTTGTTGCTAGAGAACAAGATGAAACTTTCGCAAACAGAAATCCACCAATTGCTGGTTTAGCAACATCATACGGTGCTAATGATATCGCTTTCGCAAACAACAACTCTAACTGGAGAAATTTGAGAAAGGTTTTGGTTCATGAAGTTTTGTCAAATAAGAATTTGGAAGCATCTAGATCTTTTAGAAGAAGAGAAGTTAGAAAGACTATTAAAAATGTTTACGAAAAGATTGGTACAGAAATTGATATTAATGAAATTGCTTTTTCTACTGAATTGAACGTTTTGACATCAATGGTTTGGGGTAAATCTTTGGTTGAAGGTGAAAAGTACTCAAATTTGGGTGACGAATTCAGAGAAGTTGTTTCTAAGATCGTTGAAATCGCTGGTGCACCAAACATCTCTGATTTCTTTCCAATCTTGGCATGGTTCGATTTGCAAGGTGTTGAAAGAGAAATGAAAAGACAAATGAAGCAATTCGATAGAATCTTCGAATCAATTATTGAAGAAAGAATTAATTCTAATTCAACTAAATCTGAAGAAGCTGTTGAACATGAAGGTAGAAAGGATTTCTTGCAAATCTTGTTGGAATTGAAGGATCAAAAGGATGCAACTTCAATTAATATCACACAAATTAAAGCTTTAGTTGTTGATATTTTCTTGGGTGGTACTGATGCTACATCTGCAATGGTTGAATGGGCTATGGCAGAAATCTTGCAAAACCAAAAAGTTATGAAGAAAGTTCAAGATGAATTGGCAGAAATCGTTGGTTTGAACAACATCGTTGAAGAATCACATTTGCCAAAGTTGAAGTATTTGGATGCTGTTATTAAAGAAACTTTTAGATTACATCCACCATTGCCATTGTTATTGCCAAGATCTCCAAATCAATCATGTACTGTTGGTGGTTACACAATCCCAAAGGGTTCTACAGTTTTCTTGAATGTTTGGGCTATTCATAGAGATCCACAATACTGGGATAACCCATTGGAATTCAATCCAGAAAGATTTTTGAACAGAGAAGGTACAGATAAATGGGATTACAACGGTAACAATTTGAAGTTCTTGCCATTTGGTTCAGGTAGAAGAAGATGTCCAGGTATTCCATTGGGTGAAAAGATGTTGATGTACATCTTGGCTTCTTTGTTGCATTCATTCGATTGGTCTTTACCAAAAGGTGAAGAACATGATTTGTCTGATAAGTTCGGTATCGCTTTGAAGAAAAGAAAGCCATTGATCGCAATTCCATCACAAAGATTACCAGATGCTTCTTTGTACATGTAA

**Other subfamily DNA sequences**

>CYP73A1 (XM_020846215)

atggatctggtgttggttgagaaggcgttactcggcctcttcgcggcggtgactctcgccatcgtcgtctctaagctcagcgggaagaagctcaagctcccgccagggccccttccggtgccgatcttcggcaactggctccaggtcggcgacgacctcaatcaccgcaatctcgcatccctggcaaagaagttcggcgatatccttctcctccgaatgggccagcgcaatctcgttgtcgtctcgtcgccggatcatgcccgtgaggtcctccacactcagggagtcgaatttggttcccgaactcgcaacgtcgtctttgacatcttcactggaaagggtcaggacatggtcttcactgtatacggcgagcattggcggaagatgcgccgcataatgaccgtccccttcttcaccaacaaggtcgtgcagcaataccgctatgggtgggaagatgaggccgcaagcgtcgttgaggatgtccgtaaaaaccctaagtcggccaccgagggggttgtgatccgccgccgcctgcaactcatgatgtacaacaatatgtaccgcatcatgttcgaccgaaggtttgagagcgaggacgatccccttttcatgaagctgagggcccttaataacgagcggagtcgcctcgcgcagagcttcgagtacaactatggagattttatccccattctccggccgttcctgaggggatatctcaagatctgcaaggatgtgaaagatgtgcgactcaacctattcaagaactatttcgttcaggagaggaagaaattgtcgagcacgaagccgatggataacgccggactcaagtgcgccatcgatcatatactggatgcggaaaagaagggcgagattaacgaggataacgttctctacatcgtcgaaaacatcaacgttgctgcaatcgagacaactctatggtcgatcgagtggggcatcgccgagctagtaaaccacccggaagtccagcgcaagctccgcgaagaactcgacaccgtccttggccgcggcaaccaaatcacagagcccgacacccacaagctcccctacctccaggccgtcataaaagaaactctccggcttcgcatggccatccctctcctcgtcccccacatgaatctccacgacgccaagctgggaggctacgaaatccccgccgagagcaagatcctcgtcaacgcctggtggctcgccaacaaccccgcccaatggaaaaaccccgatcagttccgccccgagcggttcctcgaagaggagtcaaaagttgaggccaacggcaacgacttccgcttcatccccttcggcgtcggccgccgcagctgccctggcatcatcctcgccctccctattctcggaatcactctcggccgcctcgtccagaactttgagctcctgccgccgccgggtatggagaagctcgatacttctgagaaaggagggcagttcagtctccatatacttaagcattacaccgtcgtcgccaagcccagggaattctaa

>CYP706A3 (NCBI Number: NM_123829)

atgacagacatttccagtctctttagaaaccgaagtcgcaaggaccaacttgactatggtttaactgtgatagttatatcaactctttgttggtgtctttggctctacgccaaatgcaaacggcggtctccaccgttgccgccgggaccttggggccttcccattatcggaaaccttccattcctccaaccggagcttcacacctacttccaagggctggctaaaaagcacggtcccattttcaaactctggctcggggccaagctcacaatcgtggtcacctcttctgaagtagcgcaagagattctcaaaacgaatgacatcatcttcgcgaaccacgatgtccctgctgtgggccctgttaacacgtatggtggtacggagatcatttggtcgccgtatggaccaaagtggcggatgctgaggaagctatgtgttaataggatactgagaaacgccatgttggattcctccactgacctccgtcgccgagagactcggcaaaccgtccggtatttggcggaccaggctcgggtcggttcaccagttaacctgggagaacaaatattcttgatgatgttgaatgttgtgacgcagatgctatggggaacaacggttaaggaagaagagagggaggttgttggagccgagttcttagaggtgattagagagatgaacgacctcctgctagtgcccaatatctccgactttttcccggtattgagccggtttgatcttcagggtttggctaagcgtatgcgaagaccggctcaaagaatggatcagatgttcgaccggatcattaaccaacggttggggatggatagggacagtagtgacgggagagctgtggattttctagacgtcttgttgaaagtcaaggatgaagaagctgaaaagacgaagttgaccatgaacgatgtcaaggccgtactcatggacatggtgctcggtggtacagacacatcactgcacgtaatagaattcgcgatggccgagctattacacaaccccgatatcatgaagagagctcaacaagaggttgacaaagttgtgggaaaagaaaaagttgtggaagaatctcacatctctaaacttccatacattctcgccattatgaaagaaactctaaggcttcacacggttgctccactccttgttcctcggcgcccgtcacaaaccaccgtggtgggcggcttcaccatccctaaagattcaaagattttcatcaacgcgtgggcaatccataggaacccgaatgtatgggagaatccactgaagtttgatcccgacaggtttctggatatgtcttatgacttcaaaggaaatgacttcaattatctaccgtttgggtctggtcgtaggatatgcgtgggaatggctatgggcgagagggttgttctttacaatctcgctacgtttttgcattcttttgattggaaaattccgcaaggagagagagtggaggtcgaggagaagtttgggatcgtgttggagctaaagaatccacttgttgccacgcccgttctaaggttgtccgatccaaatctttatctctag

>CYP706B1 (NCBI Number: XM_016849700)

atgttgcaaatagctttcagctcgtattcatggctgttgactgctagccaccagaaagatggaatgttgttcccagtagctttgtcatttttggtagccatattgggcatttcattgtggcacgtatggaccataagaaagccaaagaaagacatcgccccattaccgccgggtccccgtgggttgccaatagtgggatatcttccatatcttggaactgataatcttcacttggtgtttacagatttggctgcagcttatggtcccatctacaagctttggctcggaaacaaattatgcgtagtcattagctcggcaccactggcgaaagaagtggttcgtgacaacgacatcacattttcggagagggatcctcccgtttgtgcaaagattattacctttggcctcaatgatattgtatttgattcttacagtagtccagattggagaatgaagagaaaagtgctggtacgtgaaatgcttagccatagtagcattaaagcttgttatggtctaaggagggaacaagtgcttaaaggcgtacaaaatgttgctcaaagtgctggcaagccaattgattttggtgaaacggcatttttaacatcaatcaatgcgatgatgagcatgctgtggggtggcaaacagggaggagagcagaaaggggccgacgtttggggccaatttcgagatctcataaccgaactaatggtgattcttggaaaaccaaacgtttctgatattttcccggtgcttgcaaggtttgacatacagggattggagaaagagatgactaaaatcgttaattctttcgataagcttttcaactccatgattgaagaaagagagaactttagcaacaaattgagcaaagaagatggaaacactgaagcaaaagacttcttgcagcttcttttggaactcaagcagaagaacgatagcggaatatcgataacaatgaatcaagtcaaggccttgctcatggacattgtggtcggtggaactgatacaacatcaaccatgatggaatggacaatggctgaactaattgcaaatcctgaagcaatgaaaaaggtgaagcaagaaatagacgatgttgtcggttcggatgccgccgtcgatgagactcacttgcctaagttgcgctatctagatgctgcagtaaaggagaccttccgattgcacccaccgatgccactccttgtaccccgttgcccaggtgacttaagcaacgttggtggctatagcgtaccaaagggcaccagggtcttcttaaacatttggtgtattcagagggatccacagctttgggaaaatcctttagaattcaagcctgagaggttcttgactgatcatcaaaagctcgattatttaggaaacgattcccggtacatgccgtttggttccggaaggagaatgtgtgccggagtatctctcggtgaaaagatgttgtattcctccttggcggcaatgatccatgcttatgattggaatttggccgacggtgaagaaaatgacttgattggcttatttggaattatcatgaagaaaaagaagcctttaattcttgttcctacaccaagaccatcaaatctccagcactatatgaagtaa

>CYP706C55 (NCBI Number: MH974544)

atgtctccgtccatctccctttcccttctccacaatctcgaatcttccctctcatcgaacgatttctcgcttctgagcttactttttctcctctcgggcgctttggcaattggcttttgggcatggcgtcgttccgcagagaagaagaactcgctgccgctgccaccgggaccggccggtctccccctcgtcgggaacctgcccttcctcgacccggagctccacacctacttcgcaaccctcgcgatgacgtacggccccatcctgaagctccagcttggcaagaagctcggtatcgtcgtgacgtccccagccaccgcccgcgaggtcctcaaggataacgacgtcacgttcgccaaccgcgacgtgcccatcgctgggagggtcgcgttctacggcgggtcagacatcgtgtggaattcatacgggcccgagtggaggatgttccggaaggtgtgcggcctcaagatgctgagcaaccacgcgctggactccgtctacgagctccggcggagggaggtccgaaggaccgtggggtacttcctgctccaggccgggtcgccggtgaacgttggggagcagatgttcttgacggtgcttaacgtgatcacgagcatgttgtggggtgggacggcgcagggcgaggagaaagagagcctgggtgctgatttcaggcaggcggtgtcaagtcttacaaaacttcttgggaagccaaacatttcggacttttatccgagcctggctcgatttgatttacaagggatagagaggcagatgaaggggttggcaaagcggtttgatggaatatttcagaagatgattgagcagaggttaaagatgcagagagaaaatggaagcgagtccttggacggtggtgaggagaacaaagatttcttgcagtttctattgaacttgaaggatgaggaagatgcccagactcagctaaccacgactggcctcaaagctctgctcatggacatgttggttggtggaacagactcatcctccaacacaatcgagtttgccatggctgagatcataaataaaccaaaagttctgcaaaacatccagcaagaattggaaactgtggtaggcagaggcaatgtggtagaagaatcgcacatccctaaattgccttacttacaagctgtcatgaaagagtcgctgagattgcacccgccagttcccttgctgatcccacactgcccgagtgcgacatgcaccgtcggaggctacactgtcccgaagggctctcgggtcttcatcaatgtgtgggctatacacagggaccctttgatttggagggacccattagagttcgatcctgagaggttcttgcacagtgagggcaattacaatgagcacaacttcaattacttccctttcgggtctggaagaagaatgtgcgtgggaatattgatggcggagaggatggtgctatactcgctcgccacgctcctgcactcctttgattggaagctgcccaagggggagaagatggatctaacagagcaatttgggattgtcatgaagaagaaaaagcctctaatggctgttccgtcgccgaggttctctaattctcggctttatgaatga

>CYP706M1 (NCBI Number: JX518290)

atggacatgagcacaatatggtactactgggtgagtataatcttgggtgttttcatatttctgattgtgggtattcaaaaatggcgatcaaagaaacttcctccggggccttttgcattgcccctgttagggcatcttcatctgcttgagccaaatgtccatgaatgcctctcaaagatctctgagaaatttgggcctctcatgtccttcaaatttggcatgaaaacctcaatcatagtctcctcccctgcaatggcgaaggagattctgagagaaaatgaccagatatttgcaaatagaagcattcctgttgttgccagatgcattgcatatgatgcatctgatattctgtggagccctaatggacccagatggcgtttactcagaaaaatctgtgttaaggagctttttagccccaagagcactgaggccctgcagcctctgagaagagaggaagtgagaagaacaatggggaatatttataaggactccattaatggcgtcagtgttgatgttggggcaaaggcctttattacttcactgaatctgattacaaatatgatgtggagtacgagtactgagactggcgaaagagggggggaatttaaggatcttgttggggaacttgttcatgttcttggtgtgcctaatgcttctgatcttttcccctttcttgagagatttgatgttcaggggctttacaggaggatggagaaggtttttgtgagatttgataagatgtttgatgggattattgaggataaattgagtgggaagagtaaggagaaggattttttacagtctttgcttgatctggttgaaagaggtgtggatgaacaggatcctgatagcgttcagctcaccatgaaggatgttaaagttctcttgatggacatggtgacaggatcaacagatacaacatccaacacagtggaatgggcaatggcagagcttttacagcagccagagataatgaaaagagcccaaaaagaattggaagaagttgtagggcttgacaacatggtagaagaatgccacctgtcccaactcccatacttggacataatagtgaaggaagttctaaggctccaccctgcattgcctctgcttgcaccacacagaccagaaagggagtgtgagattggagggtacatcattccaaaggacacccaagtgctgatcaatgtgtggagcattcagaggaacccaaaagtgtggaaggagccactgttgtttgatccagagaggttttccgattcaaagtgggattacaatggaagggattttgactactttccatttgggtcagggagaagaatctgtgcagggctctccatggcaaaaataatggtgcattattcattggcttctcttctgcattcatttgattggtctcttcctgtggcggagaagcttaacatggatgagaagtatggaattgtgcttcgcaaggctgttcctcttgttgccctgcctaagcctcgtttgttgtaccctaatctctatgaatag

**Deep learning DNA sequences**

**>Design11361**

ATGGCTTCTAATGAATTGGCCTTCTCCGCTTTGTTGGTTACTTTGGTCTTGGTTCTCATTTCTTGGTACAAGAGAGAAATCTCCAATTCTAGAAAGGCTGGTACTCCACCATTGCCACCAGGTCCAAAGGGTTTACCTTTGGTCGGTTTCTTGCCATTCTTGGGCCCCAACCTGCATTTGGACTTGTTGAAGATGGCCAACAACTACGGTCCAATCTTCAAGTTATACTTGGGTTCCAACCTGCACATCGTAGTCAACTCTGCTGATTTGGCCAAGGTTGTTACCGGTGAACAAGACGAATCCTTCGCTAACCGTGCTCAACACATTGCTGGTTTGGCTACCAGTTACAACGCTTCCGACATCGCCTTCGCCGATAACAATGCTAACAGAAGAAAATTGAGAAAGGTTTTGGTCCACGAAGTTTTGTCTAATGTTAACTTGGAAGCTTCCAACGCTTACAGAAGAAGAGAAGTTAGAAAGACTATCAAGAACGTTCACGAAATTATCGGCAACGAAGTCGATATTAACGAAATTGCCTTCTCTACTGTGTTATCTGTCTTGACATCCATCGTCTTTGGTAAGTCCATGGTCAAGGGTGCTAAGTATTCTAATTTGGTTGCTGACATGAGAAAGTTCGTCTCCGGTGTTGTTGAGATTGCTGGTGGTTTGAACATCTCTGACTTCTTCCCAGTTCTTGCCAGATTCGATTTCCAAGGTGTCAAGAGAAAGATGGCAGATCAAATGAAGATGTTCGACAAGATTTTTGAAACCTCAGTCGAAGAAAGAATCAACTCGCGTTCTGCAATTATTGAAGAAACTGTTAAGCAAGAAGGTCGTAAGGACTTTTTGCAAATCTTGTTAGAACTGTTGGACCAAAACACTGAAACCTCCATCACCATGACTCAATTGAAAGCTTTGGTGGTTGACATTTTCTTGGGTGGTACCGATGCCACCTCCGCCATGGTTGAATGGGCTATGACCGAAATCTTCAGAGATAAAAAGGTCATGAAGAGAGTTCAAGACGAATTAGCTGAAGTTGTCGGTTTGAACAACATTGTTGAAGAATCTCACTTGCCAAAGTTGAAGTATCTAGATGCTGTCTTCAAGGAAACTTTCAGATTACATCCACCATTGCCTTTTCTATTGCCAAGAGCTCCAAACAAGTCTTGTACTGTCGGTGGTTACACCGTCCCAAAGGGTTCTACCATCTTTTTGAACGTCTGGGCTATCCAAAGAGACCCACAATACTGGGAAAACCCATCCGATTTCAACCCAGAAAGATTCTTAAACTACAAGGGTTCTAACAAATGGGACTACGCTGGTACTAACTTGAAATTCTTTCCATTCGGTTCTGGTCGTAGAAGATGTCCAGGTGTTTCTCTCGGTGAAAAGATGTTGATGCACATATTGGCTTCTTTGTTACACTCTTTCGACTGGTCTTTGCCAACTGGTCAAAAGCTAGACTTGTCTGATAAGTTCGGTATCACTTTAAAGAAACGTAAGCCATTGATTGCTGTTCCATCCCCAAGATTGTCTGATGCTTCCTTGTACTTGTAA

**>Design33380**

ATGGCTTCTAACGAACTGGCTTTCTCTGCTTTGTTGGTCACTTTGGTTTTGGTCTTGATCTCTTGGTACAAGAGAGAAATCTCCAACAGTCGTAAGGCTGGTACCCCACCATTACCTCCATCACCAAAGTCCTTGCCAATTGTCGGTCACTTGCCATTCTTGGGTACTGATATTCACCACGAATTGACTGAAATCTCTCACCAATACGGTCCAATCTTCAAGTTCCATCTCGGGTCAAAATTGCATATCATCATTAACTCCGCTGAATTGGCCAAGGTTATCACTGTCGAACAAGATGAATCTTTCGCTAACAGATGGCCACACATTGCCGGTATCGCCACATCTTATGGTGGTAACGACATTGCTTTTGCTCCAAACAACGCTAACTGGAGAAACTTGAGAAAGGTCTTAGTCCAAGAAGTCTTGTCTAACGTTAACTTGGAAGCTTCCCACGCTTACAGAAGACACGAAGTCAGAAAGGCTGTTAAGTACGTTTACGACAGAGTTGGTATGGATGTTGATATCAATGAAATCTCTTTTTCTACTGTTTTGAACGTCTTCTCCAACATTATCTGGAGAAAGGGTTTCGTCGACGATGGTACCAACTACGCCAATCTATCCGAAAAGATTCAAAAGGTCATCTGTCGTATTGTCGAAATTGCTGAAGGTTTGAACATCTCTGACTTCTTCCCAATGTTGGCTAGATTCGATTTGCAAGGTGTTGAACGTAAGATGAAGACCCAAATGAAGCAATTCGACAAGATCTTCGAAACCACCGTCGATGAAAGAATTAACTCTAAGCCAGCTATTTCTGAAGAAGCTGTCAAGGAAGAAGGTAGAAAAGATTTCTTGCAAATCTTGTTGGAACTTCTGGACCAAAACACTGCTACCTCCATCACCATGACTCAAATGAAGGCCTTGGTTGTTGACGTTTTTCTAGGTGGTACCGACGCTACTAGTGCTATGACTGAATGGGCCATGACCGAGATTTTGAGAAACAGACAAGTTATGAAAAAGGTTCAAGACGAATTGGCTGAAGTTGTCGGTTTGAACAACATTGTTGAAGAATCCCATCTACCAAAGTTGAAGTATTTGGACGCCGTTTTCAAGGAAACTTTCAGATTGCACCCACCTTTGCCATTCTTGTTGCCAAGAGCTCCAAACAAGTCTTGTACCGTTGGTGGTTACACTGTTCCAAAGGGTTCCACTATCTTTTTAAATGTCTGGGCTATTCAAAGAGACCCACAACACTGGACTAACCCATCTGAATTCAACCCAGAAAGATTCTTAAACAAAGGCTCTGAAAAGTGGGACTACAACGGTACCAATTCCAAGTACTTCCCTTTCGGTTCTGGTCGTAGAAGATGTCCAGGTATCCCATTGGGTGAAAAAATGATGATGCACATTTTAGCCTCTTTGATGCATTCCTTTGACTGGTCTTTACCAAGAGGTGAAGAACACGATTTATCCGACAAATTCGGTATTGCTATGAAGAAGAAGATGCCATTGGTTTTGATACCATCTCAAAGATTATCCGATCACAACTTGTACATGTAA

**>Design58683**

ATGGCTTCCAACGAATTGGCTTTCTCTGCTTTGTTGGTCACCTTGGTTTTGGTATTGATCTCTTGGTACAAGAGAGAAATCTCGAATTCCAGAAAGGCTGGTACTCCACCTTTGCCTCCAGGTCCAAAAGGTTTGCCAGTTGTCGGTTTCTTACCATTTTTAGGCCCAAACTTACATTTGGACTTCCTGACGTTAGTCCACAAGTACGGTCCAATCTTCAAGTTGTATTTAGGTTCCAACCTACATATCGTCGTTAACTCTGCCGATTTGGCCAAGGTTGTTACTGGTGAACAAGATGAAAGTTTTGCTAACCGTGCTCAACACATTGCAGGTTTGGCTACCTCTTACAACGCTTCTGATATCGCTTTCGCTGACAACAATGCCAACAGAAGAAAATTGAGAAAAGTCTTGGTTCATGAGGTTCTGTCCAACGTTAATTTGGAAGCTTCAAATGCGTTCAGACGTCGTGAAGTCAGAAAGACCATCAAGAACGTTCACGAAATCATTGGTAACGAAGTTGACATCAACGAAATTGCTTTCTCTACCGTCTTGTCCGTTTTGACCTCCATCGTTTTCGGTAAGTCCATGGTCAAGGGTGCTAAGTACTCCAACTTGGTTGCCGAGATGAGAAAATTCGTTTCTGGCGTGGTTGAAATTGCCGGTGAATTGAACATTTCCGATTTCTTTCCAATGTTGGCTAGATTTGATTTCCAAGGTGTTAAGAGAAGAATGGCCCAACAAATGAAGATCTTCGACAGAATTTTCGAAACAACTATTGAAGAAAGAACCGGTTCTACCTCCGGTATTATTGATCAAAAGGTCAAGGAAGAAGGTAGAAAGGATTTCTTGCAAATCTTGTTGGAATTATTGGACCAAAACACTGGTACCTCAATCACTATGACCCAATTGAAGGCTTTGGTCGTCGACATTTTTTTGGGTGGTACTGACGCTACTTCCGCCATGGTTGAATGGGCTATGACTGAAATATTCAGAGATAAGAAGGTTATGAAAAGGGTTCAAGACGAATTAGCTGAAGTTGTTGGTTTGCACAACATTGTCGAAGAATCTCACTTGCCAAAGTTGAAATACTTGGATGCTGTCTTCAAGGAAACCTTCAGATTGCACCCACCATTGCCTTTCTTGTTGCCAAGAGCTCCAAACAAGTCTTGTACTGTCGGTGGTTACACTGTTCCAAAGGGTTCCACTATCTTTTTGAACGTCTGGGCTATCCAAAGAGACCCAAGATACTGGGAAAACCCATCCGACTTCAACCCAGAAAGATTCTTGAACTACAAGGGTTCTGAAAAGTGGGACTACTCTGGTACCAACTTGAAGTTCTTCCCATTCGGATCTGGTAGACGTAGATGTCCAGGTGTCCCATTGGGTGAAAAGATGATGATGCACATCTTAGCTTCTTTGTTGCACTCTTTCGACTGGTCTTTGCCAACCGGTGAAAAGTTAGACCTCTCTGACAAGTTCGGTATCACTTTAAAGAAGCGTAAGCCATTGATTGCTATTCCATCTATGAGATTATCTGATGCCAGCCTGTACATGTA

**>Design6444**

ATGGCTTCTAATGAACTGGCTTTCTCCGCTTTGTTGGTTACTTTGGTCCTTGTCTTGATTTCTTGGTACAAGAGAGAAATCAGCAACTCTAGAAAGGCTGGAACTCCACCATTACCTCCAGGTCCTAAGGGTTTACCACTTGTTGGTTTCTTGCCATTCTTAGGTCCAAACTTGCATTTGGACTTTTTAACCATGGCTCACCAATACGGTCCAATCTTCAAGCTCTACTTGGGTTCCAACTTGCACATTGTCGTCAACTCCGCTGATTTGGCGAAGGTTGTTACCGGTGAACAAGACGAATCTTTCGCCAACAGAGCCCAACACATCGCCGGTTTGGCTACCTCTTACAACGCTTCCGATATTTTATTTGCTGACAACAACGCTAACAGACGTAAATTGCGTAAGGTCTTGGTTCATGAAGTGCTAAGTAATGTTAACTTGGAAGCTTCAAATGCTTACAGAAGAAGAGAAGTCAGAAAGACTATCAAGAACGTTCACGAGATCATTGGTAACGAAGTCGACATCAACGAAATTGCTTTCTCTACTGTTTTGTCTGTCCTAACTTCCATCGTCTTCGGTAAGTCCATGGTCAAGGGTGCTAAGTACTCTAACTTGGTTGCCGACATGAGAAAATTCGTTTCCGGTGTTGTTGAAATTGCTGGTGGTTTGAACATCTCTGATTTTTTCCCAATGTTGGCTAGATTCGATTTCCAAGGTGTTAAGAGAAAAATGGCTCAACAAATGAAGATGTTCGATAAGATCTTCGAATCTACCGTCGAAGAAAGAGTTGGTTCTACCTCTGGTATTATTGAAGAAGCTGTCAAGCAAGAAGGTAGAAAGGACTTCTTGCAAATCTTGTTAGAATTGTTGGACCAAAACACTGAAACCTCCATCACTATGACGCAAATGAAGGCATTGGTCGTTGACATTTTCTTGGGTGGTACCGATGCTACTTCTGCCATGGTTGAATGGGCCATGACTGAAATCTTCAGAGACAAGAAGGTTATGAAACGTGTCCAAGATGAATTGGCTGAAGTTGTCGGTTTGCACAACATTGTTGAAGAATCCCATTTACCAAAGTTGAAGTATTTGGATGCCGTTTTCAAAGAAACTTTCAGATTGCACCCACCATTGCCATTCTTATTGCCAAGAGCTCCAAACAAGTCTTGTACTGTTGGTGGTTACACCGTTCCAAAGGGTTCCACCATTTTTTTGAACGTCTGGGCTATCCAAAGAGATCCACAATACTGGGAAAACCCATCTGAATTCAACCCAGAAAGATTCTTAAACTACAAGGGATCTGAAAAGTGGGACTATACTGGTACTAACTTGAAGTTCTTCCCATTTGGTTCCGGTAGACGTAGGTGTCCAGGTGTCCCTTTGGGTGAAAAGATGATGATGCACATATTGGCCTCCCTTTTGCACTCATTTGACTGGTCTTTGCCAAACGGTCAAAAGTTGGATTTGTCTGACAAGTTCGGTATTACCCTAAAGAAGAGAAAGCCATTGATCGCTGTTCCATCCTTGAGACTGTCTGACGCTTCTTTGTACGTTTAA

**>Design84497**

ATGGCTTCTAACGAATTAGCCTTTTCTGCCTTGCTAGTGACATTAGTCCTAGTTTTGATTTCTTGGTACAAGAGAGAAATCTCCAACTCCAGAAAGGCCGGTACTCCCCCATTACCTCCAGGTCCAAGAGGTTTGCCTCTTGTCGGTTACTTGCCATTCTTGGGTCCACAACCACACCGTTCTTTGTCTGAAATCTCCCACAGATATGGTCCAATCTTCAAGCTTCAATTAGGTACCAAGTTGTGGATTGTTGTCAACTCTGCTGAATTGGCTAAGGTTATTCACGTTGAGCAAGATGAATCTTTCGCCAACAGAGCCCCACATATCGCCGGTTTAGCCACTTCTTACGGTGGTAACGATATTGCTTTCGCTCCAAACAACGCTAACCGTCGTAACTTGAGAAAATTGTTGGTTCAAGAAGTTTTATCTAACGTTAACTTGGAAGCTTCCCACGCTTACAGAAGACACGAAGTCAGAAAGGCTGTCAAGTACGTTTACGATAGAGTCGGTATGGACATTGATATCAATGAAATTAGCTTCACCACTGTCATCAACGTTTTCTTCAACATCTGGAGAATGGGTTTCATCGATGACCAATCCAATATTGGTAACTTGTTGGAAAAGATTCAAAAGGTTATCTGTAGAATCGTCGAAATTGCTGAAGGTTTGAACATTTCTGACTTCTTCCCAGTTTTGGCTAGATTCGATTTGCAAAGAGTTGAACGTAAGATGAAAGACCAAATGAAGCAATTCGACAAAATCATTGAAACTACCATCAAGGAAAGAATGAACTCCAAGTCCACTAACGTAGAAGAAACCGTCGAACAAGAAGGTAGAAAGGATTTCTTGCAAATCTTGTTGGAATTGTTGGATCAATCTACTGCAACCTCTATTACCATGACCCAATTAAAGGCTTTGGTCGTTGATGTTTTCTTGGGCGGTACCGACGCTACTTCCGCCATGGTTGAATGGGCTATGACTGAAATTTTGAGAAACAGACAGGTTATGAAGAAGGTCCAAGACGAATTGGCTCAAGTCGTCGGTTTGCACAACGTTGTTGAAGAATCCCATTTGCCAAAGTTGAAATATTTGGACGCTGTTTTCAAGGAAACTTTCAGACTTCATCCACCATTGCCATTTCTATTACCAAGAGCTCCAAACAAGTCTTGTACTGTTGGTGGTTACACTGTTCCAAAGGGTTCCACCATCTTTTTGAACGTCTGGGCTATCCAAAGAGACCCACAACACTGGACCAATCCTTCTGAATTCAATCCAGAAAGATTTTTGAACTACAAGGGTTCTGAAAAATGGGACTACGCTGGTACCAACTCTAAGTTTTTCCCATTGGGCTCCGGTCGTAGAAGATGTCCAGGTATCCCATTGGGTGAAAAGATGATGATGCACATTTTGGCTTCCTTGTTGCACAGTTTCGACTGGTCATTGCCAACTGGTCAAAAGTTGGACTTGTCTGACAAGTTCGGTATCGCTATGAAGAAAAAGAAGCCATTAGTTGTTGTCCCATCTTTGCGTTTGTCAGATTTGTCCTTGTACTCTTAA

**>Design91808**

ATGGCTTCCAACGAATTAGCTTTCTCTGCTTTGTTGGTCACTTTGGTTTTGGTTTTGATTTCTTGGTACAAGAGAGAAATCTCAAACTCCAGAAAGGCTGGTACTCCACCTCTACCACCAGGTCCAAAGGGATTGCCAATTGTCGGTTACTTGTTGTTCTTGGGCACCAACTTGCACATTCACTTCTCCAACTTATCTCAATCTTACGGTCCGATCTTCAAGTTTCACTTGGGTAACAAGTTGTGGGTTATCGTCAACACTGCTGAATTGGCTAAGACCATTGTCGTCGAGCAAGATGAATCTTTCGCTAACAGATGGCCACACATCGCTGGTTTGGCTACCTCCTACGGTGGTAACGACATTGCATTTGCTCCAAACAACGCCAACAGAAGAAACTTGAGAAAAGTATTGGTCCAAGAAGTTTTATCCAACGTTAACTTGGAAGCTTCTCATAACTACAGAAGACACGAAGTCAGAAAGGCTGTTAAGTACGTCTACGACAGAGTCGGTATGGATATTGACATCAACGAAATCTCCTTCACCACCGTTTTGAACGTTTTCTTCAACATCATCTGGAGAATGGGTTTCGAAGATGACCAAACCAATGTTGGTAACTTGTTGCAAAAGATTCAAAAGGTCATCTGTAGGATCGTTGAAATTGCTGAAGGTTTAAATATCTCTGACTTCTTCCCAGTCTTGGCCAGATTTGATGTTCAAAGAGTTGAACGTAAGATGAAGGACCAAATGAAGCAATTCGATAAGATTATTGAAACAACTATCAAGGAACGTATGAACTCTAAGTCTACTAATGTCGAAGAAACTGTTGAACAAAAAGGTCGTAAAGATTTCCTCCAAATCTTGCTTGAATTGTTGGACCAAAACAATGAAACCAGTATCACCATGACTCAAATGAAGGCCTTGGTTGTTGATATCTTCTTGGGTGGTACCGATGCTACTTCTGCCATGGTTGAATGGGCTATGACTGAAATTTTGCGTAACAGACAAGTTATGAAAAAGGTTCAAGACGAACTCGCCGAAATTGTTGGTTTGAACAACATAGTCGAAGAATCCCACTTACCAAAGTTGAAGTATTTAGACGCTGTCTTTAAGGAAACTTTCAGATTGCATCCACCACTACCTTTCTTACTTCCAAGAGCTCCAAACAAGACTTGTACTGTCGGTGGTTACACCGTTCCAAAGGGTTCCACTATCTTCTTGAACGTCTGGGCCATTCAAAGAGACCCACAATACTGGGATAACCCTTCCGAATTCAACCCAGAAAGATTCCTCAACTATAAGGGTTCTGAAAAGTGGGACTACAACGGTACCAACTTGAAATTCTTCCCATTCGGTTCTGGTAGAAGAAGATGTCCAGGTATCCCATTGGGTGAAAAAATGATGATGCATATCTTAGCCTCCTTGATGCACTCTTTCGACTGGTCTTTGCCAAGAGGTGAAGAATTGGATTTGAGCGACAAGTTTGGGATTGCTATGAAGAAGAAGAAGCCATTGGTTGTCATTCCATCTTTGCGTTTATCGGACCACAACTTGTACATGTAA

**>Design49566**

atggcttctaacgaattggctttttcagcattgttggttactttggttttggttttgatttcttggtacaagagagaaatctctaactcaagaaaggcaggtacaccaccacccctgcctccaggtccaaagggtttaccattggttggttccttgccattcttgggtccaaacatccatcaagaattgaccaagatcactcaccaatacggtccaattttcaagttatacttgggttctaaattgcacatcgttgtcaactccgctgaattggctaaggttatcaccgctgagcaagacgaatcttttgctaatagagctccacatatcgccggtttggctacttcttacggtggtaacgacattgctttcgccccaaacaacgctaacagaagaaacttgagaaaggttttagttcaagaagttttatccaacgttaatttggaagcttctcacgcttacagaagacacgaagtcagaaaggctgtcaagtacgttcacgaaagagtcggtatggaagtcgatatcaacaaaattgccttttccacagtcttgtccgttttgactaacattgtctgggctaagagcgtcgtcgatgatggtgccaactactctaatgaagttcaaaacatcatttctagagttgtcgaaattgctggtggtttgaacatttccttcttcgtcatggctcaaattgatttccaaggtgttgaaagaagaatgaagaaacaaatgaagcaattcgacaagataatcgaaatgaccattgaagaacgtatgaactcttctaagaacgaagccaagactaaggaagaaggtagaaaggattttttgcaaatcttgttggaacaacaacaacaaaacactgaaacctccatcaccatgactcaaatgaaagctttggtcgttgacatctttttgggtggtactgatgctacctctgctatggttgaatgggccatgactgaaatcttgagagaccaacaagttatgaagcgtgtccaagatgaattagaagaaattgttggcttaaacaacgtcgaagaatcccacttgccaaagttgaaatatttggacgccgtcttcaaggaaactttcagactccatccaccaattccattcttgctaccaagagctccaatcaagtcttgtaccgtgggtggttacactgttccagaaggtgctaccatcttcgttaacgtttgggctattcaacgtgacccacaaagatgggaaaacccatctgaattcaacccagaccgtttcttgaacagaaatggatctactggtaagtgggactactccggtaccaacttgaccttcttacctttcggttctggtcgtagaagatgtccaggtattccactaggtgaaaagatgatgatgcacattttggcctcacttttgcactccttcgactggaagttgccaaacggtcaaaagttggaattgtctgaaaggttcggtatcgctttgaagaagaagaagccattggtagctgttccaaccaagagattagatgttagtttgtacatgtaa

**>Design12854**

ATGGCTGCTTTGAACACCCACGGTAGTTGGTGGCCAGCTGAAGGTAACGGTGGTAAGAACGACGGTGACCTCCCATTAGCTTTGTTAGCTGTTATCACAGCTGCCTTATTGCCATTGTTATGGTACAAGAGATCCATCTCCTCTTCTCAAAACGGTGCCCCACCACTGCCGCCAGGTCCAAAGGGTTTACCAGTTGTCGGTTACCTTCCTTTCTTGGGCCCTAACTTGCACTTGGACTACTTGACCATGGTGCACCAATACGGTCCAATTTTCAAGATTTACTTGGGCTCAAACTTACATATCGTGGTCAACTCTGTCGATTTGGCCAAGGTTGTCACTGGTGAACAAGATGAATCCTTTGCTAACAGAGCTCAACACATCGCCGGTTTGGCTACATCTTACAACGCTTCCGACATCGCTTTCGCTGATAACAACGCCAACAGAAGAAAGTTGAGAAAGGTTTTGGTTCACGAAGTCTTGTCCAACGTTAACTTGGAAGCTTCTAATGCTTACAGACGTAGAGAAGTTAGAAAGACTATCAAGAACGTTCACGAAGTCATCGGTAACGAAGTCGATATCAATGAAATTTCTTTCTCTACTGTTTTGTCCGTTTTGACTTCTATCGTTTTCGGTAAGTCCATGGTTAAGGGTGCTAAGTACTCTAACCTCGCCGCTGACATTCGTAAATTTGTTTCTGGTGTCGTTGAAATTGCTGGTGGTCTAAACATCTCTGACTTCTTTCCAATGTTAGCTAGATTCGATTTCCAAGGTGTTGAACAAAGAATGAAGACCCAAATGAAGATGTTCGACAAGATTTTCGAAACCTCTGTCGAAGAAAGAATCAACTCCAGATCTGCTATCAAAGAAGAAGCCGTCAAGGAAGAAGGTAGAAAGGACTTCTTGCAAATCTTGTTGGAATTGTTGGAACAAAATACTGAGACCAGTATTACTATGACTCAAATGAAGGCTCTGGTCGTTGACGTCTTTTTGGGTGGTACCGATGCTACCAGTGCTATGGTTGAATGGGCCATGACCGAAATCTTCAGAAACAGACAAGTTATGAAAAAGGTCCAAGACGAATTGGCCGAAATCGTCGGATTGCACAACATTGTCGAAGAATCTCATTTGCCAAAGTTAAAATATTTGGACGCTGTCTTCAAGGAAACTTTCAGATTGCACCCACCATTGCCATTCTTATTGCCAAGAGCTCCAAACAAGACTTGTACCGTTGGTGGTTACACTGTTCCAAAAGGTTCAACCATTTTCTTGAACGTTTGGGCTATTCAAAGAGACCCAAAGTACTGGGACAACCCATCCGATTTCAACCCAGAACGTTTTTTGAACTACGAAGGTGAAAAATGGGATTATAATGGTACTAACCTAAAGTTCTTCCCATTCGGTTCCGGTAGAAGAAGATGTCCTGGTATCCCATTGGGTGAAAAGATGATGATGCACATTTTGGCTTCCTTGTTGCACTCTTTCAACTGGTCTCTACCAGAAGGTGAAGACCACGATTTGTCTGAAAAGTTCGGTATTGCTATGAAGAAGAAGAAGCCATTGATTGCCATTCCATCTTTGCGTTTGAGCGATCATAACTTGTACATGTAA

**>Design61021**

atggcttctaacgaattggctttttcagcattgttggttactttggttttggttttgatttcttggtacaagagagaaatctctaactcaagaaaggcaggtacaccaccattaccaccaggtccatacggcttgccactagtcggttatttgccattcttgggtccttccctacaccacgaattgactaagatggcccacagatacggtccaatcttcaagttgtacttgggttccaaattgcacattgttgttaattctgctgatctggctaaggttatcacttccgaacaagatgaatcctttgctaacagagctccacacattgctggtttggccacatcttacggtgctaacgacattgctttcgctgacaacaacgccaacagaagaaacttgagaaagatcttggtccacgaaatcttgtccaacgttaacttggaagcttctcacgcatacagaagaagagaagttagaaagactatcaagagtgttcatgacatgattggtatgccagttgatattaacgaaatgtctttctccactgttgttaacgtattgacctctattgtctggggtaactccatggttgaaggtaccaagcattctaacctaggtgaagaaataagaaaggttgtctctgaaatcgtcgatattgctgaaggtttgaacatctctgacttcttcccaaaattagctagattcgacttgcaaggtgtcgaacaaaagatgaagagaaagatgaagcaatttgactggattttcgaaaccaccattgaagaacgtatcaacttgaagtctacccacggtgaagatgccctaaagcacgaaggtcgtaaggatttcttgcaaatcttattggaattgaaggacaagaaatcaatcaccatgactcaattgaaggctctcgtcgttgacatcttcttgggtggtactgatgctacttctgccatggttgaatgggctatggctgagattttgaagaaccaaaaggttatgaaaaaggtccaagacgaattagccgaaatcgttggtttaaagaacatggtcgaagaatctcatttacctaaattaaagtacttgaatgctaccttcaaggaaactttcagattgcataccccactaccagtcttgttgccaagaactccatctaagagttgtatggtcggtggttacttgatcccaagagattctactgtcttcttaaatgtctgggccattcaaagagacccacaacactgggaaaacccatccgaattcaacccagaaagatttttgaactacgaaggttcaggtaagtgggactactctggtaccaactccaagtacttcccattcggttccggtcgtcgtagatgtccaggtattccattggctgaaaagatgatgttgcacatcctggcttccttgttgcactctttcgattggtctttgccgaagggtgaagaccacgatttgtttgaaaagttcggtatcgctttgaaaaagaagaagccattggttgctgttccatctccaagattgattgacttgtctttatacatgtaa

**>Design33105**

atggcttctaacgaattggctttttcagcattgttggttactttggttttggttttgatttcttggtacaagagagaaatctctaactcaagaaaggcaggtacaccaccattgccaccaggtccaagaggtttacctttggttggttatttgccattcttaggcccaaacttgcaccaagaattgaccaagatggctcacagatacggtccaattttcaagttgtacttgggttctaaactacacatcgttgttaattctgctgatttggctaaggttgttaccggtgaacaagacgaatctttcgcgaaccgtgctccacatattgccggtttggccacttcttacaacgcttccgatatcgccttcgctgacaacaatgccaacagaagaaaattgcgtaaggtcttggtccacgaagttttgtctaatgtgaacttggaagcttctcacgcttacagaagaagagaagttagaaagactatcaagaacgttcatgaaatcatcggtaacgaagttgacataaatgaaattgctttctcaaccgtcttgtccgtccttacctctatcgtctggggtaagtccatggttaagggtgcttccaacatgattgttgaagtcagaaagttcgtttccggtgttgtcgagattgctggggaattaaacatctctgattttttcccaatgttggctagattcgatttccaaggtgtcgaaagaagaatgaagaagcaaatgaaattgtttgacaagattttcgaatccactgttgaagaacgtatcaacagcagatctatcattaaggaagaagctgcttccaaagaagaaaaccgtagaaaggactttttgcaaatcttgttagaattacaagaacaaaacaacacttccatcaccatgactcaaatgaaggctttggttgtcgacatcttcctgggtggtaccgatgctacctccgccatgattgaatgggctatgaccgaaattttgagaaaccgtagagttatgaaaaaggtccaagatgaattggctgaaattgtcggtttgcaaaacaacgttgaagaagaaagtcatctcccaaagttaaagtacttggacgctgtcttcaaagaaactttcagattgcacccacctctacctttcttgttgccaagagccccaaacaagtcttgtactgtcggtggttacaccatcccaaagggttctactatctttttgaacgtctgggctattcaaagagacccacaatactgggaaaacccatccgaattcaacccagaacgtttcttgaactacaagggttctgaaaagtgggactacgctggtactaactctaagtttttcccattgggttctggtagaagaagatgtccaggtgtttctttaggtgaaaagatgatgatgcacatcttggcctctttgttgcactccttcgactggtccttgccaactggtcaaaagttggatctatctgacaaattcggtatcgctttgaagaagagaaagccattgattgctgttccatctccaagattgaacgatgcctccttatacatgtaa

**>Design42565**

atggcttctaacgaattggctttttcagcattgttggttactttggttttggttttgatttcttggtacaagagagaaatctctaactcaagaaaggcaggtacaccaccattgccaccaggcccaaagggtctaccattggttggttacttgcctttcttgggtccaaatttacatcacgaattcactaaggtttctcacagatacggtccaattttcaagttgtatttgggttccaagctccacattgttgttaacagcgctgatttggccaaggtcatcacttctgaacaagacgaatctttcgccaaccgtgccccacacattgctggtttggccacctcttacggtggtaacgatattgctttcgctgacaacaacgctaacagaagaaacttaaggaaggtcttggttcatgaagttttgtcaaacgtcaacttggaagcttcccacgcttacagacgtcacgaagtcagaaagactattaagtccgtccacgacatgatcggtatggaagttgatatcaacgaaatttccttctctactgttttgaacgttttgaccaacattgtttggggtaagggtttagttgaaggtaccaaatactccaacttgtctgaagaaatccggaaggttgtctacagaattgtcgaaattgctgaaggattgaacatttctgattttttcccaatgttggccagatttgatttacaaggtgtcgaaagaaagatgaaaaaacaaatgaagcaattcgatagaatcttcgaaaacactatctctgaaagaactaacaacaagaactctaaccacggtaagcaatgtttgcaaatcttgttggaattgaagaccaagaccatcaccaccacacaattgaaggctttggtcgttgacatcttcttaggtggtaccgatgctacttccgccatggttgaatgggctatgactgaaattttgagaaacaagaaggtaatgaagagagttcaagatgaattggaagaaatcgttggtttgaacaatatcgtcgaggaatcccatatcccaaagttgaagtacttggacgctgtcttcaaggaaactttcagattgcacccacctctgccttttttattgccaagagctccatctaagtcttgtactgtcggtggttacaccgttccaaaaggtgctaccatcttcctaaatgtctgggctatccaaagagacccaagacactgggaaaatccatctgaattcaacccagacagattccttaacaacaacaacggttcaactgaaaaatgggactactctggtactaacttgacttttttgccattcggttccggtagacgtagatgtccaggtatcccattaggtgaaaagatgatgatgcatatattagcttctttgatgcactccttcgactggagtttgccaaacggtgaagaacacgatttgtccgacaagttcggtattgcattgaaaaagaagaagccattggttgctattccaaccagaagattgtctgacgaaaatttgtacatgtaa

**>Design26159**

atggcttctaacgaattggctttttcagcattgttggttactttggttttggttttgatttcttggtacaagagagaaatctctaactcaagaaaggcaggtacaccaccattgccaccaggaccatacggtctgccattgttgggttatttgccttttttcttgggcccttccttgcaccacgaattgaccaagatggctcacagatacggtccaatcttcaaactgtatctaggttctaagttacacattgtcgtcaactctgctgatttggctaaggtcatcactagtgaacaagacgaatcttttgctaacagagctccacacatcgctggtctagccacttcttacggtgctaacgacattgctttcgctgacaacaacgccaaccgtagaaacttgagaaaaattttggtccatgaaattctatctaacgttaacttggaagcttcccacgcttacagaagaagagaagtgcgtaagaccatcaagtccgttcacgacatgattggtatgccagttgacattaacgaaatgtctttctctactgtcgtaaatgttttgacttccatagtctggggtaactccatggttgaaggtaccaagcattccaacttgggtgaagaaatcagaaaggttgtctccgaaattgttgatattgccgaaggtttgaacatctctgatttcttcccaaaattagctagattcgatttgcaaggtgttgaacaaaagatgaagagaaagatgaagcaattcgactggattttcgaaaccaccattgaagaaagaatcaacctgaagtctactcacggtgaagatgctctcaagcacgaaggtcgtaaggatttcttacaaatcttattggaattgaaagacaagaagtccattaccatgactcaattgaaggctttggttgtcgacatctttttaggtggtactgacgctacctccgccatggtcgaatgggccatggccgaaattcttaagaaccaaaaggttatgaaaaaagttcaagatgaattggctgaaatcgttggtttgaagaacatggttgaagaatcccatttgccaaagttgaagtacttaaacgctaccttcaaggagactttcagattgcacactccattgccagtcttgttgccaagaactcctagcaagtcttgtatggtcggtggttacttaattccaagagattccactgttttcttgaacgtctgggctatccaaagagacccacaacactgggaaaacccatctgaattcaatccagaaagattcttaaactacgaaggttccggtaagtgggactacagtggtacaaattcaaagtacttcccattcggttctggtagaagaagatgtccaggtatcccgttggcagaaaagatgatgttgcatatcttggcctctttgttgcactctttcgactggtctttgccaaagggtgaagaccacgatttgtttgaaaagttcggtattgctttgaaaaagaagaagccattagttgctgttccatctccacgtttgatcgatttgtctttgtacatgtaa

**>Design49566**

atggcttctaacgaattggctttttcagcattgttggttactttggttttggttttgatttcttggtacaagagagaaatctctaactcaagaaaggcaggtacaccaccacccctgcctccaggtccaaagggtttaccattggttggttccttgccattcttgggtccaaacatccatcaagaattgaccaagatcactcaccaatacggtccaattttcaagttatacttgggttctaaattgcacatcgttgtcaactccgctgaattggctaaggttatcaccgctgagcaagacgaatcttttgctaatagagctccacatatcgccggtttggctacttcttacggtggtaacgacattgctttcgccccaaacaacgctaacagaagaaacttgagaaaggttttagttcaagaagttttatccaacgttaatttggaagcttctcacgcttacagaagacacgaagtcagaaaggctgtcaagtacgttcacgaaagagtcggtatggaagtcgatatcaacaaaattgccttttccacagtcttgtccgttttgactaacattgtctgggctaagagcgtcgtcgatgatggtgccaactactctaatgaagttcaaaacatcatttctagagttgtcgaaattgctggtggtttgaacatttccttcttcgtcatggctcaaattgatttccaaggtgttgaaagaagaatgaagaaacaaatgaagcaattcgacaagataatcgaaatgaccattgaagaacgtatgaactcttctaagaacgaagccaagactaaggaagaaggtagaaaggattttttgcaaatcttgttggaacaacaacaacaaaacactgaaacctccatcaccatgactcaaatgaaagctttggtcgttgacatctttttgggtggtactgatgctacctctgctatggttgaatgggccatgactgaaatcttgagagaccaacaagttatgaagcgtgtccaagatgaattagaagaaattgttggcttaaacaacgtcgaagaatcccacttgccaaagttgaaatatttggacgccgtcttcaaggaaactttcagactccatccaccaattccattcttgctaccaagagctccaatcaagtcttgtaccgtgggtggttacactgttccagaaggtgctaccatcttcgttaacgtttgggctattcaacgtgacccacaaagatgggaaaacccatctgaattcaacccagaccgtttcttgaacagaaatggatctactggtaagtgggactactccggtaccaacttgaccttcttacctttcggttctggtcgtagaagatgtccaggtattccactaggtgaaaagatgatgatgcacattttggcctcacttttgcactccttcgactggaagttgccaaacggtcaaaagttggaattgtctgaaaggttcggtatcgctttgaagaagaagaagccattggtagctgttccaaccaagagattagatgttagtttgtacatgtaa

**>Design58**

atggcttctaacgaattggctttttcagcattgttggttactttggttttggttttgatttcttggtacaagagagaaatctctaactcaagaaaggcaggtacaccaccacttcctccaggtccaaagggtttgccattggttggttacttgccattcttgggtccaaacttgcaccaagaattaaccaagatggctcacagatacggtccaatcttcaagttgtatttgggttccaagttgcacatcgttgtcaactcagctgatttggccaaggttgttactggtgaacaagacgaaagtttcgctaacagagctccacatattgctggtttagctacctcttacaacgcttctgacattgctttcgcggacaacaacgccaacagacgtaagttgagaaaggtcctcgtccatgaagttttgtccaacgttaacttggaagctagtcacgcttacagaagaagagaagtcagaaagaccatcaagaacgtccacgaaatcatcggtaacgaagtcgacattaacgaaatcgccttctctactgtcttgtccgtcttgacctctattgtttggggtaagtccatggttgaaggtgctaagtactctaacttggttgctgaaatgagaaaattcgtttctggtgttgtcgaaatcgccggtgagttgaacatctccgacttcttcccaatgttggctagattcgatttccaaggtgttgaaaggagaatgaagaagcaaatgaaattgtttgacaagatctttgaatctaccgttgaagaaagaatcaactcccgtaaatctgtcgaaaaggaagattttttgcaaatcttgttggaattgcaagaacaaaacaatgaaacctccatcactatgactcaaatgaaggctttggttgtcgatatcttcttgggtggtactgatgctacttctgctatgattgaatgggccatgactgaaattttaagaaaccgtcaagttatgaagaaagttcaagatgaattggcagaaattgtcggtttgaataatattgtggaagaatcccacctaccaaaattgaagtacttagacgctgttttcaaggaaactttcagattacacccaccattgcctttcttgctcccaagagccccaaacaagtcttgtactgttggtggttacactattccaaagggttctaccattttcttgaatgtctgggctatccaaagagacccacaatactgggaaaacccatccgaattcaacccagaacgttttttaaactacaagggctctgaaaagtgggactacgctggtaccaactctaagtttttcccattaggttccggtagaagaagatgtccaggtgtttcactaggtgaaaagatgatgatgcacattttagcttccttgttgcattctttcgactggtctttgccaaccggtcaaaagttggacttgtctgataagttcggtatcgctttgaaaaagagaaagccattgattgctgtcccatctccaagattgaacgatgcctctttatacatgtaa

**> Design4779**

atggcctctaatgaattggctttctccgctttgttagttactctggtcttagtcttgatttcttggtacaagagagaaatctccaactctcgtaaggccggaactccaccattgccaccaggtccaagaggtttgccattggttggttacttgccattcttaggtccaaacttgcaccaagaattgaccaagatggctcacagatacggtccaatcttcaagttgtacttgggttctaaattacatatcgttgttaactctgctgacttggccaaagtcgtcactggtgaacaagacgaatcttttgctaaccgtgcccctcacattgctggcttggctacttcttacaacgcttccgatatcgccttcgctgacaacaacgcaaacagaagaaaattgcgtaaggtccttgtccacgaagtcttgtcaaacgtcaacttggaagcttctcacgcttacagaagaagagaggttagaaagaccatcaagaacgttcacgaaattattggtaacgaagttgacattaacgaaattgctttctcaaccgttttgagcattctcacttccattgtttggggtaagtccatggttgaaggtgctaagtacactaatttggttgctgaaatgagaaagttcgtttccggtgttgtggaaatagccggtgaattaaacatttccgattttttcccaatgttggccagattcgatttccaaggtgttgaaagaagaatgaagaagcaaatgaaattattcgacaagattttcgaatccactgttgaagaaagaatcaactcctccaagaaggaaggtagaaaggacttcctacaaatcttgttggaattgcaagaacaaaacaatgaaacctccatcaccatgactcaaatgaaggctttggttgtcgacatctttttgggtggtacagatgctacctctgctatggtcgaatgggctatgactgaaatcttgagaaacagacaagttatgaagaaagttcaagatgaattagctgaaattgtcggtttgaacaacatcgtcgaagaatctcatttgccaaagttgaaatatttggacgctgttttcaaggaaactttcagattacatccacctctgccattcttgttaccaagagctcctaacaagtcttgtaccgtaggtggttacaccattccaaagggttctactatttttttgaatgtctgggctatccaaagagacccacaatactgggaaaacccatctgaattcaacccagaaaggttcttgaactacaagggttctgaaaagtgggattatgctggtaccaactccaagttcttcccattgggttccggtcggcgtagatgtccaggtgtctctttgggtgaaaagatgatgatgcacatccttgcttccttgctacactctttcgactggtctttgccaaccggtcaaaagttggatttgtctgacaaattcggtatcgccttgaagaagagaaagccattgatcgctgtcccatccccaagattgaacgatgcctctttgtacatgtaa

**>Design3644**

atggcttctaacgaattggctttttcagcattgttggttactttggttttggttttgatttcttggtacaagagagaaatctctaactcaagaaaggcaggtacaccaccactcccaccaggtcctagaggtttgccactggttggttacttgccattcttgggtccaaacttgcaccacgaattgaccaagatggctcacagatatggtccaatcttcaagttatatcttggttctaagctacatatcgttgttaacagcgctgatttggctaaggttgtcaccggtgaacaagacgaatcttttgccaacagagatccacacatcgctggtttagctacctcatacaacgctaacgatattgcattcgctgacaacaacgccaaccggagaaaattgagaaaggttttggtccacgaagtcttatccaacgtcaacttggaagcttctcacgcttacagacgtagagaagttcgtaagacaattaagaacgttcatgaaatcatcggtaacgaagtcgacattaatgaaatcgctttctctactgttttgtcagtcttgacttccattgtttggggtaagtccatggttaagggtgctaagtacccaaacttagtcgctgaaatgagaaagttcgtttccggtgtcgttgaaatcgccggtgaattgaacatttctgacttcttcccaatgttggccagatttgatttccaaggtgttgaaagaagaatgaagaagcaaatgaaattgttcgataagatcttcgaatccactgttgaagaaagaattaactctagaaagaagattgctcgtaaggactttttgcaaatcttgttggaattgaaagaacaaaacaacgaaacctctatcaccatgacccaaatgaaggccctggtcgttgacatcttcttgggaggtaccgatgccacttctgctatgattgaatgggctatgactgaaattctcagaaacagacaagttatgaagaaggttcaagatgaattagctgaaattgtcggcttgaataacatcgtcgaagaatctcatttgccaaagttaaagtacttggatgctgttttcaaggaaactttcagattacacccacctttgccttttttgttaccaagagccccaaacaagtcttgtaccgtcggtggttacactatcccaaagggttcgactattttcttgaatgtctgggctatccaaagagacccacaatactgggaaaacccatctgaattcaacccagaaagattcttgaactacgaaggttccgagaagtgggactacgctggtactaactccaagttcttcccattgggttccggtagacgtagatgtccaggtatcccattaggtgaaaagatgatgatgcacatcttggcttccttgatgcactctttcgactggtctttgccagaaggtgaaaaattggacttgtccgataaattcggtattgccttgaagaagaaaaagccattgattgctgttccatctccaagattgaacgacgcttctttgtacatgtaa

**>Design2205**

atggcttctaacgaattggctttttcagcattgttggttactttggttttggttttgatttcttggtacaagagagaaatctctaactcaagaaaggcaggtacaccaccattgccaccaggtccaagaggtttaccactcgtaggatacttgcctttcttgggtccaaacctacatcacgaattggacaagatggctcacagatacggtccaatcttcaagttgtatttgggttctaagttgcacatcgttgtcaacagcgccgacttggcaaaggttgtcactggtgaacaagacgaatccttcgctaatcgtgctccacacattgctggtttggctacctcttacaacgcttcagacattgcgtttgctgacaacaacgccaacagaagaaagttgagaaaggtgttggttcacgaagttctatctaacgttaacttggaagcttcccacgcttacagaaggagagaagtcagaaagactattaagaacgttcatgaaattatcggtaatgaagttgacattaatgaaattgctttctctactgttttgtctgttttgacttccattgtctggggtaagtccatggttaagggtgctaagtacggtaacttggttgctgaaatgagaaaattcgtttctggtgttgttgaaatcgccggtgaattgaacatctctgattttttcccaatgttggctagatttgatttccaaggtgtcgaacgtagaatgaagaaacaaatgaagttgttcgataagatattcgaaaccaccgtcgaagaaagaaccaactcctccaagaagaaggtcaagaaggacttcttgcaagtcttgttggaattaaaggaacaaaacaacgaaacatccatcaacatgactcaaatgaaggctttggtcgtcgatatctttttgggtggtaccgacgctacttctgctatgattgaatgggccatgaccgaaatcctaagaaacaagcaagttatgaaaaaagtccaagatgaattggctgaagttgttggtttgaacaacattgttgaagagtctcacttgccaaaattgaaatacttagatgccgtcttcaaggaaactttcagattgcaccctccattgccattcctgttgccacgtgcccctaacaagtcttgtactgtcggtggttacaccatcccaaagggttccaccatcttcttaaacgtctgggctatccaaagagacccacaatactgggaaaacccaagtgaattcaacccagaaagattcttgaactacaagggttccgaaaagtgggactacgctggtactaactccaaattcttcccattgggctctggtcgtagaagatgtccaggtgtttccttgggtgaaaagatgatgatgcatatcttagcttctttattacactcttttgactggtctttgccagaaggtcaaaagcttgatttgtctgacaagttcggtattgccttgaagaagaagaagccattgatcgctattccatctccaagattgaacgatgcctctttgtacatgtaa

**>Design4129**

atggcttctaacgaattggctttttcagcattgttggttactttggttttggttttgatttcttggtacaagagagaaatctctaactcaagaaaggcaggtacaccaccattgccaccaggtccaagaggtttgccattagttggttacttgccattcttgggtccaaacttgcaccaagaattgaccaagatggctcacagatacggtccaatcttcaagttgtatttgggctccaagttgcatattgttgtcaactctgctgatttggccaaggtggtcaccggtgaacaagatgaatcttttgctaaccgtgctccacacattgccggtttggctacttcttacaacgcttcagacattgctttcgctgacaacaatgccaacagacgtaaattaagaaaagtcttggtccatgaagtcttgtccaacgttaacttggaagcctcccacgcttacagaagaagagaagttcgtaagaccattaagaacgttcacgaaatcatcggtaacgaagttgatattaacgaaatcgctttctccactgtcttatccgttctaacaagtatcgttttgaagggttctaacattgctgctgaaatgagaaagttcgtttccggtgtcgttgaaattgctggtgaattgaatatctctgattttttcccaatgttggctagatttgatttccaaggtgttgaaagaagaatgaagaagcaaatgaaattgttcgataagattttcgaatcaactgttgaagaaagaatcaactccagatctgctattaaggaggaagctgtcaaggaagaaggtagaaaggacttcttgcaaatcttattggaattgcaagaacaaaacaacgaaacctcgataaccatgacccaaatgaaggctctagttgttgacatcttcttaggtggtactgatgctacctctgccatgattgaatgggctatgactgaaattttgagaaacagacaagttatgaagaaggttcaagacgaattggcagaaattgtcggtttaaacaacattgtcgaagaatcccatttgccaaaactaaagtacttggacgccgttttcaaagaaactttcagattgcacccacctttgccttttttgttaccaagagccccaaacaagtcttgtactgtcggtggttacactatcccaaagggttccactatcttcttgaatgtctgggccatccaaagagacccacaatactgggaaaacccatctgagttcaacccagaaagattcttaaactacaagggttccgaaaagtgggactacgctggtaccaacagcaaattcttcccattgggttctggtcggagaagatgtccaggtgtctctttgggtgaaaagatgatgatgcacatcttggcttccctattgcactctttcgactggtctttgcctactggccaaaagttggacttgtctgacaagttcggtatcgctttgaagaagaggaagccattgatcgctgttccatctccacgtttgaacgatgcttctctttacatgtaa

## The Rosetta scripts and options for RosettaLigand

**ligand_dock.options**

-in:file:s ancX_HEM_AGI.pdb

-in:file:extra_res_fa inputs_CPD1/HEM.params inputs_CPD1/AGI.params

-run::preserve_header

-packing

-ex1

-ex2aro

-ex2

-no_optH false

-flip_HNQ true

-ignore_ligand_chi true

-enzdes

-cstfile inputs_CPD1/HEM.cst

-parser

-protocol inputs_CPD1/ligand_dock.xml

-out

-path:all outputs_CPD1

-nstruct 50

#-overwrite

**ligand_dock.xml**

<ROSETTASCRIPTS>

<SCOREFXNS>

<ligand_soft_rep weights="ligand_soft_rep">

</ligand_soft_rep>

<hard_rep weights="ligand">

</hard_rep>

</SCOREFXNS>

<LIGAND_AREAS>

<docking_sidechain_X chain="X" cutoff="6.0" add_nbr_radius="true" all_atom_mode="true" minimize_ligand="10"/>

<final_sidechain_X chain="X" cutoff="6.0" add_nbr_radius="true" all_atom_mode="true"/>

<final_backbone_X chain="X" cutoff="7.0" add_nbr_radius="false" all_atom_mode="true" Calpha_restraints="0.3"/>

<docking_sidechain_F chain="F" cutoff="6.0" add_nbr_radius="true" all_atom_mode="true" minimize_ligand="10"/>

<final_sidechain_F chain="F" cutoff="6.0" add_nbr_radius="true" all_atom_mode="true"/>

<final_backbone_F chain="F" cutoff="7.0" add_nbr_radius="false" all_atom_mode="true" Calpha_restraints="0.3"/>

</LIGAND_AREAS>

<INTERFACE_BUILDERS>

<side_chain_for_docking ligand_areas="docking_sidechain_X,docking_sidechain_F"/>

<side_chain_for_final ligand_areas="final_sidechain_X,final_sidechain_F"/>

<backbone ligand_areas="final_backbone_X,final_backbone_F" extension_window="3"/>

</INTERFACE_BUILDERS>

<MOVEMAP_BUILDERS>

<docking sc_interface="side_chain_for_docking" minimize_water="true"/>

<final sc_interface="side_chain_for_final" bb_interface="backbone" minimize_water="true"/>

</MOVEMAP_BUILDERS>

<SCORINGGRIDS ligand_chain="F" width="15">

<classic grid_type="ClassicGrid" weight="1.0"/>

</SCORINGGRIDS>

<SCORINGGRIDS ligand_chain="X" width="15">

<classic grid_type="ClassicGrid" weight="1.0"/>

</SCORINGGRIDS>

<MOVERS>

single movers_X

<AddOrRemoveMatchCsts name="cstadd" cst_instruction="add_new"/> add catalytic constraints

<Transform name="transform_F" chain="F" box_size="7.0" move_distance="0.2" angle="20" cycles="700" repeats="1" temperature="5"/>

<Transform name="transform_X" chain="X" box_size="8.0" move_distance="0.2" angle="20" cycles="700" repeats="1" temperature="5"/>

<AddOrRemoveMatchCsts name="cstrem" cst_instruction="remove" keep_covalent="1"/> remove constraints

<HighResDocker name="high_res_docker" cycles="6" repack_every_Nth="3" scorefxn="ligand_soft_rep" movemap_builder="docking"/>

<FinalMinimizer name="final" scorefxn="hard_rep" movemap_builder="final"/>

<AddOrRemoveMatchCsts name="cstfinadd" cst_instruction="add_pregenerated"/>

<InterfaceScoreCalculator name="add_scores" chains="X,F" scorefxn="hard_rep"/>

compound movers

<ParsedProtocol name="low_res_dock">

<Add mover_name="cstadd"/>

<Add mover_name="transform_F"/>

<Add mover_name="transform_X"/>

<Add mover_name="cstrem"/>

</ParsedProtocol>

<ParsedProtocol name="high_res_dock">

<Add mover_name="high_res_docker"/>

<Add mover_name="final"/>

<Add mover_name="cstfinadd"/>

</ParsedProtocol>

</MOVERS>

<PROTOCOLS>

<Add mover_name="low_res_dock"/>

<Add mover_name="high_res_dock"/>

<Add mover_name="add_scores"/>

</PROTOCOLS>

</ROSETTASCRIPTS>

**heme.cst**

#block 1 for covalent bond for CYS and CPD1

CST::BEGIN

TEMPLATE:: ATOM_MAP: 1 atom_name: FE1 N2 N1

TEMPLATE:: ATOM_MAP: 1 residue3: HEM

TEMPLATE:: ATOM_MAP: 2 atom_type: S ,

TEMPLATE:: ATOM_MAP: 2 residue1: C

CONSTRAINT:: distanceAB: 2.30 0.20 180.00 1

CONSTRAINT:: angle_A: 90.00 3.00 100.00 360.00

CONSTRAINT:: angle_B: 110.00 10.00 50.00 360.00

CONSTRAINT:: torsion_A: -80.00 5.00 50.00 360.00

CONSTRAINT:: torsion_B: 90.00 5.00 25.00 360.00

CONSTRAINT:: torsion_AB: -80.00 5.00 5.00 360.00

CST::END

## Reference

1. Thomas BJ, Rothstein R. Elevated recombination rates in transcriptionally active DNA. Cell. 1989;56(4):619-30.

2. Gu M, Wang M, Guo J, Shi C, Deng J, Huang L, et al. Crystal structure of CYP76AH1 in 4-PI-bound state from Salvia miltiorrhiza. Biochemical and biophysical research communications. 2019;511(4):813-9.

3. Shoji O, Kunimatsu T, Kawakami N, Watanabe Y. Highly Selective Hydroxylation of Benzene to Phenol by Wild‐type Cytochrome P450BM3 Assisted by Decoy Molecules. Angewandte Chemie International Edition. 2013;52(26):6606-10.

4. Liu X, Cheng J, Zhang G, Ding W, Duan L, Yang J, et al. Engineering yeast for the production of breviscapine by genomic analysis and synthetic biology approaches. Nature communications. 2018;9(1):1-10.
